# Supplementary material for: Analysis of the Factorial Structure and Reliability of the Social Determinants of Mental Health Questionnaire for Young Adults (SDMH)
Source: Eur J Investig Health Psychol Educ. 2025 Feb 2;15(2):18. doi: 10.3390/ejihpe15020018 (PMC11854730; doi:10.3390/ejihpe15020018)
Supplement: Supplementary file 1 [file ejihpe-15-00018-s001.zip › ejihpe-3385964-supplementary.pdf]

Supplementary Information S1

Analysis of the Factorial Structure and Reliability of the Social Determinants of Mental Health (SDMH) Questionnaire for Young Adults

Mónica Roncancio-Moreno, Rita Patricia Ocampo-Cepeda, Walther M. Zúñiga and Arcadio de Jesús Cardona-Isaza

Social Determinants of Mental Health (SDMH) Questionnaire for Young Adults (between 18 and 28 years old)

Participant data

Name: ______________________________ Date of birth: ________________________________

Structural Determinants

Structural determinants include socioeconomic position, sex and ethnicity. Socioeconomic position depends on education, occupation and economic income.

Differential approach data

Socioeconomic Level

1. Socioeconomic Level: ____________________________

Monthly Income: __

Sex, gender identity and sexual orientation.

2. Sex.

O Man (0)
O Woman (1)

Sexual orientation

3. Sexual orientation refers to the sexual, affective or erotic attraction that a person feels towards other people (of the same gender or of other genders). Nowadays, different forms of orientation are recognized (beyond heterosexual, homosexual and bisexual), including people who are not attracted to any of the genders. Regarding your sexual orientation, what do you recognize yourself as?

____

Ethnic affiliation

4. Do you identify yourself as a member of any ethnic group? Which one?

____

Territory

5. Where have you lived the majority of your life? Indicate the name of the country, state or city (as the case may be):

Vital situation

6. Who have you shared your home with most of your life?

O Your origin family (mother, father, brothers, grandparents) (1)
O Other relatives (2)
O Other people, non-relatives (3)
O Your partner and children (4)
O Just your partner (5)
O Just your children (6)
O Nobody else (7)

Education

7. What is your current education level?

O High School (1)
O A technical career (2)
O A technological career (3)
O A university career (4)

Marital status

8. What is your marital status?

O Single (1)
O Married (2)
O Free union (3)
O Divorced (4)
O Widow / Widower (5)

9. Are you currently in a relationship?

O Yes (1)
O No (2)

Health coverage

10. Do you currently have social health insurance?

O Yes (1)
O No (0)

Intermediate Determinants

Intermediate determinants involve the biological and psychosocial factors that condition health and health systems, such as living conditions, people's behaviors and barriers to adopting healthy lifestyles.

Protective factors

Perception of well-being in relationships and with the environment.

Our daily life takes place in different settings such as the neighborhood, the workplace, the educational institution, etc. These settings present us with challenges and opportunities for development; in them, we meet people with whom we establish relationships that can be cooperative and supportive or competitive and tense. The way we feel in these places and the nature of the relationships we establish play a very important role in our mental health, well-being and quality of life.

Well-being in different environments

11. For each of the following environments, indicate HOW COMFORTABLE YOU FEEL:

Environment	I feel very comfortable (6)	It doesn't generate tensions or problems for me (5)	It is indifferent to me (4)	It generates a lot of tensions and problems for me (3)	I find it uncomfortable and/or unpleasant (2)	I feel it is harmful for me (1)	Not applicable (0)
Home							
Neighborhood							
Study place							

12. For each one of these environments, indicate how good your relationships with people are:

Environment	Close and good relations (6)	Distant but good relationships (5)	Relationships barely existent (4)	Distant but bad relationships (3)	Close and bad relationships (2)	Not applicable (No relationships) (1)
Religious community						
Music, sports, recreational or civic group						

Social support expectations

13. For each one of the following people, indicate how much you think they would support you on a difficult situation.

Person/ Response	They would give me unconditional support (6)	They would support me depending on the situation (5)	They wouldn't support me (4)	They wouldn't show interest (3)	I fear that instead of supporting me, they would make things harder (2)	I don't know what their reaction would be (1)	Not applicable (0)
Couple							
Relatives							
Friends							

Coping strategies

14. When you find yourself in an overwhelming situation that causes you tension, discomfort or emotional pain, what do you tend to do?

O You try to find a solution (5)
O You postpone finding of a solution (4)
O You try to distract yourself to not think about it (3)
O You do nothing about it and wait for it to "happen" on its own. (2)
O You worry and suffer without being able to do anything else. (1)

Positive behaviors and sociocultural support

Attendance at counseling or therapy

15. Indicate how often you engage in the following behaviors (check only one option per row):

Behavior/ Frequency	Everyday (6)	Almost every day of the week (5)	One or two days per week (4)	One or two times per month (3)	Very rarely (2)	Never (1)
Sharing recreational moments with your family						
Sharing recreational moments with your friends						

Psychosocial life skills

16. Indicate how often you do or feel the following:

Situation / Frequency	Always (5)	Most of the time (4)	Half of the time (3)	Very rarely (2)	Never (1)
I know my strengths, weaknesses, aspirations and expectations; I am clear about what I want in life and the personal resources I must achieve it.					
I can "put myself in the place" of another person, understand his or her point of view and feelings to respond in a supportive way, according to the circumstances.					
I can express emotions and feelings in a way that is appropriate to different contexts and circumstances based on the recognition of the meaning and importance they have for me and for other people.					

Risk factors

Concerns and emotional distress

17. For each of the following areas of life, indicate how much they generate concern and emotional discomfort for you:

Area / Response	It generates a lot of concern and emotional discomfort (1)	It generates a lot of concern but not discomfort (2)	it doesn't cause me any concern (3)	Instead, it gives me peace of mind and well-being (4)	Not applicable (0)
Your life as a couple					
Your family life					
Your social life					
Your financial situation					

18. If there is any other aspect of your life causing concern or emotional discomfort, please briefly mention it:

19. What causes difficulty, worry, or emotional discomfort for you regarding college or school life?

At different times and under various circumstances, we are faced with situations or events that require us to put in additional effort in both the development of our daily activities and the construction of meaning and purpose in our lives (life project). Often, these events cause us pain or emotional discomfort so intense that it can last over time and have a lasting impact on the way we act, feel, or think. In mental health care, it is important to recognize the existence of such experiences and the impacts they may have caused.

Physical health issues

20. Do you suffer from any chronic health conditions (conditions that last for years and require ongoing treatment or monitoring, such as diabetes, asthma, or cancer, among others)?

O Yes (1)
O No (0)

21. Have you received an accurate diagnosis?

O Yes (3)
O No (2)
O Not applicable (0)

Functional diversity

22. Do you experience functional diversity (e.g., blindness or low vision, hearing loss, etc.)?

O Yes (1)
O No (0)

23. Please indicate which of the following functional diversity situations apply to you (you can select as many options as needed):

O Visual (1)
O Auditory (2)

O Motor (3)
O Intellectual (4)
O Other (5)
O Not applicable (0)

Mental health diagnosis

24. Have you ever been diagnosed with a mental health condition?

O Yes (1)
O No (0)

25. What diagnosis were you given?

---

26. Please indicate if you have received or are currently receiving any type of treatment (you may select as many options as necessary).

**Type of treatment / Response**	**Ongoing** (2)	**Finished** (1)	**Not applicable** (0)
Psychotherapeutic			
Pharmacological			
Other			

27. Do you feel that the treatment you received has led to any improvement or relief?

**Type of treatment / Response**	**Yes**	**No**	**Not applicable**
Psychotherapeutic			
Pharmacological			
Other			

28. Does this condition limit your ability to carry out and enjoy your daily activities?"

O All the time (1)
O Often (2)
O From time to time (3)
O Very few times (4)
O It doesn’t set any limitations (5)
O Not applicable (0)

Discomfort due to trauma

29. Have you ever faced overwhelming situations that caused you significant stress, discomfort, or emotional pain (e.g., a significant loss, being in danger, a sudden and abrupt life change, etc.)?

O Yes (1)
O No (0)

30. How long ago did these stressful situations that caused you great tension, discomfort or emotional pain occur (check only one option)?

O Many years ago (1)
O More than six months ago (2)
O In the last six months (3)
O Currently occurring (4)
O Not applicable (0)

Violence

31. Have you been a victim of any of the following forms of violence (or abuse)? (You may check as many options as you consider necessary).

O Physical (1)
O Sexual (2)
O Psychological (3)
O Digital (on social media) (4)
O Bullying (5)
O Forced displacement (6)
OI have not been a victim of any violence (0)

Suicidal behavior

32. Some people may feel like they 'can't take it anymore' or feel emotionally exhausted when facing situations that cause intense suffering (such as pain, shame, helplessness, etc.) and feel that finding a satisfactory solution is beyond their reach. Have you ever faced an overwhelming situation? (Please check as many options as you wish).

Option / Response	Yes (1)	No (0)
Have you seriously considered that ceasing to exist would be a solution?		

Drug use

33. Indicate the frequency of use for each of the following substances (please check only one option per row):

Environment / Frequency	Daily (4)	Weekly (3)	Occasionally (2)	Sporadically (1)	I don't consume this drug (0)
Alcoholic beverages (fermented or distilled)					
Non-prescription drugs (e.g., psychiatric drugs, etc.)					

34. As a result of using any of the substances listed above, have you ever experienced the following? (Please check only one option per row).

Response	Yes (2)	No (1)	Not applicable (0)
Conflicts with your family			
Conflicts with your couple			
Conflicts with your friends			
Economic problems			

Problematic use of the internet

35. Indicate how often you engage in each of the following activities (please check only one option per row):

Activity / Frequency	Daily (4)	Weekly (3)	Occasionally (2)	Sporadically (1)	I don't do this activity (0)
Play video games					
Watching sexual content on the internet					

36. As a result of engaging in the activities mentioned in the previous question, have you ever experienced the following? (Please check only one option per row).

Response	Yes (2)	No (1)	Not applicable (0)

Academic problems			
Legal problems			

Additional information

37. In your own words, how do you feel about your current mental health?

____

____

____

____

____

38. Indicate with whom you feel comfortable discussing the following mental health topics (you may check as many options as you find necessary).

Topic / People	With my couple	With relatives	With my friends	With study colleagues	With coworkers	With superiors or teachers	With people in my community (neighborhood church, etc.)	With a mental health professional	I wouldn't talk about it with anyone
What is mental health and why is it important in daily life?									
The importance of promoting emotional well-being through the establishment of healthy interpersonal relationships.									

39. Do you know the steps you need to take to access counseling or professional help for mental health concerns?

Option / Response	Yes (1)	No (0)
At your study place		
At work		
At your health care institution		
Independently		

40. Mention three activities that bring you the most joy, vitality, and/or satisfaction.

Frequency of well-being and discomfort

Experiences of well-being, psychological discomfort, and physical symptoms over the past week.

41. In the PAST WEEK, how frequently have you experienced the following?

Experience / Response	All the time (5)	Frequently (4)	Occasionally (3)	Very rarely (2)	Haven't experienced it (1)
Nervousness or fear about many things					
Concerns or distress					
Worry, uneasiness, or uncertainty					
Hope, optimism, or illusion					
Eating much more or much less than usual					
Digestive problems					

Experiences of well-being, psychological discomfort, and physical symptoms over the past six months.

42. In the PAST SIX MONTHS, how often have you experienced the following?

Experience / Response	All the time (5)	Frequently (4)	Occasionally (3)	Very rarely (2)	Haven't experienced it (1)
Nervousness or fear about many things					
Difficulty concentrating or thinking clearly					
Confusion in decision-making					
Vitality or energy					
Hope, optimism, or illusion					

Thank you for your participation!

Social Determinants of Mental Health Questionnaire for Young Adults (SDMH)

Questionary Structure

Item	Determinant	Component	Subcomponent	CFA
1	Structural Determinant	Differential approach data	Socioeconomic Level	NO
2	Structural Determinant	Differential approach data	Sex, gender identity and sexual orientation.	NO
3	Structural Determinant	Differential approach data	Gender	NO
4	Structural Determinant	Differential approach data	Sexual orientation	NO
5	Structural Determinant	Differential approach data	Ethnic affiliation	NO
6	Structural Determinant	Differential approach data	Territory	NO
7	Structural Determinant	Vital situation	Vital situation	NO
8	Structural Determinant	Vital situation	Vital situation	NO
9	Structural Determinant	Vital situation	Vital situation	NO
10	Structural Determinant	Vital situation	Education	NO
11	Structural Determinant	Vital situation	Education	NO
12	Structural Determinant	Vital situation	Education	NO
13	Structural Determinant	Vital situation	Education	NO
14	Structural Determinant	Vital situation	Education	NO
15	Structural Determinant	Vital situation	Education	NO
16	Structural Determinant	Vital situation	Career satisfaction	NO
17	Structural Determinant	Vital situation	Career satisfaction	NO
18	Structural Determinant	Vital situation	Career satisfaction	NO
19	Structural Determinant	Vital situation	Employment status	NO
20	Structural Determinant	Vital situation	Employment status	NO
21	Structural Determinant	Vital situation	Employment status	NO
22	Structural Determinant	Vital situation	People that depend on you	NO
23	Structural Determinant	Vital situation	People that depend on you	NO
24	Structural Determinant	Vital situation	Marital status	NO
25	Structural Determinant	Vital situation	Marital status	NO
26	Structural Determinant	Vital situation	Health coverage	NO
27	Intermediate Determinants	Protective factors	Well–being in different environments	YES

Item	Determinant	Component	Subcomponent	CFA
**28**	Intermediate Determinants	Protective factors	Well–being in different environments	YES
**29**	Intermediate Determinants	Protective factors	Well–being in different environments	YES
**30**	Intermediate Determinants	Protective factors	Well–being in different environments	YES
**31**	Intermediate Determinants	Protective factors	Social support expectations	NO
**32**	Intermediate Determinants	Protective factors	Coping strategies	NO
**33**	Intermediate Determinants	Protective factors	Coping strategies	NO
**34**	Intermediate Determinants	Protective factors	Coping strategies	NO
**35**	Intermediate Determinants	Protective factors	Attendance to counselling or therapy	NO
**36**	Intermediate Determinants	Positive behaviours and sociocultural support	Attendance at counselling or therapy	YES
**37**	Intermediate Determinants	Positive behaviours and sociocultural support	Psychosocial life skills	YES
**38**	Intermediate Determinants	Risk factors	Concerns and emotional distress	YES
**39**	Intermediate Determinants	Risk factors	Concerns and emotional distress	NO
**40**	Intermediate Determinants	Risk factors	Concerns and emotional distress	NO
**41**	Intermediate Determinants	Risk factors	Physical health issues	NO
**42**	Intermediate Determinants	Risk factors	Physical health issues	NO
**43**	Intermediate Determinants	Risk factors	Physical health issues	NO
**44**	Intermediate Determinants	Risk factors	Physical health issues	NO
**45**	Intermediate Determinants	Risk factors	Physical health issues	NO
**46**	Intermediate Determinants	Risk factors	Functional diversity	NO
**47**	Intermediate Determinants	Risk factors	Functional diversity	NO
**48**	Intermediate Determinants	Risk factors	Functional diversity	NO
**49**	Intermediate Determinants	Risk factors	Functional diversity	NO
**50**	Intermediate Determinants	Risk factors	Functional diversity	NO
**51**	Intermediate Determinants	Risk factors	Mental health diagnosis	NO
**52**	Intermediate Determinants	Risk factors	Mental health diagnosis	NO

Item	Determinant	Component	Subcomponent	CFA
**53**	Intermediate Determinants	Risk factors	Mental health diagnosis	NO
**54**	Intermediate Determinants	Risk factors	Mental health diagnosis	NO
**55**	Intermediate Determinants	Risk factors	Mental health diagnosis	NO
**56**	Intermediate Determinants	Risk factors	Mental health diagnosis	NO
**57**	Intermediate Determinants	Risk factors	Mental health diagnosis	NO
**58**	Intermediate Determinants	Risk factors	Discomfort due to trauma	NO
**59**	Intermediate Determinants	Risk factors	Discomfort due to trauma	NO
**60**	Intermediate Determinants	Risk factors	Discomfort due to trauma	NO
**61**	Intermediate Determinants	Risk factors	Violence	NO
**62**	Intermediate Determinants	Risk factors	Suicidal behaviour	YES
**63**	Intermediate Determinants	Risk factors	Drug use	YES
**64**	Intermediate Determinants	Risk factors	Drug use	YES
**65**	Intermediate Determinants	Risk factors	Problematic use of the internet	YES
**66**	Intermediate Determinants	Risk factors	Problematic use of the internet	YES
**67**	Intermediate Determinants	Risk factors	Additional information	NO
**68**	Intermediate Determinants	Risk factors	Additional information	NO
**69**	Intermediate Determinants	Risk factors	Additional information	NO
**70**	Intermediate Determinants	Risk factors	Additional information	NO
**71**	Intermediate Determinants	Positive behaviours and social-cultural support	Experiences of well-being, psychological discomfort, and physical symptoms over the past week.	YES
**72**	Intermediate Determinants	Positive behaviours and social-cultural support	Experiences of well-being, psychological discomfort, and physical symptoms over the past week.	YES

Table S1

Correlations between the intermediate determinants of the SDMH (n=1232)

	Variable	1	2	3	4	5	6	7	8	9	10	11	12	13	14	15	16	17	18	19	20	21
1	Well-being at home	—																				
2	Well-being in the neighborhood	0.39**	—																			
3	School wellness	0.25**	0.35**	—																		
4	Well-being at work	0.07*	0.12**	0.35**	—																	
5	Well-being in religious organizations	0.18**	0.20**	0.12**	0.27**	—																
6	Social welfare, culture and recreation	0.14**	0.25**	0.20**	0.26**	0.33**	—															
7	Expectation of social support	0.24**	0.31**	0.25**	0.25**	0.24**	0.24**	—														
8	Healthy lifestyle habits	0.25**	0.25**	0.31**	0.12**	0.22**	0.38**	0.23**	—													
9	Psychosocial skills for life	0.24**	0.21**	0.25**	0.11**	0.11**	0.18**	0.21**	0.35**	—												
10	Worry and emotional distress	–0.34**	–0.13**	0.21**	–0.01	–0.03	–0.03	–0.02	–0.27**	–0.28**	—											
11	Suicidal behavior	–0.27**	–0.14**	–0.13**	0.002	–.08**	–.07*	–0.08**	–0.18**	–0.22**	0.27**	—										
12	Drug use	–0.10**	0.04	–0.14**	.06*	—.11**	.07*	0.03	0.02	–0.07*	0.11**	0.11**	—									
13	Conflicts due to drug use	–0.12**	0.06*	0.04	.13**	–.07*	.07*	.07*	0.05	–0.04	0.16**	0.13**	0.52**	—								
14	Problematic internet use	–0.01	0.06*	0.06*	–0.01	–.11**	0.05	0.01	.13**	0.06*	–0.04	–0.02	0.28**	0.18**	—							
15	Problems due to problematic internet use	–0.16**	–0.01	0.06*	.12**	–0.02	0.01	.07**	0.03	–0.07**	.24**	.12**	0.23**	0.46**	.22**	_						
16	Emotional symptoms in the last week	–0.31**	–0.18**	–0.18**	–0.03	–0.05	–0.04	–.08**	–.28**	–0.25**	0.42**	0.38**	0.13**	0.12**	–0.03	.22**	_					
17	Physical symptoms in the last week	–0.21**	–0.10**	–0.11**	0.03	0.007	0.008	0.006	–.20**	–.013**	0.37**	0.31**	0.11**	0.10**	–.10**	.17**	.61**	_				
18	Well–being in the last week	0.21**	0.16**	0.19**	.06*	.06*	.10**	.16**	.29**	0.36**	–0.17**	–0.17**	0.02	0.07*	.19**	0.01	–.21**	–0.04	_			
19	Emotional symptoms in the past six months	–0.27**	–0.14**	–0.13**	–0.005	–0.05	–.06*	–.06*	–.26**	–0.23**	0.38*	0.37**	0.10**	0.14**	–0.09	.22**	.75**	.59**	–.14**	_		
20	Physical symptoms in the past six months	–0.19**	–.10**	–0.09**	0.01	0.007	–0.01	0.01	–.19**	–0.13**	0.35**	0.27**	0.10**	0.11**	–.08**	.18**	.54**	.75**	–.08**	.71**	_	
21	Well–being last six months	0.16**	0.11**	0.13**	0.02	0.04	0.03	0.14**	0.18**	0.26**	–0.11**	–0.12**	0.07	0.05	.11**	.06*	–.07*	–.07*	.60**	.09**	.17**	_

$^{*}p < 0.05$; $^{**}p < 0.01$

Prediction of Emotional Symptoms in the Last Six Months

A hierarchical regression was conducted to investigate whether well-being in different environments and risk and protective factors (intermediate factors in the survey) predicted negative emotional symptoms or distress in the last six months. Sex, age, and factors representing these components were included in the analysis.

The evaluated model was significant ($R^2 = 0.26$; $F = 24.716$, $p < 0.001$). The variables that contributed to the explained variance were fewer healthy lifestyle habits (β = -0.14; t = -4.957, $p < 0.001$; 95% CI = -0.28 to -0.124), higher levels of worry and emotional distress (β = 0.23; t = 8.625, $p < 0.001$; 95% CI = 0.41 to 0.66), greater suicidal behavior (β = 0.23; t = 8.930, $p < 0.001$; 95% CI = 1.47 to 2.30), drugs use (β = 0.06; t = 2.351, p = 0.019; 95% CI = 0.03 to 0.38), and problems due to problematic internet use (β = 0.15; t = 5.259, $p < 0.001$; 95% CI = 0.28 to 0.61).

We replicated the regression analysis by differentiating participants with a mental health diagnosis (n = 252) from those without one (n = 980). The variables affecting emotional symptoms and distress in the last six months were consistent across the entire group and these subgroups. The results of the regression are shown in the following table.

Table S2

Results of the multiple regression predicting emotional symptoms and distress over the last six months (n=1232)

Variables	B	95% CI para *B*		SE B	t	β	R^2	ΔR^2
		LI	LS					
							0.26	0.26
Constant	21.40***	16.15	26.662	2.677	7.997			
Gender	–0.07	–0.89	0.749	0.419	–0.174	–0.004		
Age	–0.02	–0.20	0.158	0.091	–0.235	–0.006		
Well-being at home	–0.08	–0.20	0.043	0.064	–1.291	–0.03		
Well-being in the neighborhood	–0.03	–0.13	0.059	0.049	–0.767	–0.02		
School wellness	–0.06	–0.18	0.066	0.065	–0.937	–0.02		
Well-being at work	–0.00	–0.05	0.051	0.027	–0.080	–0.00		
Well-being in religious organizations	0.03	–0.03	0.090	0.030	0.988	0.02		
Social welfare. culture and recreation	0.01	–0.04	0.064	0.027	0.393	0.01		
Expectation of social support	–0.00	–0.05	0.048	0.026	–0.108	–0.003		
Healthy lifestyle habits	–0.20	–0.28	–0.124	0.041	–4.957	–0.14***		
Psychosocial skills for life	0.002	–.05	0.059	0.029	0.058	0.002		
Worry and emotional distress	0.54	0.41	0.665	0.063	8.625	0.23***		
Suicidal behavior	1.89	1.47	2.306	.212	8.930	0.23***		
Drug use	0.20	0.03	0.384	0.089	2.351	0.06*		
Conflicts due to drug use	–0.08	–0.22	0.063	0.073	–1.100	–0.03		

Problematic internet use	–.01	–.17	.148	.082	–.163	–.004
Problems due to problematic internet use	0.45	0.28	0.619	0.086	5.259	0.15***

Note. B = Unstandardized Coefficient; CI = Confidence Interval; LL = Lower Limit; UL = Upper Limit; t = Test of variance; SE-B = Standard Error of B; β = Standardized Coefficient.

*p < 0.05. **p < 0.01. ***p < 0.001

Prediction of emotional well-being in the last six months.

A hierarchical regression was performed to investigate whether well-being in different settings and risk and protective factors (intermediate factors in the questionnaire) predicted emotional well-being in the last six months. Gender, age, and factors representing these components were included in the analysis.

The model evaluated was significant ($R^2 = 0.12$; $\Delta R^2 = 0.12$). The variables that contributed to the variance explained were as follows: well-being at home (β = 0.10; $t = 3.291$, $p < 0.001$; 95% CI = 0.04, 0.16) and school well-being (β = 0.06; $t = 2.247$, $p < 0.05$; 95% CI = 0.009, 0.13), where higher well-being in the school environment predicted better general well-being; social well-being, culture and leisure (β = -.06; $t = -1.992$, $p < 0.05$; 95% CI = - 0.05, 0. 000), where lower well-being in these areas negatively influenced general well-being; also contributing to the variance explained were the expectation of social support (β = 0.06; $t = 1.930$, $p < 0.05$; 95% CI = 0.000, 0.05); psychosocial life skills (β = 0. 21; $t = 6.987$, $p <$ .001; 95% CI = 0.07 to 0.13), which emerged as the most significant positive predictor of well-being; and problems due to problematic internet use (β = 0.08; $t = 2.744$, $p < 0.05$; 95% CI = 0.03 to 0.20).

We replicated the regression analysis by differentiating participants with a mental health diagnosis (n = 252) from those without one (n = 980). The variables affecting emotional well-being in the last six months were consistent across the entire group and these subgroups. The variables that showed the greatest effect on well-being were well- being at home and psychosocial life skills. The results of the regression are shown in the following table.

Table S3

Results of the multiple regression predicting well-being last six months (n=1232)

Variables	B	95% CI para *B* ------------------------ -		SE B	t	β	R^2	ΔR^2
		LI	LS					
							0.12	0.12
Constant	4.40***	1.768	7.048	1.34	3.276			
Gender	–0.11	–0.524	0.303	0.21	–0.524	–0.01		
Age	–0.02	–0.118	0.063	0.04	–0.600	–0.01		
Well–being at home	0.10	0.042	0.168	0.03	3.291	0.10***		
Well–being in the neighborhood	–0.02	–0.068	0.029	0.02	–0.798	–0.02		
School wellness	0.07	0.009	0.136	0.03	2.247	0.06*		
Well–being at work	–0.008	–0.035	0.019	0.014	–0.559	–.01		
Well–being in religious organizations	–0.003	–0.033	0.027	0.015	–0.199	–.006		
Social welfare. culture and recreation	–0.027	–0.054	0.000	0.014	–1.992	–.06*		
Expectation of social support	0.025	0.000	0.051	0.013	1.930	0.06*		
Healthy lifestyle habits	0.026	–0.015	0.066	0.02	1.225	0.04		
Psychosocial skills for life	0.10	0.074	0.132	0.01	6.987	0.21***		
Worry and emotional distress	0.029	–0.033	0.091	0.03	0.914	0.02		
Suicidal behavior	–0.14	–0.354	0.064	0.10	–1.363	–0.03		
Drug use	–0.08	–0.170	0.005	0.04	–1.843	–0.05		
Conflicts due to drug use	0.05	–0.019	0.125	00.03	1.441	0.04		
Problematic internet use	0.08	0.000	0.162	0.041	1.961	0.05		
Problems due to problematic internet use	0.11	0.034	0.203	0.043	2.744	0.08*		

Note. B = Unstandardized Coefficient; CI = Confidence Interval; LL = Lower Limit; UL = Upper Limit; *t* = Test of variance; SE-B = Standard Error of B; β = Standardized Coefficient.

*p < 0.05. **p < 0.01. ***p < 0.001

Supplementary Information S2

Analysis of the Factorial Structure and Reliability of the Social Determinants of Mental Health Questionnaire for Young Adults (SDMH)

Mónica Roncancio-Moreno, Rita Patricia Ocampo-Cepeda, Walther M. Zúñiga and Arcadio de Jesús Cardona-Isaza

CONFIRMATORY FACTOR ANALYSIS OF ENVIRONMENTS

C:\Users\media\Dropbox\Mi PC (LAPTOP-LCRG33R5)\Desktop\2024\Articulos 2024\Salud mental 2024 1\Resultados junio final\Entornos.amw

Analysis Summary

Date and Time

Date: sábado, 22 de junio de 2024
Time: 6:11:02 a. m.

Title

Entornos: sábado, 22 de junio de 2024 6:11 a. m.

Groups

Group number 1 (Group number 1)

Notes for Group (Group number 1)

The model is recursive.
Sample size = 1232

Variable Summary (Group number 1)

Your model contains the following variables (Group number 1)

Observed, endogenous variables
ENTHOB
RELHOB
CONFHOB
SOLHOB
SOLREL
CONFREL
RELREL
ENTREL
ENTHG
RELHOG
CONFLHOG
SOLHGR
ENTTBA
RELIBAR
CONFBARR
SOLBAR
ENTTRAB
RELIOTRB
CONFTRA
SOLTRAB
ENTUNI
RELUNIVE
CONFCUNI
SOLUNIV
Unobserved, exogenous variables
F1

e1
e2
e3
e4
F2
e5
e6
e7
e8
F3
e9
e10
e11
e12
F4
e13
e14
e15
e16
F6
e21
e22
e23
e24
F7
e25
e26
e27
e28

Variable counts (Group number 1)

Number of variables in your model:	54
Number of observed variables:	24
Number of unobserved variables:	30
Number of exogenous variables:	30
Number of endogenous variables:	24

Parameter Summary (Group number 1)

	Weights	Covariances	Variances	Means	Intercepts	Total
Fixed	30	0	0	0	0	30
Labeled	0	0	0	0	0	0
Unlabeled	18	18	30	0	0	66
Total	48	18	30	0	0	96

Models

Default model (Default model)

Notes for Model (Default model)

Computation of degrees of freedom (Default model)

Number of distinct sample moments:	300
Number of distinct parameters to be estimated:	66
Degrees of freedom (300 - 66):	234

Result (Default model)

Minimum was achieved
Chi-square = 2127,483
Degrees of freedom = 234
Probability level = ,000

Group number 1 (Group number 1 - Default model)

Estimates (Group number 1 - Default model)

Scalar Estimates (Group number 1 - Default model)

Maximum Likelihood Estimates

Regression Weights: (Group number 1 - Default model)

			Estimate	S.E.	C.R.	P	Label
ENTHOB	<---	F1	1.000				
RELHOB	<---	F1	1.027	.018	56.876	***	
CONFHOB	<---	F1	.738	.017	42.263	***	
SOLHOB	<---	F1	.567	.018	31.263	***	
SOLREL	<---	F2	1.000				
CONFREL	<---	F2	1.422	.055	26.029	***	
RELREL	<---	F2	1.938	.067	28.779	***	
ENTREL	<---	F2	1.795	.067	26.653	***	
ENTHG	<---	F3	1.000				
RELHOG	<---	F3	.903	.054	16.597	***	
CONFLHOG	<---	F3	.799	.052	15.236	***	
SOLHGR	<---	F3	.936	.062	15.141	***	
ENTTBA	<---	F4	1.000				
RELIBAR	<---	F4	2.320	.199	11.646	***	
CONFBARR	<---	F4	1.339	.134	9.985	***	
SOLBAR	<---	F4	1.791	.167	10.757	***	
ENTTRAB	<---	F6	1.000				
RELIOTRB	<---	F6	1.089	.020	53.959	***	
CONFTRA	<---	F6	.775	.018	44.154	***	
SOLTRAB	<---	F6	.695	.020	35.638	***	
ENTUNI	<---	F7	1.000				
RELUNIVE	<---	F7	.805	.094	8.522	***	
CONFCUNI	<---	F7	.896	.111	8.042	***	
SOLUNIV	<---	F7	1.127	.146	7.704	***	

Standardized Regression Weights: (Group number 1 - Default model)

			Estimate
ENTHOB	<---	F1	.914
RELHOB	<---	F1	.954
CONFHOB	<---	F1	.812
SOLHOB	<---	F1	.666
SOLREL	<---	F2	.644
CONFREL	<---	F2	.774
RELREL	<---	F2	.930
ENTREL	<---	F2	.829
ENTHG	<---	F3	.660
RELHOG	<---	F3	.675
CONFLHOG	<---	F3	.579
SOLHGR	<---	F3	.574
ENTTBA	<---	F4	.455
RELIBAR	<---	F4	.670
CONFBARR	<---	F4	.450
SOLBAR	<---	F4	.520
ENTTRAB	<---	F6	.898
RELIOTRB	<---	F6	.950
CONFTRA	<---	F6	.865
SOLTRAB	<---	F6	.755
ENTUNI	<---	F7	.475
RELUNIVE	<---	F7	.448
CONFCUNI	<---	F7	.398
SOLUNIV	<---	F7	.346

Covariances: (Group number 1 - Default model)

			Estimate	S.E.	C.R.	P	Label
F1	<-->	F2	.981	.100	9.771	***	
F1	<-->	F3	.295	.076	3.873	***	
F1	<-->	F4	.424	.063	6.688	***	
F1	<-->	F6	1.366	.172	7.954	***	
F2	<-->	F3	.227	.038	5.995	***	

			Estimate	S.E.	C.R.	P	Label
F2	<-->	F4	.197	.031	6.446	***	
F2	<-->	F6	.570	.083	6.881	***	
F3	<-->	F4	.258	.030	8.525	***	
F3	<-->	F6	.127	.066	1.923	.054	
F4	<-->	F6	.182	.050	3.618	***	
F1	<-->	F7	.340	.071	4.763	***	
F2	<-->	F7	.060	.032	1.850	.064	
F3	<-->	F7	.243	.033	7.369	***	
F4	<-->	F7	.227	.030	7.672	***	
F6	<-->	F7	.096	.060	1.616	.106	
e4	<-->	e5	1.129	.074	15.247	***	
e3	<-->	e6	.810	.063	12.897	***	
e24	<-->	e28	.981	.082	11.915	***	

Correlations: (Group number 1 - Default model)

			Estimate
F1	<-->	F2	.333
F1	<-->	F3	.136
F1	<-->	F4	.283
F1	<-->	F6	.248
F2	<-->	F3	.222
F2	<-->	F4	.280
F2	<-->	F6	.221
F3	<-->	F4	.495
F3	<-->	F6	.067
F4	<-->	F6	.138
F1	<-->	F7	.216
F2	<-->	F7	.081
F3	<-->	F7	.445
F4	<-->	F7	.604
F6	<-->	F7	.070
e4	<-->	e5	.509
e3	<-->	e6	.446
e24	<-->	e28	.386

Variances: (Group number 1 - Default model)

	Estimate	S.E.	C.R.	*p*	Label
F1	6.270	.304	20.619	***	
F2	1.383	.103	13.389	***	
F3	.757	.069	10.921	***	
F4	.359	.052	6.880	***	
F6	4.831	.241	20.043	***	
F7	.393	.063	6.209	***	
e1	1.239	.077	16.189	***	
e2	.654	.065	10.021	***	
e3	1.759	.080	21.965	***	
e4	2.519	.106	23.711	***	
e5	1.955	.084	23.187	***	
e6	1.870	.089	21.082	***	
e7	.817	.080	10.161	***	
e8	2.024	.106	19.096	***	
e9	.982	.055	17.845	***	
e10	.737	.043	17.263	***	
e11	.955	.047	20.298	***	
e12	1.349	.066	20.422	***	
e13	1.376	.063	21.795	***	
e14	2.370	.156	15.236	***	
e15	2.532	.116	21.872	***	
e16	3.108	.152	20.471	***	
e21	1.162	.064	18.077	***	
e22	.624	.056	11.197	***	
e23	.980	.048	20.263	***	

	Estimate	S.E.	C.R.	*p*	Label
e24	1.759	.077	22.876	***	
e25	1.349	.071	19.094	***	
e26	1.013	.051	19.902	***	
e27	1.677	.079	21.172	***	
e28	3.669	.165	22.286	***	

Matrices (Group number 1 - Default model)

Total Effects (Group number 1 - Default model)

	F7	F6	F4	F3	F2	F1
SOLUNIV	1.127	.000	.000	.000	.000	.000
CONFCUNI	.896	.000	.000	.000	.000	.000
RELUNIVE	.805	.000	.000	.000	.000	.000
ENTUNI	1.000	.000	.000	.000	.000	.000
SOLTRAB	.000	.695	.000	.000	.000	.000
CONFTRA	.000	.775	.000	.000	.000	.000
RELIOTRB	.000	1.089	.000	.000	.000	.000
ENTTRAB	.000	1.000	.000	.000	.000	.000
SOLBAR	.000	.000	1.791	.000	.000	.000
CONFBARR	.000	.000	1.339	.000	.000	.000
RELIBAR	.000	.000	2.320	.000	.000	.000
ENTTBA	.000	.000	1.000	.000	.000	.000
SOLHGR	.000	.000	.000	.936	.000	.000
CONFLHOG	.000	.000	.000	.799	.000	.000
RELHOG	.000	.000	.000	.903	.000	.000
ENTHG	.000	.000	.000	1.000	.000	.000
ENTREL	.000	.000	.000	.000	1.795	.000
RELREL	.000	.000	.000	.000	1.938	.000
CONFREL	.000	.000	.000	.000	1.422	.000
SOLREL	.000	.000	.000	.000	1.000	.000
SOLHOB	.000	.000	.000	.000	.000	.567
CONFHOB	.000	.000	.000	.000	.000	.738
RELHOB	.000	.000	.000	.000	.000	1.027
ENTHOB	.000	.000	.000	.000	.000	1.000

Standardized Total Effects (Group number 1 - Default model)

	F7	F6	F4	F3	F2	F1
SOLUNIV	.346	.000	.000	.000	.000	.000
CONFCUNI	.398	.000	.000	.000	.000	.000
RELUNIVE	.448	.000	.000	.000	.000	.000
ENTUNI	.475	.000	.000	.000	.000	.000
SOLTRAB	.000	.755	.000	.000	.000	.000
CONFTRA	.000	.865	.000	.000	.000	.000
RELIOTRB	.000	.950	.000	.000	.000	.000
ENTTRAB	.000	.898	.000	.000	.000	.000
SOLBAR	.000	.000	.520	.000	.000	.000
CONFBARR	.000	.000	.450	.000	.000	.000
RELIBAR	.000	.000	.670	.000	.000	.000
ENTTBA	.000	.000	.455	.000	.000	.000
SOLHGR	.000	.000	.000	.574	.000	.000
CONFLHOG	.000	.000	.000	.579	.000	.000
RELHOG	.000	.000	.000	.675	.000	.000
ENTHG	.000	.000	.000	.660	.000	.000
ENTREL	.000	.000	.000	.000	.829	.000
RELREL	.000	.000	.000	.000	.930	.000
CONFREL	.000	.000	.000	.000	.774	.000
SOLREL	.000	.000	.000	.000	.644	.000
SOLHOB	.000	.000	.000	.000	.000	.666
CONFHOB	.000	.000	.000	.000	.000	.812
RELHOB	.000	.000	.000	.000	.000	.954
ENTHOB	.000	.000	.000	.000	.000	.914

Direct Effects (Group number 1 - Default model)

	F7	F6	F4	F3	F2	F1
SOLUNIV	1.127	.000	.000	.000	.000	.000
CONFCUNI	.896	.000	.000	.000	.000	.000
RELUNIVE	.805	.000	.000	.000	.000	.000
ENTUNI	1.000	.000	.000	.000	.000	.000
SOLTRAB	.000	.695	.000	.000	.000	.000
CONFTRA	.000	.775	.000	.000	.000	.000
RELIOTRB	.000	1.089	.000	.000	.000	.000
ENTTRAB	.000	1.000	.000	.000	.000	.000
SOLBAR	.000	.000	1.791	.000	.000	.000
CONFBARR	.000	.000	1.339	.000	.000	.000
RELIBAR	.000	.000	2.320	.000	.000	.000
ENTTBA	.000	.000	1.000	.000	.000	.000
SOLHGR	.000	.000	.000	.936	.000	.000
CONFLHOG	.000	.000	.000	.799	.000	.000
RELHOG	.000	.000	.000	.903	.000	.000
ENTHG	.000	.000	.000	1.000	.000	.000
ENTREL	.000	.000	.000	.000	1.795	.000
RELREL	.000	.000	.000	.000	1.938	.000
CONFREL	.000	.000	.000	.000	1.422	.000
SOLREL	.000	.000	.000	.000	1.000	.000
SOLHOB	.000	.000	.000	.000	.000	.567
CONFHOB	.000	.000	.000	.000	.000	.738
RELHOB	.000	.000	.000	.000	.000	1.027
ENTHOB	.000	.000	.000	.000	.000	1.000

Standardized Direct Effects (Group number 1 - Default model)

	F7	F6	F4	F3	F2	F1
SOLUNIV	.346	.000	.000	.000	.000	.000
CONFCUNI	.398	.000	.000	.000	.000	.000
RELUNIVE	.448	.000	.000	.000	.000	.000
ENTUNI	.475	.000	.000	.000	.000	.000
SOLTRAB	.000	.755	.000	.000	.000	.000
CONFTRA	.000	.865	.000	.000	.000	.000
RELIOTRB	.000	.950	.000	.000	.000	.000
ENTTRAB	.000	.898	.000	.000	.000	.000
SOLBAR	.000	.000	.520	.000	.000	.000
CONFBARR	.000	.000	.450	.000	.000	.000
RELIBAR	.000	.000	.670	.000	.000	.000
ENTTBA	.000	.000	.455	.000	.000	.000
SOLHGR	.000	.000	.000	.574	.000	.000
CONFLHOG	.000	.000	.000	.579	.000	.000
RELHOG	.000	.000	.000	.675	.000	.000
ENTHG	.000	.000	.000	.660	.000	.000
ENTREL	.000	.000	.000	.000	.829	.000
RELREL	.000	.000	.000	.000	.930	.000
CONFREL	.000	.000	.000	.000	.774	.000
SOLREL	.000	.000	.000	.000	.644	.000
SOLHOB	.000	.000	.000	.000	.000	.666
CONFHOB	.000	.000	.000	.000	.000	.812
RELHOB	.000	.000	.000	.000	.000	.954
ENTHOB	.000	.000	.000	.000	.000	.914

Covariances: (Group number 1 - Default model)

	M.I.	Par Change
e28 <--> F4	14.298	.133
e28 <--> F3	5.834	-.119
e27 <--> F3	9.048	.109

			M.I.	Par Change
e27	<-->	e28	5.864	-.171
e26	<-->	F4	6.190	-.050
e25	<-->	e26	7.854	.105
e24	<-->	F7	6.287	-.077
e24	<-->	F3	7.878	.097
e23	<-->	e27	83.448	.379
e23	<-->	e25	5.526	-.090
e23	<-->	e24	4.632	.084
e22	<-->	e27	22.584	-.201
e22	<-->	e26	15.179	.130
e22	<-->	e25	4.456	-.082
e21	<-->	F4	4.229	-.048
e21	<-->	e26	5.140	-.084
e21	<-->	e25	39.728	.272
e21	<-->	e24	4.335	-.092
e16	<-->	e28	91.625	.941
e16	<-->	e26	5.436	-.133
e16	<-->	e25	24.060	-.325
e16	<-->	e24	23.301	.331
e15	<-->	F7	22.516	.190
e15	<-->	F2	4.730	-.116
e15	<-->	e27	115.090	.685
e15	<-->	e23	41.225	.327
e15	<-->	e22	9.897	-.164
e14	<-->	F7	17.391	-.178
e14	<-->	e27	26.167	-.351
e14	<-->	e23	4.065	-.110
e14	<-->	e22	17.628	.235
e14	<-->	e21	8.079	-.176
e14	<-->	e16	14.525	.355
e13	<-->	F7	8.150	.084
e13	<-->	F4	8.216	-.065
e13	<-->	F3	12.632	.117
e13	<-->	e28	8.131	-.183
e13	<-->	e25	53.259	.316
e13	<-->	e24	5.279	-.103
e13	<-->	e21	11.614	.145
e13	<-->	e16	20.283	-.293
e12	<-->	e28	32.244	.371
e12	<-->	e25	7.478	-.120
e12	<-->	e24	23.164	.219
e12	<-->	e23	4.381	-.080
e12	<-->	e16	55.733	.496
e12	<-->	e15	5.290	-.135
e12	<-->	e13	7.851	-.122
e11	<-->	F7	15.838	.101
e11	<-->	e27	81.409	.364
e11	<-->	e23	49.669	.227
e11	<-->	e22	17.281	-.137
e11	<-->	e15	37.344	.303
e11	<-->	e14	12.782	-.190
e10	<-->	F7	5.969	-.057
e10	<-->	F4	7.155	-.049
e10	<-->	e28	14.754	-.196
e10	<-->	e26	5.617	.070
e10	<-->	e23	8.341	-.086
e10	<-->	e22	8.383	.088
e10	<-->	e16	24.841	-.258
e10	<-->	e15	9.698	-.143
e10	<-->	e14	9.346	.151
e9	<-->	F4	5.551	.050
e9	<-->	e28	9.839	-.182
e9	<-->	e26	10.135	-.107
e9	<-->	e25	18.863	.170

			M.I.	Par Change
e9	<-->	e23	6.875	-.089
e9	<-->	e21	9.191	.117
e9	<-->	e16	8.020	-.167
e9	<-->	e13	66.973	.316
e9	<-->	e12	13.853	-.143
e9	<-->	e10	5.714	.071
e8	<-->	F6	14.618	.371
e8	<-->	F4	9.168	-.091
e8	<-->	e26	6.753	-.124
e8	<-->	e25	17.370	.231
e8	<-->	e22	8.569	-.144
e8	<-->	e21	49.624	.384
e8	<-->	e16	8.363	-.242
e8	<-->	e15	8.300	-.214
e8	<-->	e14	8.708	-.235
e8	<-->	e13	29.195	.296
e8	<-->	e11	5.956	-.114
e8	<-->	e9	13.270	.180
e7	<-->	F6	5.477	-.187
e7	<-->	e27	7.417	-.136
e7	<-->	e26	10.390	.127
e7	<-->	e25	4.460	-.097
e7	<-->	e23	24.972	-.199
e7	<-->	e22	35.278	.241
e7	<-->	e21	18.446	-.193
e7	<-->	e14	17.448	.275
e7	<-->	e13	12.003	-.156
e7	<-->	e10	6.744	.093
e6	<-->	F7	4.187	.066
e6	<-->	e27	24.876	.257
e6	<-->	e24	11.742	-.167
e6	<-->	e23	41.423	.264
e6	<-->	e22	10.650	-.137
e6	<-->	e15	17.462	.264
e6	<-->	e11	5.266	.092
e5	<-->	F4	4.187	.049
e5	<-->	e24	32.043	.260
e5	<-->	e22	6.789	-.103
e5	<-->	e16	31.725	.378
e5	<-->	e13	7.189	-.118
e5	<-->	e8	15.908	-.222
e4	<-->	F4	4.001	.054
e4	<-->	e28	42.334	.483
e4	<-->	e24	24.297	.255
e4	<-->	e16	38.369	.468
e4	<-->	e12	18.086	.213
e3	<-->	F2	9.716	-.128
e3	<-->	e27	37.136	.300
e3	<-->	e26	7.993	-.110
e3	<-->	e25	5.447	-.106
e3	<-->	e23	48.211	.273
e3	<-->	e22	20.035	-.180
e3	<-->	e21	4.616	-.096
e3	<-->	e15	15.810	.240
e3	<-->	e11	22.217	.180
e3	<-->	e9	6.091	-.100
e3	<-->	e8	6.836	-.149
e2	<-->	F2	4.128	.076
e2	<-->	e27	4.428	-.094
e2	<-->	e26	17.091	.146
e2	<-->	e25	5.046	-.092
e2	<-->	e24	6.311	-.107
e2	<-->	e23	7.267	-.096
e2	<-->	e22	48.994	.255

			M.I.	Par Change
e2	<-->	e21	19.774	-.180
e2	<-->	e14	17.754	.249
e2	<-->	e13	14.836	-.156
e2	<-->	e10	10.940	.106
e2	<-->	e9	5.072	-.083
e2	<-->	e8	53.797	-.380
e2	<-->	e7	31.029	.237
e1	<-->	F4	5.072	-.056
e1	<-->	e28	7.820	-.190
e1	<-->	e26	7.367	-.107
e1	<-->	e25	24.826	.228
e1	<-->	e22	16.690	-.166
e1	<-->	e21	58.572	.344
e1	<-->	e16	10.150	-.220
e1	<-->	e15	4.590	-.131
e1	<-->	e14	6.674	-.170
e1	<-->	e13	34.202	.264
e1	<-->	e10	4.324	-.074
e1	<-->	e9	17.031	.168
e1	<-->	e8	121.347	.636
e1	<-->	e7	40.711	-.303
e1	<-->	e5	4.201	-.095
e1	<-->	e4	6.931	-.136

Variances: (Group number 1 - Default model)

	M.I.	Par Change

Regression Weights: (Group number 1 - Default model)

			M.I.	Par Change
SOLUNIV	<---	F4	4.419	.222
SOLUNIV	<---	CONFCUNI	4.564	-.079
SOLUNIV	<---	SOLBAR	73.951	.219
SOLUNIV	<---	SOLHGR	14.115	.139
SOLUNIV	<---	RELHOG	10.236	-.144
SOLUNIV	<---	ENTHG	7.747	-.111
SOLUNIV	<---	SOLREL	12.954	.103
SOLUNIV	<---	SOLHOB	44.656	.165
CONFCUNI	<---	F3	5.630	.122
CONFCUNI	<---	SOLUNIV	7.195	-.051
CONFCUNI	<---	CONFTRA	14.982	.076
CONFCUNI	<---	CONFBARR	89.117	.204
CONFCUNI	<---	RELIBAR	8.500	-.054
CONFCUNI	<---	CONFLHOG	64.667	.258
CONFCUNI	<---	CONFREL	18.477	.077
CONFCUNI	<---	CONFHOB	22.094	.080
RELUNIVE	<---	ENTUNI	5.410	.054
RELUNIVE	<---	SOLBAR	6.819	-.038
RELUNIVE	<---	ENTHG	6.569	-.059
RELUNIVE	<---	CONFHOB	6.165	-.033
ENTUNI	<---	SOLUNIV	4.816	-.038
ENTUNI	<---	RELUNIVE	5.692	.075
ENTUNI	<---	ENTTRAB	13.110	.052
ENTUNI	<---	SOLBAR	17.442	-.072
ENTUNI	<---	ENTTBA	36.338	.162
ENTUNI	<---	SOLHGR	5.147	-.057
ENTUNI	<---	ENTHG	7.389	.073
ENTUNI	<---	ENTREL	5.458	.032
SOLTRAB	<---	F3	4.655	.105
SOLTRAB	<---	SOLBAR	16.127	.071
SOLTRAB	<---	SOLHGR	22.544	.122
SOLTRAB	<---	SOLREL	54.469	.148
SOLTRAB	<---	SOLHOB	34.895	.101

			M.I.	Par Change
CONFTRA	<---	CONFCUNI	72.824	.186
CONFTRA	<---	CONFBARR	35.874	.103
CONFTRA	<---	CONFLHOG	27.242	.134
CONFTRA	<---	RELHOG	4.183	-.054
CONFTRA	<---	RELREL	5.089	-.028
CONFTRA	<---	CONFREL	29.140	.077
CONFTRA	<---	CONFHOB	32.875	.077
RELIOTRB	<---	F3	4.107	-.085
RELIOTRB	<---	CONFCUNI	22.073	-.104
RELIOTRB	<---	RELUNIVE	7.310	.075
RELIOTRB	<---	ENTUNI	5.553	-.056
RELIOTRB	<---	CONFBARR	9.603	-.055
RELIOTRB	<---	RELIBAR	4.367	.032
RELIOTRB	<---	CONFLHOG	17.390	-.109
RELIOTRB	<---	CONFREL	13.220	-.053
RELIOTRB	<---	SOLREL	8.127	-.049
RELIOTRB	<---	SOLHOB	4.477	-.031
RELIOTRB	<---	CONFHOB	14.009	-.052
ENTTRAB	<---	ENTUNI	31.852	.149
ENTTRAB	<---	ENTTBA	8.338	.076
ENTTRAB	<---	ENTHG	7.584	.073
ENTTRAB	<---	ENTREL	18.079	.058
ENTTRAB	<---	ENTHOB	13.155	.046
SOLBAR	<---	SOLUNIV	119.601	.286
SOLBAR	<---	RELUNIVE	5.066	-.107
SOLBAR	<---	ENTUNI	18.815	-.176
SOLBAR	<---	SOLTRAB	29.462	.143
SOLBAR	<---	RELIBAR	6.232	.064
SOLBAR	<---	ENTTBA	14.946	-.157
SOLBAR	<---	SOLHGR	29.043	.203
SOLBAR	<---	RELHOG	13.221	-.167
SOLBAR	<---	ENTHG	5.175	-.092
SOLBAR	<---	SOLREL	70.114	.245
SOLBAR	<---	SOLHOB	56.369	.188
CONFBARR	<---	F7	8.889	.297
CONFBARR	<---	F2	7.541	-.115
CONFBARR	<---	CONFCUNI	106.803	.346
CONFBARR	<---	CONFTRA	7.945	.068
CONFBARR	<---	CONFLHOG	19.772	.175
CONFBARR	<---	RELHOG	5.132	-.092
CONFBARR	<---	ENTREL	13.280	-.068
CONFBARR	<---	RELREL	8.612	-.057
CONFBARR	<---	SOLREL	4.374	-.054
CONFBARR	<---	CONFHOB	6.352	.052
RELIBAR	<---	F7	8.532	-.310
RELIBAR	<---	SOLUNIV	4.836	-.055
RELIBAR	<---	CONFCUNI	29.067	-.194
RELIBAR	<---	ENTUNI	5.121	-.087
RELIBAR	<---	SOLBAR	9.787	.077
RELIBAR	<---	SOLHGR	4.383	-.075
RELIBAR	<---	CONFLHOG	10.934	-.140
RELIBAR	<---	RELREL	6.495	.053
ENTTBA	<---	F7	7.378	.200
ENTTBA	<---	F3	9.182	.141
ENTTBA	<---	SOLUNIV	8.604	-.050
ENTTBA	<---	ENTUNI	48.777	.185
ENTTBA	<---	SOLTRAB	5.146	-.039
ENTTBA	<---	SOLBAR	13.235	-.061
ENTTBA	<---	ENTHG	52.212	.191
ENTTBA	<---	RELREL	5.268	-.033
ENTTBA	<---	SOLREL	11.044	-.063
ENTTBA	<---	ENTHOB	6.657	.033
SOLHGR	<---	SOLUNIV	58.212	.132
SOLHGR	<---	SOLTRAB	31.523	.098

			M.I.	Par Change
SOLHGR	<---	SOLBAR	41.044	.110
SOLHGR	<---	ENTTBA	4.137	-.055
SOLHGR	<---	ENTHG	6.488	-.068
SOLHGR	<---	SOLREL	14.648	.074
SOLHGR	<---	SOLHOB	25.463	.084
CONFLHOG	<---	F7	10.514	.205
CONFLHOG	<---	F2	4.365	-.055
CONFLHOG	<---	CONFCUNI	79.385	.189
CONFLHOG	<---	CONFTRA	6.891	.040
CONFLHOG	<---	CONFBARR	31.708	.094
CONFLHOG	<---	ENTREL	8.401	-.034
CONFLHOG	<---	RELREL	5.332	-.028
CONFLHOG	<---	CONFHOB	10.742	.043
RELHOG	<---	F7	10.446	-.189
RELHOG	<---	F4	8.635	-.163
RELHOG	<---	SOLUNIV	21.412	-.063
RELHOG	<---	CONFCUNI	5.786	-.047
RELHOG	<---	ENTUNI	5.940	-.051
RELHOG	<---	SOLBAR	27.824	-.071
RELHOG	<---	CONFBARR	14.200	-.058
RELHOG	<---	CONFHOB	4.869	-.027
ENTHG	<---	SOLUNIV	10.775	-.051
ENTHG	<---	RELUNIVE	7.800	-.078
ENTHG	<---	ENTUNI	11.997	.083
ENTHG	<---	ENTTBA	56.191	.180
ENTHG	<---	SOLHGR	8.398	-.064
ENTHG	<---	ENTREL	5.866	.030
ENTREL	<---	F6	13.831	.078
ENTREL	<---	F4	4.742	-.196
ENTREL	<---	RELUNIVE	6.937	-.105
ENTREL	<---	ENTUNI	8.775	.100
ENTREL	<---	SOLTRAB	4.480	.047
ENTREL	<---	CONFTRA	7.176	.061
ENTREL	<---	RELIOTRB	7.725	.049
ENTREL	<---	ENTTRAB	35.881	.109
ENTREL	<---	SOLBAR	10.643	-.071
ENTREL	<---	CONFBARR	10.770	-.082
ENTREL	<---	RELIBAR	9.623	-.067
ENTREL	<---	ENTTBA	14.362	.129
ENTREL	<---	CONFLHOG	4.649	-.080
ENTREL	<---	ENTHG	4.433	.071
ENTREL	<---	SOLREL	13.384	-.090
ENTREL	<---	ENTHOB	21.741	.076
RELREL	<---	F6	5.651	-.041
RELREL	<---	CONFCUNI	7.558	-.072
RELREL	<---	RELUNIVE	5.390	.076
RELREL	<---	ENTUNI	4.667	-.060
RELREL	<---	CONFTRA	18.548	-.081
RELREL	<---	ENTTRAB	14.047	-.056
RELREL	<---	RELIBAR	5.930	.043
RELREL	<---	ENTTBA	9.746	-.087
RELREL	<---	ENTHOB	8.622	-.040
CONFREL	<---	CONFCUNI	23.922	.132
CONFREL	<---	SOLTRAB	8.013	-.053
CONFREL	<---	CONFTRA	4.898	.043
CONFREL	<---	CONFBARR	15.565	.084
SOLREL	<---	F4	7.001	.191
SOLREL	<---	F3	4.105	.097
SOLREL	<---	SOLUNIV	9.670	.055
SOLREL	<---	SOLTRAB	17.767	.075
SOLREL	<---	SOLBAR	32.097	.098
SOLREL	<---	SOLHGR	5.201	.058
SOLREL	<---	CONFLHOG	4.926	.066
SOLREL	<---	ENTREL	4.157	-.029

			M.I.	Par Change
SOLREL	<---	CONFHOB	6.660	.041
SOLHOB	<---	F7	10.394	.275
SOLHOB	<---	F4	10.894	.268
SOLHOB	<---	F3	7.184	.144
SOLHOB	<---	SOLUNIV	81.671	.179
SOLHOB	<---	SOLTRAB	30.131	.109
SOLHOB	<---	SOLBAR	40.949	.125
SOLHOB	<---	SOLHGR	20.958	.130
CONFHOB	<---	F2	9.669	-.100
CONFHOB	<---	CONFCUNI	30.881	.144
CONFHOB	<---	RELUNIVE	4.678	-.070
CONFHOB	<---	RELIOTRB	7.412	-.039
CONFHOB	<---	ENTTRAB	6.044	-.037
CONFHOB	<---	CONFBARR	11.742	.070
CONFHOB	<---	CONFLHOG	11.385	.103
CONFHOB	<---	ENTREL	14.464	-.054
CONFHOB	<---	RELREL	8.359	-.043
CONFHOB	<---	CONFREL	5.336	-.039
RELHOB	<---	RELUNIVE	12.263	.103
RELHOB	<---	RELIBAR	9.650	.050
RELHOB	<---	ENTTBA	9.074	-.076
RELHOB	<---	RELHOG	5.439	.066
RELHOB	<---	ENTREL	4.589	-.028
RELHOB	<---	RELREL	10.361	.043
RELHOB	<---	SOLREL	9.162	.055
ENTHOB	<---	F7	4.851	-.172
ENTHOB	<---	F4	6.664	-.191
ENTHOB	<---	SOLUNIV	13.425	-.066
ENTHOB	<---	CONFCUNI	4.867	-.058
ENTHOB	<---	RELUNIVE	9.213	-.099
ENTHOB	<---	ENTUNI	10.961	.092
ENTHOB	<---	ENTTRAB	13.220	.055
ENTHOB	<---	SOLBAR	13.490	-.066
ENTHOB	<---	CONFBARR	7.840	-.058
ENTHOB	<---	RELIBAR	9.556	-.055
ENTHOB	<---	ENTTBA	16.137	.112
ENTHOB	<---	CONFLHOG	4.051	-.062
ENTHOB	<---	RELHOG	4.753	-.069
ENTHOB	<---	ENTREL	28.741	.078
ENTHOB	<---	SOLREL	9.505	-.062
ENTHOB	<---	SOLHOB	9.528	-.053

Minimization History (Default model)

Iteration		Negative eigenvalues	Condition #	Smallest eigenvalue	Diameter	F	NTries	Ratio
0	e	16		-.637	9999.000	15602.017	0	9999.000
1	e*	15		-.537	3.405	7600.606	20	.444
2	e	4		-.159	.693	4995.499	6	.949
3	e	2		-.005	.490	4117.979	5	.632
4	e*	0	2043.800		.652	3042.771	5	.833
5	e	0	456.507		1.064	2628.247	3	.000
6	e	0	1301.226		1.091	2248.852	1	.916
7	e	0	2906.134		.683	2229.119	2	.000
8	e	1		-.004	.880	2178.658	2	.000
9	e	0	954.913		.893	2135.584	12	.797
10	e	0	666.508		.190	2128.153	1	1.075
11	e	0	709.599		.098	2127.498	1	1.028
12	e	0	712.529		.007	2127.483	1	1.004
13	e	0	722.380		.000	2127.483	1	1.000

Model Fit Summary

CMIN

Model	NPAR	CMIN	*df*	*p*	CMIN/DF
Default model	66	2127.483	234	.000	9.092
Saturated model	300	.000	0		

Model	NPAR	CMIN	*df*	*p*	CMIN/DF
Independence model	24	15261.917	276	.000	55.297

RMR. GFI

Model	RMR	GFI	AGFI	PGFI
Default model	.209	.852	.810	.665
Saturated model	.000	1.000		
Independence model	1.132	.434	.385	.399

Baseline Comparisons

Model	NFI Delta1	RFI rho1	IFI Delta2	TLI rho2	CFI
Default model	.861	.836	.874	.851	.874
Saturated model	1.000		1.000		1.000
Independence model	.000	.000	.000	.000	.000

Parsimony-Adjusted Measures

Model	PRATIO	PNFI	PCFI
Default model	.848	.730	.741
Saturated model	.000	.000	.000
Independence model	1.000	.000	.000

NCP

Model	NCP	LO 90	HI 90
Default model	1893.483	1749.634	2044.740
Saturated model	.000	.000	.000
Independence model	14985.917	14584.516	15393.634

FMIN

Model	FMIN	F0	LO 90	HI 90
Default model	1.728	1.538	1.421	1.661
Saturated model	.000	.000	.000	.000
Independence model	12.398	12.174	11.848	12.505

RMSEA

Model	RMSEA	LO 90	HI 90	PCLOSE
Default model	.081	.078	.084	.000
Independence model	.210	.207	.213	.000

AIC

Model	AIC	BCC	BIC	CAIC
Default model	2259.483	2262.220	2597.165	2663.165
Saturated model	600.000	612.438	2134.918	2434.918
Independence model	15309.917	15310.912	15432.711	15456.711

ECVI

Model	ECVI	LO 90	HI 90	MECVI
Default model	1.835	1.719	1.958	1.838
Saturated model	.487	.487	.487	.498
Independence model	12.437	12.111	12.768	12.438

HOELTER

Model	HOELTER .05	HOELTER .01
Default model	157	167
Independence model	26	27

Execution time summary

Minimization:	.017
Miscellaneous:	.564
Bootstrap:	.000
Total:	.581

Path Diagram: Confirmatory Factor Analysis

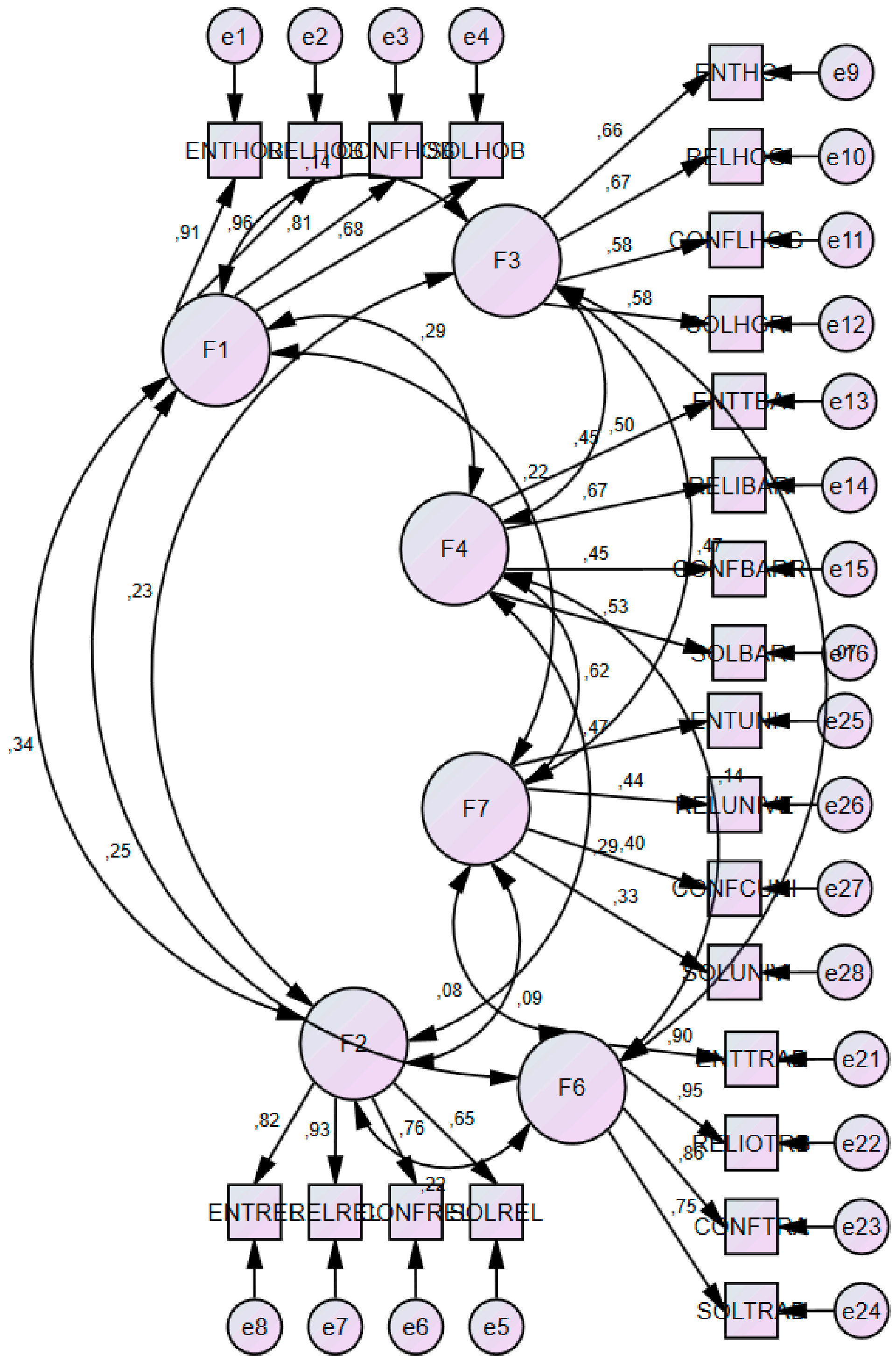

CONFIRMATORY FACTOR ANALYSIS OF COMPLETE PROTECTIVE FACTORS

C:\Users\media\Dropbox\Mi PC (LAPTOP-LCRG33R5)\Desktop\2024\Articulos 2024\Salud mental

2024 1\Resultados junio final\factores de proteccion.amw

Analysis Summary

Date and Time

Date: sábado, 22 de junio de 2024
Time: 6:36:42 a. m.

Title

factores de proteccion: sábado, 22 de junio de 2024 6:36 a. m.

Groups

Group number 1 (Group number 1)

Notes for Group (Group number 1)

The model is recursive.
Sample size = 1232

Variable Summary (Group number 1)

Your model contains the following variables (Group number 1)

Observed, endogenous variables
AMIGOS
COMFAM
GRUPOS
RECREA
CULTUR
DEPORT
DORMIR
COMER
APOYPAR
APOYFAM
APOYAMIG
APOYCOMP
APOTTRAB
APOYDOC
APOYBIE
APOYCOM
FORTALZ
EMPATI
EXPRSN
INTERACT
DECISNS
AFRONT
INNOVA
CUESTIN
EXPRESEMOC
INDENTIF

Variable counts (Group number 1)

Number of variables in your model:	55
Number of observed variables:	26
Number of unobserved variables:	29
Number of exogenous variables:	29
Number of endogenous variables:	26

Parameter Summary (Group number 1)

	Weights	Covariances	Variances	Means	Intercepts	Total
Fixed	29	0	0	29	0	58
Labeled	0	0	0	0	0	0
Unlabeled	23	9	29	0	26	87
Total	52	9	29	29	26	145

Models

Default model (Default model)

Notes for Model (Default model)

Computation of degrees of freedom (Default model)

Number of distinct sample moments:	377
Number of distinct parameters to be estimated:	87
Degrees of freedom (377 - 87):	290

Result (Default model)

Minimum was achieved
Chi-square = 1173,185
Degrees of freedom = 290
Probability level = ,000

Group number 1 (Group number 1 - Default model)

Estimates (Group number 1 - Default model)

Scalar Estimates (Group number 1 - Default model)

Maximum Likelihood Estimates

Regression Weights: (Group number 1 - Default model)

			Estimate	S.E.	C.R.	*p*	Label
AMIGOS	<---	F1	1.000				
COMFAM	<---	F1	.839	.066	12.681	***	
GRUPOS	<---	F1	.975	.086	11.291	***	
RECREA	<---	F1	1.700	.120	14.143	***	
CULTUR	<---	F1	1.365	.097	14.074	***	
DEPORT	<---	F1	1.111	.103	10.754	***	
DORMIR	<---	F1	.266	.065	4.078	***	
COMER	<---	F1	.424	.072	5.920	***	
APOYPAR	<---	F2	1.000				
APOYFAM	<---	F2	2.266	1.385	1.636	.102	
APOYAMIG	<---	F2	3.306	2.017	1.639	.101	
APOYCOMP	<---	F2	6.674	4.024	1.658	.097	
APOTTRAB	<---	F2	4.677	2.845	1.644	.100	
APOYDOC	<---	F2	11.274	6.779	1.663	.096	
APOYBIE	<---	F2	10.794	6.492	1.663	.096	
APOYCOM	<---	F2	9.264	5.574	1.662	.097	
FORTALZ	<---	F3	1.000				
EMPATI	<---	F3	.678	.045	15.161	***	
EXPRSN	<---	F3	1.096	.057	19.240	***	
INTERACT	<---	F3	1.029	.052	19.719	***	
DECISNS	<---	F3	1.089	.051	21.188	***	
AFRONT	<---	F3	1.279	.058	22.232	***	
INNOVA	<---	F3	1.307	.058	22.421	***	
CUESTIN	<---	F3	1.105	.055	20.143	***	
EXPRESEMOC	<---	F3	1.118	.058	19.199	***	
INDENTIF	<---	F3	1.151	.058	20.020	***	

Standardized Regression Weights: (Group number 1 - Default model)

			Estimate
AMIGOS	<---	F1	.431
COMFAM	<---	F1	.372
GRUPOS	<---	F1	.466
RECREA	<---	F1	.859
CULTUR	<---	F1	.787
DEPORT	<---	F1	.429
DORMIR	<---	F1	.130

			Estimate
COMER	<---	F1	.196
APOYPAR	<---	F2	.052
APOYFAM	<---	F2	.268
APOYAMIG	<---	F2	.283
APOYCOMP	<---	F2	.518
APOTTRAB	<---	F2	.311
APOYDOC	<---	F2	.817
APOYBIE	<---	F2	.740
APOYCOM	<---	F2	.681
FORTALZ	<---	F3	.627
EMPATI	<---	F3	.487
EXPRSN	<---	F3	.647
INTERACT	<---	F3	.667
DECISNS	<---	F3	.732
AFRONT	<---	F3	.782
INNOVA	<---	F3	.792
CUESTIN	<---	F3	.685
EXPRESEMOC	<---	F3	.648
INDENTIF	<---	F3	.681

Intercepts: (Group number 1 - Default model)

	Estimate	S.E.	C.R.	*p*	Label
AMIGOS	3.645	.040	91.369	***	
COMFAM	3.326	.039	85.886	***	
GRUPOS	1.996	.036	55.478	***	
RECREA	2.380	.034	69.896	***	
CULTUR	2.127	.030	71.279	***	
DEPORT	3.585	.045	80.436	***	
DORMIR	4.133	.035	117.636	***	
COMER	4.226	.037	113.870	***	
APOYPAR	2.549	.081	31.518	***	
APOYFAM	5.359	.036	150.084	***	
APOYAMIG	4.632	.049	93.804	***	
APOYCOMP	3.704	.054	68.093	***	
APOTTRAB	1.527	.064	24.028	***	
APOYDOC	2.994	.058	51.400	***	
APOYBIE	3.180	.062	51.693	***	
APOYCOM	2.154	.057	37.508	***	
FORTALZ	3.741	.031	120.475	***	
EMPATI	4.015	.027	148.012	***	
EXPRSN	3.379	.033	102.507	***	
INTERACT	3.791	.030	126.120	***	
DECISNS	3.773	.029	130.354	***	
AFRONT	3.537	.032	111.069	***	
INNOVA	3.427	.032	106.598	***	
CUESTIN	3.761	.031	119.767	***	
EXPRESEMOC	3.381	.034	100.564	***	
INDENTIF	3.473	.033	105.455	***	

Covariances: (Group number 1 - Default model)

			Estimate	S.E.	C.R.	*p*	Label
F1	<-->	F2	.019	.012	1.596	.110	
F3	<-->	F1	.107	.016	6.690	***	
F3	<-->	F2	.018	.011	1.584	.113	
e1	<-->	e2	.642	.051	12.506	***	
e7	<-->	e8	.508	.046	11.090	***	
e11	<-->	e12	.993	.087	11.376	***	
e6	<-->	e8	.405	.051	7.895	***	
e19	<-->	e25	.202	.025	8.226	***	
e25	<-->	e26	.193	.024	8.073	***	

Correlations: (Group number 1 - Default model)

			Estimate
F1	<-->	F2	.210
F3	<-->	F1	.259
F3	<-->	F2	.175
e1	<-->	e2	.403
e7	<-->	e8	.326
e11	<-->	e12	.366
e6	<-->	e8	.225
e19	<-->	e25	.255
e25	<-->	e26	.254

Variances: (Group number 1 - Default model)

	Estimate	S.E.	C.R.	*p*	Label
F1	.364	.050	7.285	***	
F2	.022	.026	.832	.405	
F3	.467	.040	11.724	***	
e1	1.595	.067	23.705	***	
e2	1.590	.066	24.026	***	
e3	1.247	.053	23.473	***	
e4	.375	.037	10.183	***	
e5	.418	.028	15.054	***	
e6	1.996	.084	23.731	***	
e7	1.494	.060	24.728	***	
e8	1.630	.066	24.766	***	
e9	8.028	.324	24.795	***	
e10	1.457	.060	24.399	***	
e11	2.762	.114	24.318	***	
e12	2.665	.117	22.824	***	
e13	4.490	.185	24.242	***	
e14	1.389	.103	13.541	***	
e15	2.104	.119	17.756	***	
e16	2.179	.109	19.949	***	
e17	.720	.031	22.992	***	
e18	.691	.029	23.941	***	
e19	.777	.034	22.744	***	
e20	.617	.027	22.559	***	
e21	.478	.022	21.549	***	
e22	.485	.024	20.357	***	
e23	.475	.024	20.065	***	
e24	.644	.029	22.319	***	
e25	.808	.035	22.770	***	
e26	.717	.032	22.332	***	

Matrices (Group number 1 - Default model)

Total Effects (Group number 1 - Default model)

	F2	F1	F3
INDENTIF	.000	.000	1.151
EXPRESEMOC	.000	.000	1.118
CUESTIN	.000	.000	1.105
INNOVA	.000	.000	1.307
AFRONT	.000	.000	1.279
DECISNS	.000	.000	1.089
INTERACT	.000	.000	1.029
EXPRSN	.000	.000	1.096
EMPATI	.000	.000	.678
FORTALZ	.000	.000	1.000
APOYCOM	9.264	.000	.000
APOYBIE	10.794	.000	.000
APOYDOC	11.274	.000	.000
APOTTRAB	4.677	.000	.000
APOYCOMP	6.674	.000	.000
APOYAMIG	3.306	.000	.000

	F2	F1	F3
APOYFAM	2.266	.000	.000
APOYPAR	1.000	.000	.000
COMER	.000	.424	.000
DORMIR	.000	.266	.000
DEPORT	.000	1.111	.000
CULTUR	.000	1.365	.000
RECREA	.000	1.700	.000
GRUPOS	.000	.975	.000
COMFAM	.000	.839	.000
AMIGOS	.000	1.000	.000

Standardized Total Effects (Group number 1 - Default model)

	F2	F1	F3
INDENTIF	.000	.000	.681
EXPRESEMOC	.000	.000	.648
CUESTIN	.000	.000	.685
INNOVA	.000	.000	.792
AFRONT	.000	.000	.782
DECISNS	.000	.000	.732
INTERACT	.000	.000	.667
EXPRSN	.000	.000	.647
EMPATI	.000	.000	.487
FORTALZ	.000	.000	.627
APOYCOM	.681	.000	.000
APOYBIE	.740	.000	.000
APOYDOC	.817	.000	.000
APOTTRAB	.311	.000	.000
APOYCOMP	.518	.000	.000
APOYAMIG	.283	.000	.000
APOYFAM	.268	.000	.000
APOYPAR	.052	.000	.000
COMER	.000	.196	.000
DORMIR	.000	.130	.000
DEPORT	.000	.429	.000
CULTUR	.000	.787	.000
RECREA	.000	.859	.000
GRUPOS	.000	.466	.000
COMFAM	.000	.372	.000
AMIGOS	.000	.431	.000

Direct Effects (Group number 1 - Default model)

	F2	F1	F3
INDENTIF	.000	.000	1.151
EXPRESEMOC	.000	.000	1.118
CUESTIN	.000	.000	1.105
INNOVA	.000	.000	1.307
AFRONT	.000	.000	1.279
DECISNS	.000	.000	1.089
INTERACT	.000	.000	1.029
EXPRSN	.000	.000	1.096
EMPATI	.000	.000	.678
FORTALZ	.000	.000	1.000
APOYCOM	9.264	.000	.000
APOYBIE	10.794	.000	.000
APOYDOC	11.274	.000	.000
APOTTRAB	4.677	.000	.000
APOYCOMP	6.674	.000	.000
APOYAMIG	3.306	.000	.000
APOYFAM	2.266	.000	.000
APOYPAR	1.000	.000	.000
COMER	.000	.424	.000
DORMIR	.000	.266	.000

	F2	F1	F3
DEPORT	.000	1.111	.000
CULTUR	.000	1.365	.000
RECREA	.000	1.700	.000
GRUPOS	.000	.975	.000
COMFAM	.000	.839	.000
AMIGOS	.000	1.000	.000

Standardized Direct Effects (Group number 1 - Default model)

	F2	F1	F3
INDENTIF	.000	.000	.681
EXPRESEMOC	.000	.000	.648
CUESTIN	.000	.000	.685
INNOVA	.000	.000	.792
AFRONT	.000	.000	.782
DECISNS	.000	.000	.732
INTERACT	.000	.000	.667
EXPRSN	.000	.000	.647
EMPATI	.000	.000	.487
FORTALZ	.000	.000	.627
APOYCOM	.681	.000	.000
APOYBIE	.740	.000	.000
APOYDOC	.817	.000	.000
APOTTRAB	.311	.000	.000
APOYCOMP	.518	.000	.000
APOYAMIG	.283	.000	.000
APOYFAM	.268	.000	.000
APOYPAR	.052	.000	.000
COMER	.000	.196	.000
DORMIR	.000	.130	.000
DEPORT	.000	.429	.000
CULTUR	.000	.787	.000
RECREA	.000	.859	.000
GRUPOS	.000	.466	.000
COMFAM	.000	.372	.000
AMIGOS	.000	.431	.000

Modification Indices (Group number 1 - Default model)

Covariances: (Group number 1 - Default model)

			M.I.	Par Change
e24	<-->	e25	10.578	.066
e23	<-->	e24	34.254	.105
e22	<-->	e24	7.726	-.050
e22	<-->	e23	37.555	.098
e21	<-->	F1	12.842	-.048
e20	<-->	e24	5.078	-.044
e20	<-->	e23	32.423	-.100
e20	<-->	e22	10.281	-.056
e20	<-->	e21	15.045	.066
e19	<-->	F1	4.537	.034
e19	<-->	e24	21.300	-.096
e19	<-->	e22	6.567	-.048
e19	<-->	e20	26.791	.105
e18	<-->	e26	11.412	-.069
e18	<-->	e24	12.311	.071
e18	<-->	e23	13.006	-.065
e18	<-->	e22	5.588	-.043
e18	<-->	e20	4.198	.040
e18	<-->	e19	4.563	.045
e17	<-->	e26	7.973	.059
e17	<-->	e25	9.527	-.065
e17	<-->	e24	9.358	-.064

			M.I.	Par Change
e17	<-->	e19	12.590	.077
e17	<-->	e18	4.627	.045
e16	<-->	F1	10.978	.096
e15	<-->	F3	5.992	-.080
e15	<-->	e26	4.710	.085
e15	<-->	e16	4.993	.163
e14	<-->	F1	6.092	-.065
e14	<-->	F3	4.844	-.064
e14	<-->	e16	7.161	-.171
e14	<-->	e15	7.681	.177
e13	<-->	F3	4.101	.086
e13	<-->	e15	20.226	-.447
e12	<-->	e19	6.160	.096
e12	<-->	e15	16.099	-.289
e12	<-->	e13	14.796	.365
e11	<-->	F3	13.548	.113
e11	<-->	e24	5.436	.086
e11	<-->	e18	4.056	.075
e10	<-->	F3	35.169	.143
e10	<-->	e17	11.381	.103
e10	<-->	e11	45.066	.360
e9	<-->	F3	15.696	.223
e9	<-->	e19	5.278	.162
e9	<-->	e17	7.172	.190
e9	<-->	e15	7.154	-.353
e9	<-->	e13	20.771	.787
e9	<-->	e10	5.724	.235
e8	<-->	F3	30.765	.129
e8	<-->	e25	4.739	.062
e8	<-->	e18	4.014	-.057
e8	<-->	e17	5.737	.070
e8	<-->	e15	4.912	-.121
e8	<-->	e10	4.115	.082
e7	<-->	F2	7.663	.015
e7	<-->	F3	24.481	.113
e7	<-->	e26	10.961	.092
e7	<-->	e15	4.989	.120
e7	<-->	e13	8.523	-.205
e7	<-->	e10	8.965	.120
e6	<-->	F3	11.821	.095
e6	<-->	e20	4.632	.070
e6	<-->	e7	9.978	.144
e5	<-->	F3	6.296	-.037
e5	<-->	e21	5.534	-.037
e5	<-->	e19	4.023	.037
e5	<-->	e10	6.476	-.066
e4	<-->	F3	7.195	-.044
e4	<-->	e7	6.198	-.067
e3	<-->	e19	5.860	-.069
e3	<-->	e6	17.119	-.188
e2	<-->	F2	9.779	.016
e2	<-->	F3	12.441	.081
e2	<-->	e25	7.701	.078
e2	<-->	e17	4.790	.063
e2	<-->	e10	38.855	.250
e2	<-->	e8	14.066	.145
e2	<-->	e7	4.661	.082
e2	<-->	e4	7.602	-.075
e2	<-->	e3	18.100	.160
e1	<-->	F3	5.020	.052
e1	<-->	e20	7.124	.073
e1	<-->	e14	4.508	-.103
e1	<-->	e11	85.836	.477
e1	<-->	e3	5.169	-.086

Variances: (Group number 1 - Default model)

	M.I.	Par Change

Regression Weights: (Group number 1 - Default model)

			M.I.	Par Change
DECISNS	<---	F1	12.303	-.133
APOYCOM	<---	F1	11.838	.284
APOYBIE	<---	F3	7.654	-.199
APOYBIE	<---	APOTTRAB	12.270	-.060
APOYDOC	<---	F1	8.827	-.224
APOYDOC	<---	F3	7.602	-.178
APOTTRAB	<---	F3	4.756	.205
APOTTRAB	<---	APOYPAR	11.458	.054
APOYCOMP	<---	APOTTRAB	8.939	.049
APOYAMIG	<---	F1	5.749	.191
APOYAMIG	<---	F3	16.230	.274
APOYAMIG	<---	AMIGOS	11.098	.038
APOYFAM	<---	F3	36.271	.322
APOYFAM	<---	APOYAMIG	5.958	.017
APOYFAM	<---	COMFAM	6.240	.024
APOYPAR	<---	F3	15.909	.497
APOYPAR	<---	APOTTRAB	12.535	.106
COMER	<---	F3	28.035	.273
DORMIR	<---	F2	11.193	.818
DORMIR	<---	F3	25.939	.258
DORMIR	<---	APOYBIE	4.045	.017
DEPORT	<---	F3	10.793	.202
CULTUR	<---	F3	5.553	-.078
RECREA	<---	F2	4.767	-.380
RECREA	<---	F3	7.921	-.102
COMFAM	<---	F2	12.331	.863
COMFAM	<---	F3	14.188	.192
AMIGOS	<---	F3	4.555	.110
AMIGOS	<---	APOYAMIG	11.440	.023

Means: (Group number 1 - Default model)

	M.I.	Par Change

Intercepts: (Group number 1 - Default model)

	M.I.	Par Change

Minimization History (Default model)

Iteration		Negative eigenvalues	Condition #	Smallest eigenvalue	Diameter	F	NTries	Ratio
0	e	7		-1.112	9999.000	10205.824	0	9999.000
1	e	5		-.373	3.027	5747.136	20	.226
2	e	3		-.162	1.068	3967.114	4	.740
3	e	0	2127.086		1.387	2321.807	5	.857
4	e	0	281.774		.777	1981.981	5	.000
5	e	0	353.879		.757	1611.956	2	.000
6	e	0	558.708		.972	1396.100	1	1.157
7	e	0	1395.377		.818	1288.494	1	1.245
8	e	0	5095.751		1.188	1265.738	1	.550
9	e	0	20725.096		.779	1215.353	1	1.134
10	e	0	35501.202		.733	1205.412	2	.000
11	e	0	101280.197		1.076	1195.078	1	1.281
12	e	0	289618.067		1.047	1188.117	1	1.324
13	e	0	621702.977		1.236	1183.871	1	1.207
14	e	0	2171624.597		.982	1180.519	1	1.319
15	e	0	2334627.916		1.643	1179.595	1	.490
16	e	0	16554687.848		.743	1176.826	1	1.118
17	e	0	9261075.811		1.448	1176.275	2	.000
18	e	0	54719332.938		.774	1175.133	1	1.173
19	e	0	29423271.068		1.365	1174.781	2	.000
20	e	0	145698709.922		.801	1174.227	1	1.223
21	e	0	88158074.547		1.193	1174.006	2	.000
22	e	0	281606559.676		.884	1173.735	1	1.305

Iteration		Negative eigenvalues	Condition #	Smallest eigenvalue	Diameter	F	NTries	Ratio
23	e	0	147396838.741		1.759	1173.723	1	.069
24	e	0	1394732273.697		.527	1173.422	1	1.069
25	e	0	375981712.866		1.494	1173.401	2	.000
26	e	0	2883286610.636		.484	1173.281	1	1.090
27	e	0	1011599927.501		1.187	1173.258	2	.000
28	e	0	4896802234.258		.474	1173.219	1	1.153
29	e	0	2763900561.097		.773	1173.206	2	.000
30	e	0	5818695971.522		.544	1173.194	1	1.294
31	e	0	4968764149.496		.713	1173.189	1	1.041
32	e	0	12932457056.181		.275	1173.186	1	1.169
33	e	0	11289167879.558		.394	1173.186	1	.972
34	e	0	17729513488.474		.067	1173.185	1	1.042
35	e	0	18284009241.744		.034	1173.185	1	1.011
36	e	0	17899359760.944		.000	1173.185	1	1.000

Model Fit Summary

CMIN

Model	NPAR	CMIN	*df*	*p*	CMIN/DF
Default model	87	1173.185	290	.000	4.045
Saturated model	377	.000	0		
Independence model	52	10470.276	325	.000	32.216

Baseline Comparisons

Model	NFI Delta1	RFI rho1	IFI Delta2	TLI rho2	CFI
Default model	.888	.874	.913	.902	.913
Saturated model	1.000		1.000		1.000
Independence model	.000	.000	.000	.000	.000

Parsimony-Adjusted Measures

Model	PRATIO	PNFI	PCFI
Default model	.892	.792	.815
Saturated model	.000	.000	.000
Independence model	1.000	.000	.000

NCP

Model	NCP	LO 90	HI 90
Default model	883.185	781.434	992.478
Saturated model	.000	.000	.000
Independence model	10145.276	9814.462	10482.434

FMIN

Model	FMIN	F0	LO 90	HI 90
Default model	.953	.717	.635	.806
Saturated model	.000	.000	.000	.000
Independence model	8.506	8.241	7.973	8.515

RMSEA

Model	RMSEA	LO 90	HI 90	PCLOSE
Default model	.050	.047	.053	.552
Independence model	.159	.157	.162	.000

AIC

Model	AIC	BCC	BIC	CAIC
Default model	1347.185	1351.087	1792.311	1879.311
Saturated model	754.000	770.909	2682.880	3059.880
Independence model	10574.276	10576.608	10840.328	10892.328

ECVI

Model	ECVI	LO 90	HI 90	MECVI
Default model	1.094	1.012	1.183	1.098
Saturated model	.613	.613	.613	.626
Independence model	8.590	8.321	8.864	8.592

HOELTER

Model	HOELTER .05	HOELTER .01
Default model	348	367
Independence model	44	46

Execution time summary

Minimization:	.048
Miscellaneous:	.718
Bootstrap:	.000
Total:	.766

Path Diagram: Confirmatory Factor Analysis

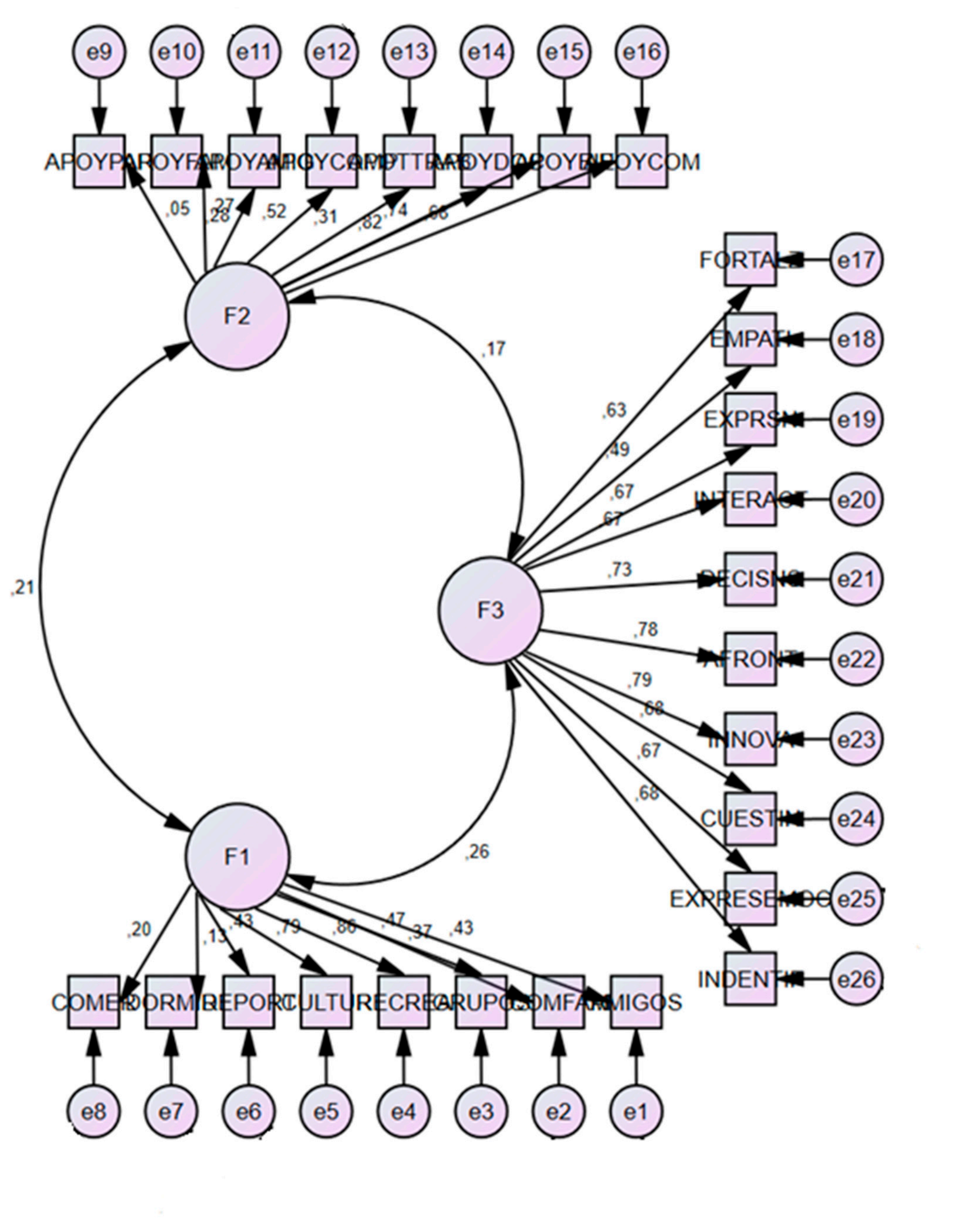

CONFIRMATORY FACTOR ANALYSIS RISK FACTORS

Users\media\Dropbox\Mi PC (LAPTOP-LCRG33R5)\Desktop\2024\Articulos 2024\Salud mental 2024 1\Resultados junio final\Factores de riesgo.amw

Analysis Summary

Date and Time

Date: sábado, 22 de junio de 2024
Time: 9:56:42 a. m.

Title

Factores de riesgo: sábado, 22 de junio de 2024 9:56 a. m.

Groups

Group number 1 (Group number 1)

Notes for Group (Group number 1)

The model is recursive.
Sample size = 1232

Variable Summary (Group number 1)

Your model contains the following variables (Group number 1)

Observed, endogenous variables
IDEACI
INTENT
DAÑO
COMNERGZ
COMMDIC
CONSDRG
CONTABAC
CONSALCH
MALPAR
MALFAM
MALSOCL
MALFINAZ
MALESSLD
VIDEOJG
PORN
APUESTAS
RESDSLS
CFFAMILIA
CFPAREJA
CFAMIGS
CFECNM
CFLABR
CFACADM
CFLEGAL
PROLGLS
PROBACDM
PRMLABR
PROMECON
CONFAMIG
CONFPRJ
CONFFAM
Unobserved, exogenous variables
SUIC
e1
e2
e3
DRUG
e4
e5
e6
e7
e8
MALEST
e9
e10
e11
e12
e13
INTERN

e14
e15
e16
e17
CONFADD
e18
e19
e20
e21
e22
e23
e24
CONFDRUG
e25
e26
e27
e28
e29
e30
e31

Variable counts (Group number 1)

Number of variables in your model: 68
Number of observed variables: 31
Number of unobserved variables: 37
Number of exogenous variables: 37
Number of endogenous variables: 31

Parameter Summary (Group number 1)

	Weights	Covariances	Variances	Means	Intercepts	Total
Fixed	37	0	0	0	0	37
Labeled	0	0	0	0	0	0
Unlabeled	25	19	37	0	0	81
Total	62	19	37	0	0	118

Models

Default model (Default model)

Notes for Model (Default model)

Computation of degrees of freedom (Default model)

Number of distinct sample moments: 496
Number of distinct parameters to be estimated: 81
Degrees of freedom (496 - 81): 415

Result (Default model)

Minimum was achieved
Chi-square = 1721. 005
Degrees of freedom =0 415
Probability level = 0.000

Group number 1 (Group number 1 - Default model)

Estimates (Group number 1 - Default model)

Scalar Estimates (Group number 1 - Default model)

Maximum Likelihood Estimates

Regression Weights: (Group number 1 — Default model)

			Estimate	S.E.	C.R.	*p*	Label
IDEACI	<———	SUIC	1.000				
INTENT	<———	SUIC	.746	.064	11.728	***	

			Estimate	S.E.	C.R.	*p*	Label
DAÑO	<———	SUIC	.827	.069	11.991	***	
COMNERGZ	<———	DRUG	1.000				
COMMDIC	<———	DRUG	.349	.039	8.905	***	
CONSDRG	<———	DRUG	.662	.055	12.002	***	
CONTABAC	<———	DRUG	.629	.055	11.471	***	
CONSALCH	<———	DRUG	1.153	.087	13.189	***	
MALPAR	<———	MALEST	1.000				
MALFAM	<———	MALEST	2.164	.255	8.488	***	
MALSOCL	<———	MALEST	1.468	.180	8.140	***	
MALFINAZ	<———	MALEST	1.873	.225	8.342	***	
MALESSLD	<———	MALEST	1.698	.207	8.201	***	
VIDEOJG	<———	INTERN	1.000				
PORN	<———	INTERN	.756	.073	10.321	***	
APUESTAS	<———	INTERN	.312	.035	8.890	***	
RESDSLS	<———	INTERN	.113	.036	3.150	.002	
CFFAMILIA	<———	CONFADD	1.000				
CFPAREJA	<———	CONFADD	.876	.051	17.253	***	
CFAMIGS	<———	CONFADD	1.040	.046	22.525	***	
CFECNM	<———	CONFADD	1.095	.048	22.579	***	
CFLABR	<———	CONFADD	.886	.045	19.652	***	
CFACADM	<———	CONFADD	1.015	.046	22.038	***	
CFLEGAL	<———	CONFADD	.832	.039	21.143	***	
PROLGLS	<———	CONFDRUG	1.000				
PROBACDM	<———	CONFDRUG	1.265	.028	44.968	***	
PRMLABR	<———	CONFDRUG	.945	.027	35.012	***	
PROMECON	<———	CONFDRUG	1.247	.029	43.345	***	
CONFAMIG	<———	CONFDRUG	1.219	.029	42.660	***	
CONFPRJ	<———	CONFDRUG	.958	.033	28.679	***	
CONFFAM	<———	CONFDRUG	1.313	.033	40.369	***	

Standardized Regression Weights: (Group number 1 — Default model)

			Estimate
IDEACI	<———	SUIC	.643
INTENT	<———	SUIC	.534
DAÑO	<———	SUIC	.617
COMNERGZ	<———	DRUG	.489
COMMDIC	<———	DRUG	.342
CONSDRG	<———	DRUG	.540
CONTABAC	<———	DRUG	.498
CONSALCH	<———	DRUG	.686
MALPAR	<———	MALEST	.295
MALFAM	<———	MALEST	.688
MALSOCL	<———	MALEST	.542
MALFINAZ	<———	MALEST	.608
MALESSLD	<———	MALEST	.559
VIDEOJG	<———	INTERN	.621
PORN	<———	INTERN	.605
APUESTAS	<———	INTERN	.383
RESDSLS	<———	INTERN	.116
CFFAMILIA	<———	CONFADD	.611
CFPAREJA	<———	CONFADD	.518
CFAMIGS	<———	CONFADD	.838
CFECNM	<———	CONFADD	.841
CFLABR	<———	CONFADD	.637
CFACADM	<———	CONFADD	.615
CFLEGAL	<———	CONFADD	.731
PROLGLS	<———	CONFDRUG	.848

			Estimate
PROBACDM	<———	CONFDRUG	.900
PRMLABR	<———	CONFDRUG	.739
PROMECON	<———	CONFDRUG	.884
CONFAMIG	<———	CONFDRUG	.877
CONFPRJ	<———	CONFDRUG	.629
CONFFAM	<———	CONFDRUG	.852

Covariances: (Group number 1 — Default model)

			Estimate	S.E.	C.R.	*p*	Label
SUIC	<——>	DRUG	.032	.008	3.853	***	
SUIC	<——>	MALEST	.046	.007	6.199	***	
SUIC	<——>	INTERN	−.023	.014	−1.624	.104	
SUIC	<——>	CONFADD	.009	.005	1.974	.048	
SUIC	<——>	CONFDRUG	.017	.005	3.276	.001	
DRUG	<——>	MALEST	.020	.009	2.304	.021	
DRUG	<——>	INTERN	.213	.029	7.329	***	
DRUG	<——>	CONFADD	.058	.009	6.327	***	
DRUG	<——>	CONFDRUG	.132	.012	10.741	***	
MALEST	<——>	INTERN	−.031	.015	−2.022	.043	
MALEST	<——>	CONFADD	.031	.006	4.916	***	
MALEST	<——>	CONFDRUG	.022	.006	3.633	***	
INTERN	<——>	CONFADD	.100	.016	6.351	***	
INTERN	<——>	CONFDRUG	.087	.016	5.402	***	
CONFADD	<——>	CONFDRUG	.090	.007	12.947	***	
e24	<——>	e25	.042	.003	14.207	***	
e22	<——>	e27	.090	.005	16.538	***	
e19	<——>	e30	.175	.010	17.683	***	
e18	<——>	e23	.083	.008	9.836	***	

Correlations: (Group number 1 — Default model)

			Estimate
SUIC	<——>	DRUG	.176
SUIC	<——>	MALEST	.394
SUIC	<——>	INTERN	−.077
SUIC	<——>	CONFADD	.075
SUIC	<——>	CONFDRUG	.122
DRUG	<——>	MALEST	.099
DRUG	<——>	INTERN	.414
DRUG	<——>	CONFADD	.264
DRUG	<——>	CONFDRUG	.547
MALEST	<——>	INTERN	−.093
MALEST	<——>	CONFADD	.216
MALEST	<——>	CONFDRUG	.138
INTERN	<——>	CONFADD	.278
INTERN	<——>	CONFDRUG	.218
CONFADD	<——>	CONFDRUG	.532
e24	<——>	e25	.509
e22	<——>	e27	.575
e19	<——>	e30	.607
e18	<——>	e23	.322

Variances: (Group number 1 — Default model)

	Estimate	S.E.	C.R.	*p*	Label
SUIC	.103	.011	8.994	***	
DRUG	.313	.041	7.699	***	
MALEST	.132	.030	4.488	***	

	Estimate	S.E.	C.R.	*p*	Label
INTERN	.845	.105	8.027	***	
CONFADD	.154	.014	11.333	***	
CONFDRUG	.186	.010	19.018	***	
e1	.146	.010	14.622	***	
e2	.144	.008	19.183	***	
e3	.115	.007	15.837	***	
e4	.996	.046	21.771	***	
e5	.287	.012	23.546	***	
e6	.332	.016	20.792	***	
e7	.376	.017	21.622	***	
e8	.468	.029	16.118	***	
e9	1.392	.058	23.902	***	
e10	.689	.043	15.941	***	
e11	.688	.033	20.749	***	
e12	.793	.042	18.998	***	
e13	.839	.041	20.342	***	
e14	1.349	.096	14.089	***	
e15	.834	.056	14.794	***	
e16	.478	.022	22.076	***	
e17	.781	.032	24.597	***	
e18	.257	.011	23.054	***	
e19	.320	.013	23.788	***	
e20	.070	.004	17.968	***	
e21	.076	.004	17.779	***	
e22	.176	.008	22.878	***	
e23	.261	.011	23.026	***	
e24	.093	.004	21.521	***	
e25	.073	.003	21.522	***	
e26	.070	.004	19.370	***	
e27	.138	.006	23.291	***	
e28	.081	.004	20.251	***	
e29	.083	.004	20.565	***	
e30	.261	.011	23.989	***	
e31	.121	.006	21.430	***	

Matrices (Group number 1 — Default model)

Total Effects (Group number 1 — Default model)

	CONFDRUG	CONFADD	INTERN	MALEST	DRUG	SUIC
CONFFAM	1.313	.000	.000	.000	.000	.000
CONFPRJ	.958	.000	.000	.000	.000	.000
CONFAMIG	1.219	.000	.000	.000	.000	.000
PROMECON	1.247	.000	.000	.000	.000	.000
PRMLABR	.945	.000	.000	.000	.000	.000
PROBACDM	1.265	.000	.000	.000	.000	.000
PROLGLS	1.000	.000	.000	.000	.000	.000
CFLEGAL	.000	.832	.000	.000	.000	.000
CFACADM	.000	1.015	.000	.000	.000	.000
CFLABR	.000	.886	.000	.000	.000	.000
CFECNM	.000	1.095	.000	.000	.000	.000
CFAMIGS	.000	1.040	.000	.000	.000	.000
CFPAREJA	.000	.876	.000	.000	.000	.000
CFFAMILIA	.000	1.000	.000	.000	.000	.000
RESDSLS	.000	.000	.113	.000	.000	.000
APUESTAS	.000	.000	.312	.000	.000	.000
PORN	.000	.000	.756	.000	.000	.000
VIDEOJG	.000	.000	1.000	.000	.000	.000
MALESSLD	.000	.000	.000	1.698	.000	.000
MALFINAZ	.000	.000	.000	1.873	.000	.000
MALSOCL	.000	.000	.000	1.468	.000	.000
MALFAM	.000	.000	.000	2.164	.000	.000
MALPAR	.000	.000	.000	1.000	.000	.000
CONSALCH	.000	.000	.000	.000	1.153	.000

	CONFDRUG	CONFADD	INTERN	MALEST	DRUG	SUIC
CONTABAC	.000	.000	.000	.000	.629	.000
CONSDRG	.000	.000	.000	.000	.662	.000
COMMDIC	.000	.000	.000	.000	.349	.000
COMNERGZ	.000	.000	.000	.000	1.000	.000
DAÑO	.000	.000	.000	.000	.000	.827
INTENT	.000	.000	.000	.000	.000	.746
IDEACI	.000	.000	.000	.000	.000	1.000

Standardized Total Effects (Group number 1 — Default model)

	CONFDRUG	CONFADD	INTERN	MALEST	DRUG	SUIC
CONFFAM	.852	.000	.000	.000	.000	.000
CONFPRJ	.629	.000	.000	.000	.000	.000
CONFAMIG	.877	.000	.000	.000	.000	.000
PROMECON	.884	.000	.000	.000	.000	.000
PRMLABR	.739	.000	.000	.000	.000	.000
PROBACDM	.900	.000	.000	.000	.000	.000
PROLGLS	.848	.000	.000	.000	.000	.000
CFLEGAL	.000	.731	.000	.000	.000	.000
CFACADM	.000	.615	.000	.000	.000	.000
CFLABR	.000	.637	.000	.000	.000	.000
CFECNM	.000	.841	.000	.000	.000	.000
CFAMIGS	.000	.838	.000	.000	.000	.000
CFPAREJA	.000	.518	.000	.000	.000	.000
CFFAMILIA	.000	.611	.000	.000	.000	.000
RESDSLS	.000	.000	.116	.000	.000	.000
APUESTAS	.000	.000	.383	.000	.000	.000
PORN	.000	.000	.605	.000	.000	.000
VIDEOJG	.000	.000	.621	.000	.000	.000
MALESSLD	.000	.000	.000	.559	.000	.000
MALFINAZ	.000	.000	.000	.608	.000	.000
MALSOCL	.000	.000	.000	.542	.000	.000
MALFAM	.000	.000	.000	.688	.000	.000
MALPAR	.000	.000	.000	.295	.000	.000
CONSALCH	.000	.000	.000	.000	.686	.000
CONTABAC	.000	.000	.000	.000	.498	.000
CONSDRG	.000	.000	.000	.000	.540	.000
COMMDIC	.000	.000	.000	.000	.342	.000
COMNERGZ	.000	.000	.000	.000	.489	.000
DAÑO	.000	.000	.000	.000	.000	.617
INTENT	.000	.000	.000	.000	.000	.534
IDEACI	.000	.000	.000	.000	.000	.643

Direct Effects (Group number 1 — Default model)

	CONFDRUG	CONFADD	INTERN	MALEST	DRUG	SUIC
CONFFAM	1.313	.000	.000	.000	.000	.000
CONFPRJ	.958	.000	.000	.000	.000	.000
CONFAMIG	1.219	.000	.000	.000	.000	.000
PROMECON	1.247	.000	.000	.000	.000	.000
PRMLABR	.945	.000	.000	.000	.000	.000
PROBACDM	1.265	.000	.000	.000	.000	.000
PROLGLS	1.000	.000	.000	.000	.000	.000
CFLEGAL	.000	.832	.000	.000	.000	.000
CFACADM	.000	1.015	.000	.000	.000	.000
CFLABR	.000	.886	.000	.000	.000	.000
CFECNM	.000	1.095	.000	.000	.000	.000
CFAMIGS	.000	1.040	.000	.000	.000	.000
CFPAREJA	.000	.876	.000	.000	.000	.000
CFFAMILIA	.000	1.000	.000	.000	.000	.000
RESDSLS	.000	.000	.113	.000	.000	.000
APUESTAS	.000	.000	.312	.000	.000	.000
PORN	.000	.000	.756	.000	.000	.000
VIDEOJG	.000	.000	1.000	.000	.000	.000

	CONFDRUG	CONFADD	INTERN	MALEST	DRUG	SUIC
MALESSLD	.000	.000	.000	1.698	.000	.000
MALFINAZ	.000	.000	.000	1.873	.000	.000
MALSOCL	.000	.000	.000	1.468	.000	.000
MALFAM	.000	.000	.000	2.164	.000	.000
MALPAR	.000	.000	.000	1.000	.000	.000
CONSALCH	.000	.000	.000	.000	1.153	.000
CONTABAC	.000	.000	.000	.000	.629	.000
CONSDRG	.000	.000	.000	.000	.662	.000
COMMDIC	.000	.000	.000	.000	.349	.000
COMNERGZ	.000	.000	.000	.000	1.000	.000
DAÑO	.000	.000	.000	.000	.000	.827
INTENT	.000	.000	.000	.000	.000	.746
IDEACI	.000	.000	.000	.000	.000	1.000

Standardized Direct Effects (Group number 1 — Default model)

	CONFDRUG	CONFADD	INTERN	MALEST	DRUG	SUIC
CONFFAM	.852	.000	.000	.000	.000	.000
CONFPRJ	.629	.000	.000	.000	.000	.000
CONFAMIG	.877	.000	.000	.000	.000	.000
PROMECON	.884	.000	.000	.000	.000	.000
PRMLABR	.739	.000	.000	.000	.000	.000
PROBACDM	.900	.000	.000	.000	.000	.000
PROLGLS	.848	.000	.000	.000	.000	.000
CFLEGAL	.000	.731	.000	.000	.000	.000
CFACADM	.000	.615	.000	.000	.000	.000
CFLABR	.000	.637	.000	.000	.000	.000
CFECNM	.000	.841	.000	.000	.000	.000
CFAMIGS	.000	.838	.000	.000	.000	.000
CFPAREJA	.000	.518	.000	.000	.000	.000
CFFAMILIA	.000	.611	.000	.000	.000	.000
RESDSLS	.000	.000	.116	.000	.000	.000
APUESTAS	.000	.000	.383	.000	.000	.000
PORN	.000	.000	.605	.000	.000	.000
VIDEOJG	.000	.000	.621	.000	.000	.000
MALESSLD	.000	.000	.000	.559	.000	.000
MALFINAZ	.000	.000	.000	.608	.000	.000
MALSOCL	.000	.000	.000	.542	.000	.000
MALFAM	.000	.000	.000	.688	.000	.000
MALPAR	.000	.000	.000	.295	.000	.000
CONSALCH	.000	.000	.000	.000	.686	.000
CONTABAC	.000	.000	.000	.000	.498	.000
CONSDRG	.000	.000	.000	.000	.540	.000
COMMDIC	.000	.000	.000	.000	.342	.000
COMNERGZ	.000	.000	.000	.000	.489	.000
DAÑO	.000	.000	.000	.000	.000	.617
INTENT	.000	.000	.000	.000	.000	.534
IDEACI	.000	.000	.000	.000	.000	.643

Modification Indices (Group number 1 — Default model)

Covariances: (Group number 1 — Default model)

	M.I.	Par Change
e31 <---> DRUG	9.675	.020
e31 <---> SUIC	5.335	.009
e30 <---> SUIC	5.629	.011
e29 <---> e31	7.350	.009
e29 <---> e30	4.358	.008
e28 <---> SUIC	4.525	−.007
e27 <---> DRUG	8.053	−.015
e27 <---> e31	24.719	−.017
e27 <---> e29	20.667	−.013

			M.I.	Par Change
e27	<--->	e28	4.793	.006
e26	<--->	e31	14.932	.012
e26	<--->	e30	8.045	−.010
e25	<--->	SUIC	4.151	−.006
e25	<--->	e31	16.268	−.011
e25	<--->	e27	39.359	.014
e24	<--->	CONFDRUG	4.011	.006
e23	<--->	e28	4.997	−.010
e23	<--->	e26	16.665	.017
e22	<--->	MALEST	5.181	−.010
e22	<--->	e25	4.895	−.006
e22	<--->	e24	14.751	.011
e21	<--->	SUIC	6.123	−.009
e21	<--->	e31	13.033	−.012
e21	<--->	e29	18.380	−.012
e21	<--->	e28	48.147	.020
e20	<--->	e29	14.105	.010
e20	<--->	e22	6.826	−.008
e19	<--->	INTERN	5.377	−.035
e19	<--->	e29	4.458	−.009
e19	<--->	e22	14.938	.019
e18	<--->	INTERN	14.985	.061
e18	<--->	MALEST	7.361	.015
e18	<--->	SUIC	16.733	.022
e18	<--->	e31	4.391	.011
e18	<--->	e26	6.887	−.011
e18	<--->	e25	5.488	.008
e18	<--->	e24	12.642	−.014
e18	<--->	e22	24.106	−.025
e18	<--->	e20	25.380	.021
e17	<--->	CONFADD	5.960	.022
e17	<--->	MALEST	13.793	.038
e17	<--->	e21	7.009	−.021
e17	<--->	e18	13.149	.045
e16	<--->	INTERN	6.673	−.058
e16	<--->	MALEST	7.455	−.023
e16	<--->	DRUG	8.945	.036
e16	<--->	SUIC	7.159	−.021
e16	<--->	e28	4.825	.014
e16	<--->	e23	7.784	−.028
e16	<--->	e21	21.952	.030
e16	<--->	e18	6.626	−.026
e16	<--->	e17	4.050	−.036
e15	<--->	MALEST	7.440	.033
e15	<--->	e26	4.636	.019
e15	<--->	e23	4.247	.030
e15	<--->	e21	4.488	−.020
e15	<--->	e16	4.809	−.046
e14	<--->	DRUG	5.561	−.053
e14	<--->	e26	6.617	−.029
e14	<--->	e22	6.241	−.035
e14	<--->	e19	6.976	−.047
e14	<--->	e18	17.043	.076
e13	<--->	CONFDRUG	5.285	−.022
e13	<--->	DRUG	5.989	−.041
e13	<--->	SUIC	4.299	−.022
e13	<--->	e31	5.414	−.024

			M.I.	Par Change
e12	<--->	CONFADD	4.558	.021
e12	<--->	SUIC	6.175	-.027
e12	<--->	e28	10.711	.028
e12	<--->	e22	4.013	.020
e12	<--->	e21	15.364	.034
e12	<--->	e17	8.307	.071
e12	<--->	e16	5.131	.045
e12	<--->	e13	16.623	.109
e11	<--->	e25	6.424	-.016
e11	<--->	e22	5.619	-.022
e11	<--->	e20	7.100	.020
e11	<--->	e19	7.580	-.032
e11	<--->	e16	11.858	-.062
e11	<--->	e14	6.517	.085
e10	<--->	DRUG	4.704	.035
e10	<--->	SUIC	11.996	.036
e10	<--->	e31	6.148	.025
e10	<--->	e30	9.962	-.036
e10	<--->	e22	11.179	-.033
e10	<--->	e18	11.875	.046
e10	<--->	e15	6.913	.074
e10	<--->	e12	5.100	-.058
e10	<--->	e11	4.162	.048
e9	<--->	e30	41.768	.092
e9	<--->	e29	4.058	-.022
e9	<--->	e22	11.811	.043
e9	<--->	e21	11.497	-.037
e9	<--->	e19	42.451	.103
e8	<--->	CONFDRUG	48.427	.054
e8	<--->	DRUG	15.380	-.047
e8	<--->	e31	7.892	.023
e8	<--->	e28	13.662	.026
e8	<--->	e17	11.561	.068
e8	<--->	e16	6.708	.042
e8	<--->	e14	4.903	-.066
e7	<--->	CONFDRUG	17.073	-.026
e7	<--->	DRUG	9.993	.033
e7	<--->	e30	4.391	.016
e7	<--->	e14	6.951	-.064
e7	<--->	e8	5.825	-.034
e6	<--->	CONFDRUG	18.885	-.026
e6	<--->	DRUG	7.622	.027
e6	<--->	e28	21.338	-.025
e6	<--->	e22	6.133	.016
e6	<--->	e8	10.387	-.043
e6	<--->	e7	81.888	.101
e5	<--->	CONFDRUG	47.157	-.037
e5	<--->	CONFADD	8.048	.015
e5	<--->	DRUG	19.800	.041
e5	<--->	e28	5.252	-.011
e5	<--->	e17	4.118	-.028
e5	<--->	e8	7.761	-.034
e5	<--->	e7	14.372	.038
e5	<--->	e6	34.578	.056
e4	<--->	CONFDRUG	8.572	.030
e4	<--->	INTERN	6.499	.086
e4	<--->	DRUG	8.045	-.049

			M.I.	Par Change
e4	<——>	e16	7.751	.059
e4	<——>	e14	6.084	.098
e4	<——>	e7	10.800	—.063
e4	<——>	e6	9.648	—.056
e3	<——>	e31	8.928	.012
e3	<——>	e25	10.260	—.009
e3	<——>	e21	8.151	—.010
e3	<——>	e18	12.499	.019
e3	<——>	e16	4.405	—.016
e3	<——>	e12	8.291	—.030
e3	<——>	e10	5.728	.025
e2	<——>	CONFADD	5.376	.009
e2	<——>	MALEST	11.547	—.016
e2	<——>	e16	6.316	.021
e2	<——>	e11	9.243	—.031
e2	<——>	e9	7.285	.038
e1	<——>	MALEST	6.345	.013
e1	<——>	e23	8.697	.018
e1	<——>	e19	4.302	—.012
e1	<——>	e16	9.392	—.028
e1	<——>	e15	5.869	.031
e1	<——>	e10	6.710	.031

Variances: (Group number 1 — Default model)

	M.I.	Par Change

Regression Weights: (Group number 1 — Default model)

			M.I.	Par Change
CONFFAM	<———	DRUG	6.399	.056
CONFFAM	<———	SUIC	8.562	.120
CONFFAM	<———	PRMLABR	10.201	—.061
CONFFAM	<———	MALFAM	5.432	.022
CONFFAM	<———	CONSALCH	10.583	.037
CONFFAM	<———	DAÑO	12.545	.087
CONFPRJ	<———	DRUG	4.253	.052
CONFPRJ	<———	SUIC	5.931	.112
CONFPRJ	<———	MALPAR	38.250	.060
CONFPRJ	<———	CONTABAC	6.793	.044
CONFPRJ	<———	CONSDRG	5.202	.040
CONFPRJ	<———	INTENT	5.302	.061
CONFPRJ	<———	IDEACI	5.762	.057
CONFAMIG	<———	PRMLABR	8.474	—.047
CONFAMIG	<———	CFECNM	6.753	—.046
CONFAMIG	<———	MALPAR	5.038	—.016
CONFAMIG	<———	COMNERGZ	4.566	.017
PROMECON	<———	CFECNM	15.388	.069
PROMECON	<———	MALFINAZ	7.611	.022
PROMECON	<———	CONTABAC	4.776	—.028
PROMECON	<———	CONSDRG	19.532	—.057
PROMECON	<———	COMMDIC	6.734	—.041
PRMLABR	<———	CONFFAM	5.956	—.033
PRMLABR	<———	CONFPRJ	4.363	.029
PRMLABR	<———	CONFAMIG	4.065	—.031
PRMLABR	<———	PROLGLS	13.210	.065
PRMLABR	<———	CFLEGAL	4.660	.044
PRMLABR	<———	CONSALCH	4.096	—.020

			M.I.	Par Change
PROBACDM	<———	CFACADM	12.226	.046
PROBACDM	<———	VIDEOJG	5.408	−.013
PROLGLS	<———	MALEST	5.247	−.054
PROLGLS	<———	SUIC	7.824	−.078
PROLGLS	<———	PRMLABR	15.743	.052
PROLGLS	<———	MALSOCL	9.364	−.023
PROLGLS	<———	DAÑO	13.161	−.061
CFLEGAL	<———	CONFDRUG	5.256	.044
CFLEGAL	<———	DRUG	6.186	.043
CFLEGAL	<———	CONFFAM	7.855	.034
CFLEGAL	<———	CONFPRJ	9.299	.038
CFLEGAL	<———	CONFAMIG	4.498	.029
CFLEGAL	<———	PRMLABR	12.984	.053
CFLEGAL	<———	CFLABR	12.932	.054
CFLEGAL	<———	CFFAMILIA	9.928	−.040
CFLEGAL	<———	CONSALCH	6.006	.021
CFLEGAL	<———	CONTABAC	4.153	.024
CFACADM	<———	APUESTAS	5.405	−.043
CFACADM	<———	MALSOCL	4.446	.030
CFACADM	<———	IDEACI	7.395	.076
CFLABR	<———	CONFPRJ	4.713	.034
CFLABR	<———	CFPAREJA	19.560	.069
CFLABR	<———	CFFAMILIA	12.412	−.057
CFLABR	<———	VIDEOJG	4.011	−.014
CFLABR	<———	MALSOCL	7.041	−.028
CFLABR	<———	MALFAM	9.560	−.028
CFLABR	<———	MALPAR	7.934	.024
CFLABR	<———	CONSDRG	4.435	.032
CFECNM	<———	SUIC	7.174	−.093
CFECNM	<———	CONFPRJ	6.161	−.034
CFECNM	<———	CONFAMIG	4.137	−.031
CFECNM	<———	PROMECON	7.917	.042
CFECNM	<———	CFPAREJA	6.625	−.035
CFECNM	<———	RESDSLS	6.720	−.026
CFECNM	<———	APUESTAS	18.352	.052
CFECNM	<———	MALFINAZ	5.165	.018
CFECNM	<———	MALSOCL	4.349	−.019
CFECNM	<———	MALPAR	12.086	−.025
CFECNM	<———	DAÑO	11.061	−.070
CFAMIGS	<———	PRMLABR	4.310	−.033
CFAMIGS	<———	CFLABR	7.426	−.043
CFAMIGS	<———	CFFAMILIA	18.942	.059
CFPAREJA	<———	INTERN	4.602	−.039
CFPAREJA	<———	CFLABR	13.891	.091
CFPAREJA	<———	VIDEOJG	8.128	−.026
CFPAREJA	<———	MALPAR	39.067	.067
CFFAMILIA	<———	INTERN	4.368	.040
CFFAMILIA	<———	MALEST	12.896	.161
CFFAMILIA	<———	SUIC	22.022	.251
CFFAMILIA	<———	CFLABR	15.669	−.101
CFFAMILIA	<———	CFAMIGS	5.803	.069
CFFAMILIA	<———	RESDSLS	14.311	.059
CFFAMILIA	<———	VIDEOJG	14.751	.036
CFFAMILIA	<———	MALSOCL	6.458	.036
CFFAMILIA	<———	MALFAM	18.230	.052
CFFAMILIA	<———	DAÑO	23.078	.155
CFFAMILIA	<———	IDEACI	13.887	.104

			M.I.	Par Change
RESDSLS	<———	CONFDRUG	15.644	.237
RESDSLS	<———	CONFADD	20.268	.306
RESDSLS	<———	MALEST	31.065	.454
RESDSLS	<———	DRUG	10.508	.172
RESDSLS	<———	SUIC	14.377	.369
RESDSLS	<———	CONFFAM	13.658	.140
RESDSLS	<———	CONFPRJ	7.389	.105
RESDSLS	<———	CONFAMIG	11.197	.141
RESDSLS	<———	PROMECON	10.481	.134
RESDSLS	<———	PRMLABR	13.610	.169
RESDSLS	<———	PROBACDM	12.939	.150
RESDSLS	<———	PROLGLS	11.615	.169
RESDSLS	<———	CFLEGAL	15.646	.224
RESDSLS	<———	CFACADM	21.904	.183
RESDSLS	<———	CFLABR	13.442	.170
RESDSLS	<———	CFECNM	5.518	.116
RESDSLS	<———	CFAMIGS	12.998	.187
RESDSLS	<———	CFPAREJA	5.861	.092
RESDSLS	<———	CFFAMILIA	35.380	.234
RESDSLS	<———	MALESSLD	14.154	.086
RESDSLS	<———	MALFINAZ	24.776	.112
RESDSLS	<———	MALSOCL	6.871	.067
RESDSLS	<———	MALFAM	10.547	.072
RESDSLS	<———	MALPAR	6.293	.051
RESDSLS	<———	CONSALCH	16.335	.109
RESDSLS	<———	COMNERGZ	7.027	.058
RESDSLS	<———	IDEACI	8.891	.151
APUESTAS	<———	CONFDRUG	4.050	.098
APUESTAS	<———	MALEST	7.926	−.185
APUESTAS	<———	DRUG	5.940	.104
APUESTAS	<———	SUIC	8.239	−.226
APUESTAS	<———	CONFPRJ	5.181	.071
APUESTAS	<———	PROMECON	7.131	.090
APUESTAS	<———	PROLGLS	4.717	.087
APUESTAS	<———	CFACADM	4.489	−.067
APUESTAS	<———	CFECNM	11.282	.134
APUESTAS	<———	CFPAREJA	4.129	.063
APUESTAS	<———	CFFAMILIA	4.053	−.064
APUESTAS	<———	MALESSLD	6.399	−.047
APUESTAS	<———	MALSOCL	16.167	−.083
APUESTAS	<———	MALFAM	6.146	−.044
APUESTAS	<———	CONSALCH	9.371	.066
APUESTAS	<———	COMNERGZ	11.127	.059
APUESTAS	<———	DAÑO	8.376	−.137
APUESTAS	<———	IDEACI	12.450	−.144
PORN	<———	MALEST	10.352	.307
PORN	<———	SUIC	10.284	.365
PORN	<———	MALSOCL	7.031	.079
PORN	<———	MALFAM	12.516	.091
PORN	<———	CONTABAC	4.109	.085
PORN	<———	DAÑO	4.469	.145
PORN	<———	IDEACI	10.737	.194
VIDEOJG	<———	CONFDRUG	6.311	−.227
VIDEOJG	<———	MALEST	5.949	−.299
VIDEOJG	<———	DRUG	10.433	−.257
VIDEOJG	<———	SUIC	4.390	−.307
VIDEOJG	<———	CONFFAM	6.823	−.149

			M.I.	Par Change
VIDEOJG	<---	PROMECON	6.399	-.158
VIDEOJG	<---	PRMLABR	4.417	-.145
VIDEOJG	<---	PROBACDM	10.278	-.201
VIDEOJG	<---	CFLABR	4.889	-.154
VIDEOJG	<---	CFPAREJA	5.814	-.138
VIDEOJG	<---	CFFAMILIA	8.675	.175
VIDEOJG	<---	MALFINAZ	7.355	-.092
VIDEOJG	<---	MALFAM	4.138	-.067
VIDEOJG	<---	MALPAR	5.387	-.071
VIDEOJG	<---	CONSALCH	10.832	-.133
VIDEOJG	<---	CONTABAC	12.618	-.191
MALESSLD	<---	CONFDRUG	25.061	-.332
MALESSLD	<---	CONFADD	11.305	-.253
MALESSLD	<---	INTERN	18.374	-.166
MALESSLD	<---	DRUG	33.025	-.337
MALESSLD	<---	SUIC	5.504	-.252
MALESSLD	<---	CONFFAM	28.085	-.223
MALESSLD	<---	CONFPRJ	8.316	-.123
MALESSLD	<---	CONFAMIG	16.820	-.191
MALESSLD	<---	PROMECON	22.862	-.219
MALESSLD	<---	PRMLABR	10.282	-.162
MALESSLD	<---	PROBACDM	19.442	-.203
MALESSLD	<---	PROLGLS	14.487	-.209
MALESSLD	<---	CFLEGAL	4.100	-.127
MALESSLD	<---	CFLABR	4.154	-.104
MALESSLD	<---	CFECNM	7.084	-.146
MALESSLD	<---	CFAMIGS	8.883	-.171
MALESSLD	<---	CFFAMILIA	4.085	-.088
MALESSLD	<---	APUESTAS	7.073	-.099
MALESSLD	<---	PORN	8.226	-.070
MALESSLD	<---	VIDEOJG	5.912	-.046
MALESSLD	<---	MALFINAZ	9.024	.075
MALESSLD	<---	CONSALCH	16.216	-.120
MALESSLD	<---	CONTABAC	9.900	-.124
MALESSLD	<---	CONSDRG	6.974	-.108
MALESSLD	<---	COMNERGZ	15.711	-.097
MALESSLD	<---	INTENT	5.771	-.149
MALFINAZ	<---	CONFDRUG	4.033	.132
MALFINAZ	<---	CONFADD	8.075	.212
MALFINAZ	<---	SUIC	4.354	-.223
MALFINAZ	<---	PROMECON	9.764	.142
MALFINAZ	<---	PRMLABR	5.177	.114
MALFINAZ	<---	CFLABR	11.923	.176
MALFINAZ	<---	CFECNM	16.854	.223
MALFINAZ	<---	RESDSLS	8.594	.091
MALFINAZ	<---	APUESTAS	5.174	.084
MALFINAZ	<---	MALESSLD	10.262	.080
MALFINAZ	<---	DAÑO	9.303	-.196
MALSOCL	<---	PRMLABR	4.361	-.095
MALSOCL	<---	CFLABR	8.226	-.132
MALSOCL	<---	CFPAREJA	8.445	-.110
MALSOCL	<---	APUESTAS	7.361	-.091
MALSOCL	<---	VIDEOJG	5.435	.040
MALSOCL	<---	INTENT	6.862	-.147
MALFAM	<---	DRUG	8.377	.166
MALFAM	<---	SUIC	11.360	.354
MALFAM	<---	CONFFAM	4.202	.084

			M.I.	Par Change
MALFAM	<---	CONFPRJ	4.074	-.084
MALFAM	<---	CFLABR	5.589	-.118
MALFAM	<---	CFFAMILIA	6.462	.108
MALFAM	<---	PORN	5.964	.058
MALFAM	<---	CONTABAC	8.076	.110
MALFAM	<---	CONSDRG	5.183	.091
MALFAM	<---	COMMDIC	4.039	.096
MALFAM	<---	COMNERGZ	4.163	.049
MALFAM	<---	DAÑO	11.242	.213
MALFAM	<---	IDEACI	12.094	.190
MALPAR	<---	CONFDRUG	8.974	.243
MALPAR	<---	DRUG	7.598	.197
MALPAR	<---	CONFFAM	4.441	.108
MALPAR	<---	CONFPRJ	137.324	.608
MALPAR	<---	PROMECON	7.092	.149
MALPAR	<---	PRMLABR	16.871	.254
MALPAR	<---	PROBACDM	7.357	.152
MALPAR	<---	CFLABR	20.433	.283
MALPAR	<---	CFPAREJA	140.499	.610
MALPAR	<---	CONSALCH	4.651	.078
MALPAR	<---	COMNERGZ	5.864	.072
MALPAR	<---	INTENT	7.968	.214
CONSALCH	<---	CONFDRUG	32.021	.302
CONSALCH	<---	CONFADD	4.479	.128
CONSALCH	<---	CONFFAM	36.947	.205
CONSALCH	<---	CONFPRJ	18.938	.149
CONSALCH	<---	CONFAMIG	32.623	.214
CONSALCH	<---	PROMECON	41.652	.238
CONSALCH	<---	PRMLABR	8.203	.117
CONSALCH	<---	PROBACDM	20.115	.166
CONSALCH	<---	PROLGLS	21.440	.204
CONSALCH	<---	CFLEGAL	5.203	.115
CONSALCH	<---	RESDSLS	11.255	.085
CONSALCH	<---	APUESTAS	5.150	.068
CONSALCH	<---	CONTABAC	4.113	-.064
CONSALCH	<---	CONSDRG	6.788	-.085
CONSALCH	<---	COMMDIC	6.685	-.102
CONTABAC	<---	CONFDRUG	12.203	-.152
CONTABAC	<---	CONFFAM	9.081	-.083
CONTABAC	<---	CONFAMIG	10.935	-.101
CONTABAC	<---	PROMECON	14.265	-.114
CONTABAC	<---	PRMLABR	10.802	-.109
CONTABAC	<---	PROBACDM	10.044	-.096
CONTABAC	<---	PROLGLS	7.260	-.097
CONTABAC	<---	CFAMIGS	4.202	-.077
CONTABAC	<---	VIDEOJG	5.864	-.030
CONTABAC	<---	CONSDRG	52.034	.193
CONTABAC	<---	COMMDIC	12.274	.113
CONTABAC	<---	COMNERGZ	7.576	-.044
CONSDRG	<---	CONFDRUG	14.510	-.158
CONSDRG	<---	CONFADD	4.199	-.096
CONSDRG	<---	CONFFAM	7.225	-.071
CONSDRG	<---	CONFAMIG	11.474	-.099
CONSDRG	<---	PROMECON	27.575	-.151
CONSDRG	<---	PRMLABR	6.691	-.082
CONSDRG	<---	PROBACDM	11.653	-.098
CONSDRG	<---	PROLGLS	6.322	-.086

			M.I.	Par Change
CONSDRG	<---	CFFAMILIA	4.589	-.058
CONSDRG	<---	CONSALCH	4.324	-.039
CONSDRG	<---	CONTABAC	56.717	.186
CONSDRG	<---	COMMDIC	29.561	.166
CONSDRG	<---	COMNERGZ	6.786	-.040
COMMDIC	<---	CONFDRUG	22.884	-.177
COMMDIC	<---	CONFFAM	13.817	-.087
COMMDIC	<---	CONFPRJ	15.519	-.093
COMMDIC	<---	CONFAMIG	22.427	-.123
COMMDIC	<---	PROMECON	26.018	-.130
COMMDIC	<---	PRMLABR	10.388	-.091
COMMDIC	<---	PROBACDM	21.133	-.118
COMMDIC	<---	PROLGLS	19.872	-.136
COMMDIC	<---	RESDSLS	4.321	-.036
COMMDIC	<---	CONTABAC	9.865	.069
COMMDIC	<---	CONSDRG	21.799	.106
COMNERGZ	<---	CONFDRUG	5.785	.170
COMNERGZ	<---	CONFAMIG	8.512	.145
COMNERGZ	<---	PROMECON	4.436	.103
COMNERGZ	<---	PROBACDM	7.149	.131
COMNERGZ	<---	APUESTAS	9.349	.122
COMNERGZ	<---	VIDEOJG	6.829	.053
COMNERGZ	<---	CONTABAC	7.456	-.115
COMNERGZ	<---	CONSDRG	6.127	-.108
DAÑO	<---	PROLGLS	5.729	-.051
DAÑO	<---	CFLEGAL	7.294	-.066
DAÑO	<---	CFECNM	7.107	-.057
DAÑO	<---	APUESTAS	5.142	-.033
DAÑO	<---	MALFINAZ	4.490	-.021
INTENT	<---	CONFADD	5.864	.076
INTENT	<---	INTERN	4.807	.035
INTENT	<---	MALEST	6.271	-.094
INTENT	<---	CONFPRJ	7.759	.049
INTENT	<---	CFLABR	9.297	.065
INTENT	<---	CFECNM	4.933	.051
INTENT	<---	CFAMIGS	4.833	.053
INTENT	<---	CFPAREJA	9.243	.054
INTENT	<---	APUESTAS	8.429	.045
INTENT	<---	MALESSLD	6.582	-.027
INTENT	<---	MALSOCL	12.671	-.042
INTENT	<---	MALFAM	4.763	-.022
IDEACI	<---	MALEST	4.229	.083
IDEACI	<---	CFACADM	7.400	.053
IDEACI	<---	CFFAMILIA	4.703	.042
IDEACI	<---	APUESTAS	8.357	-.048
IDEACI	<---	MALSOCL	4.379	.027
IDEACI	<---	MALFAM	8.044	.031

Minimization History (Default model)

Iteration		Negative eigenvalues	Condition #	Smallest eigenvalue	Diameter	F	NTries	Ratio
0	e	16		-1.228	9999.000	16492.855	0	9999.000
1	e	16		-.311	2.375	9967.017	19	.416
2	e*	7		-.411	1.093	6290.224	5	.889
3	e*	1		-.082	.771	4476.844	5	.740
4	e	0	754.608		.907	2973.580	5	.855
5	e	0	432.335		1.211	2441.212	3	.000

Iteration		Negative eigenvalues	Condition #	Smallest eigenvalue	Diameter	F	NTries	Ratio
6	e	0	274.197		.568	2151.449	3	.000
7	e	0	407.616		1.044	1812.450	1	1.111
8	e	0	1052.518		.648	1738.664	1	1.160
9	e	0	2868.249		.465	1724.587	1	1.184
10	e	0	5132.305		.383	1721.769	1	1.136
11	e	0	9916.605		.188	1721.083	1	1.154
12	e	0	12653.065		.109	1721.007	1	1.071
13	e	0	13630.858		.014	1721.005	1	1.016
14	e	0	13512.626		.001	1721.005	1	1.000

Model Fit Summary

CMIN

Model	NPAR	CMIN	*df*	*p*	CMIN/DF
Default model	81	1721.005	415	.000	4.147
Saturated model	496	.000	0		
Independence model	31	16431.989	465	.000	35.338

RMR. GFI

Model	RMR	GFI	AGFI	PGFI
Default model	.036	.913	.897	.764
Saturated model	.000	1.000		
Independence model	.116	.363	.320	.340

Baseline Comparisons

Model	NFI Delta1	RFI rho1	IFI Delta2	TLI rho2	CFI
Default model	.895	.883	.918	.908	.918
Saturated model	1.000		1.000		1.000
Independence model	.000	.000	.000	.000	.000

Parsimony—Adjusted Measures

Model	PRATIO	PNFI	PCFI
Default model	.892	.799	.819
Saturated model	.000	.000	.000
Independence model	1.000	.000	.000

NCP

Model	NCP	LO 90	HI 90
Default model	1306.005	1181.743	1437.787
Saturated model	.000	.000	.000
Independence model	15966.989	15551.460	16388.856

FMIN

Model	FMIN	F0	LO 90	HI 90
Default model	1.398	1.061	.960	1.168
Saturated model	.000	.000	.000	.000
Independence model	13.348	12.971	12.633	13.313

RMSEA

Model	RMSEA	LO 90	HI 90	PCLOSE
Default model	.051	.048	.053	.350
Independence model	.167	.165	.169	.000

AIC

Model	AIC	BCC	BIC	CAIC
Default model	1883.005	1887.329	2297.433	2378.433
Saturated model	992.000	1018.475	3529.731	4025.731
Independence model	16493.989	16495.643	16652.597	16683.597

ECVI

Model	ECVI	LO 90	HI 90	MECVI
Default model	1.530	1.429	1.637	1.533
Saturated model	.806	.806	.806	.827
Independence model	13.399	13.061	13.742	13.400

HOELTER

Model	HOELTER .05	HOELTER .01
Default model	332	347
Independence model	39	41

Execution time summary

Minimization:	.019
Miscellaneous:	.850
Bootstrap:	.000
Total:	.869

Path Diagram: Confirmatory Factor Analysis

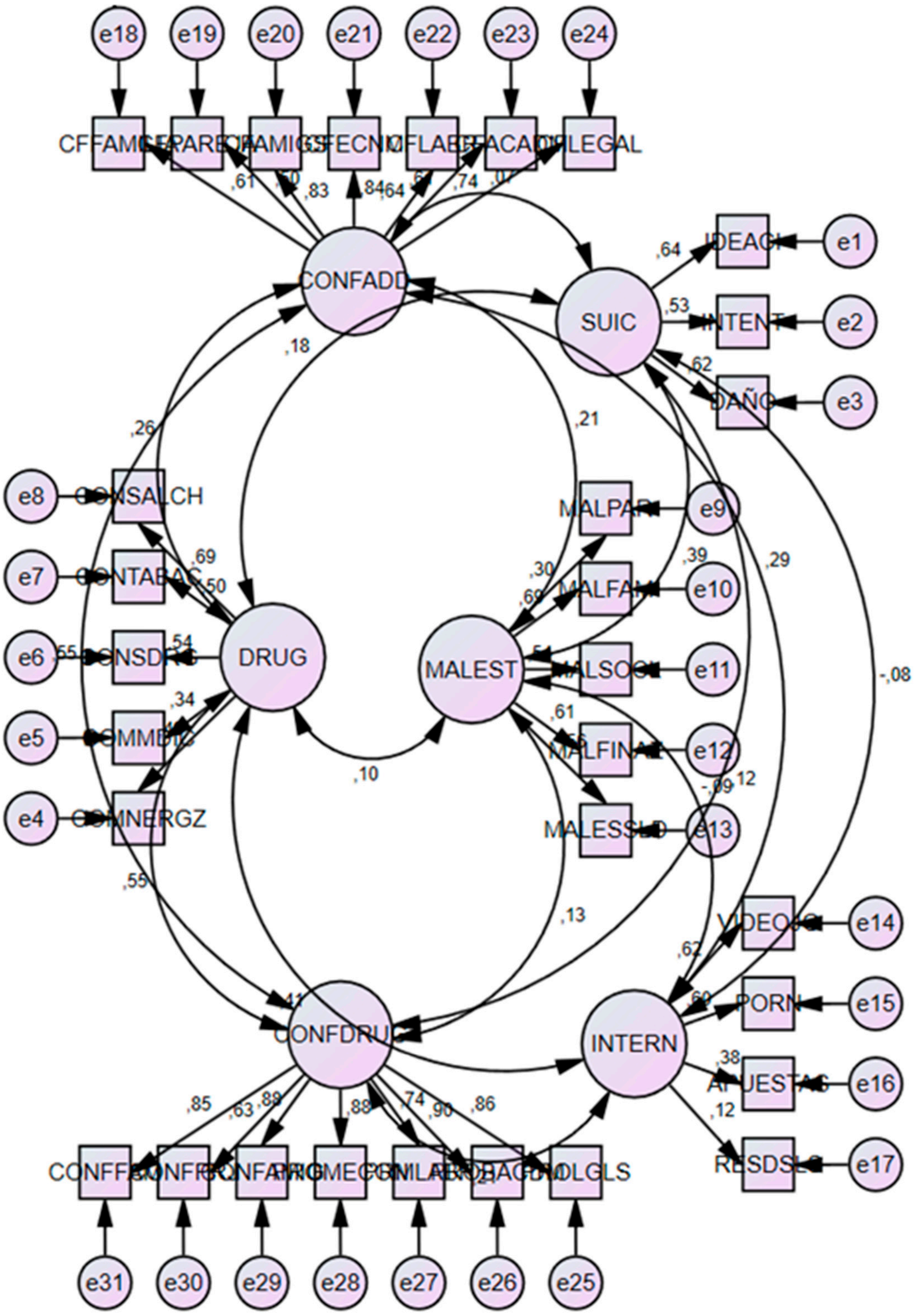

CONFIRMATORY FACTOR ANALYSIS WELL-BEING AND DISTRESS IN THE LAST WEEK

C:\Users\media\Dropbox\Mi PC (LAPTOP-LCRG33R5)\Desktop\2024\Articulos 2024\Salud mental 2024 1\Resultados junio final\Resultados 1.amw

Analysis Summary

Date and Time

Date: sábado, 22 de junio de 2024
Time: 11:18:07 a. m.

Title

Resultados 1: sábado, 22 de junio de 2024 11:18 a. m.

Groups

Group number 1 (Group number 1)

Notes for Group (Group number 1)

The model is recursive.
Sample size = 1232

Variable Summary (Group number 1)

Your model contains the following variables (Group number 1)

Observed, endogenous variables
NERVS
PRCUPC
ZOZ
TENSINE
TRIT
IRRIT
NODISFR
PERDDNT
TRAQUIL
ALGR
VITALI
OPTIM
PROBS
DOLRMU
DOLCA
COMRM
PROBLDI
Unobserved, exogenous variables
SINT
e1
e2
e3
e4
e5
e6
e7
e8
BIEN
e9
e10
e11
e12
FIS
e13
e14
e15
e16
e17

Variable counts (Group number 1)

Number of variables in your model:	37
Number of observed variables:	17
Number of unobserved variables:	20
Number of exogenous variables:	20
Number of endogenous variables:	17

Parameter Summary (Group number 1)

	Weights	Covariances	Variances	Means	Intercepts	Total
Fixed	20	0	0	0	0	20
Labeled	0	0	0	0	0	0
Unlabeled	14	5	20	0	0	39
Total	34	5	20	0	0	59

Models

Default model (Default model)

Notes for Model (Default model)

Computation of degrees of freedom (Default model)

Number of distinct sample moments: 153
Number of distinct parameters to be estimated: 39
Degrees of freedom (153 - 39): 114

Result (Default model)

Minimum was achieved
Chi-square = 867,788
Degrees of freedom = 114
Probability level = ,000

Group number 1 (Group number 1 - Default model)

Estimates (Group number 1 - Default model)

Scalar Estimates (Group number 1 - Default model)

Maximum Likelihood Estimates

Regression Weights: (Group number 1 — Default model)

			Estimate	S.E.	C.R.	*p*	Label
NERVS	<———	SINT	1.000				
PRCUPC	<———	SINT	1.030	.032	32.395	***	
ZOZ	<———	SINT	1.115	.047	23.783	***	
TENSINE	<———	SINT	1.132	.046	24.848	***	
TRIT	<———	SINT	1.174	.048	24.608	***	
IRRIT	<———	SINT	1.141	.049	23.098	***	
NODISFR	<———	SINT	.993	.046	21.785	***	
PERDDNT	<———	SINT	1.012	.047	21.684	***	
TRAQUIL	<———	BIEN	1.000				
ALGR	<———	BIEN	1.458	.076	19.174	***	
VITALI	<———	BIEN	1.307	.072	18.268	***	
OPTIM	<———	BIEN	1.154	.069	16.823	***	
PROBS	<———	FIS	1.000				
DOLRMU	<———	FIS	1.228	.060	20.565	***	
DOLCA	<———	FIS	1.300	.060	21.531	***	
COMRM	<———	FIS	1.183	.059	19.986	***	
PROBLDI	<———	FIS	1.123	.059	19.098	***	

Standardized Regression Weights: (Group number 1 — Default model)

			Estimate
NERVS	<———	SINT	.688
PRCUPC	<———	SINT	.758
ZOZ	<———	SINT	.751
TENSINE	<———	SINT	.789
TRIT	<———	SINT	.780
IRRIT	<———	SINT	.726
NODISFR	<———	SINT	.682
PERDDNT	<———	SINT	.679
TRAQUIL	<———	BIEN	.595
ALGR	<———	BIEN	.842
VITALI	<———	BIEN	.709
OPTIM	<———	BIEN	.625
PROBS	<———	FIS	.649
DOLRMU	<———	FIS	.715
DOLCA	<———	FIS	.762
COMRM	<———	FIS	.689
PROBLDI	<———	FIS	.651

Covariances: (Group number 1 — Default model)

			Estimate	S.E.	C.R.	*p*	Label
SINT	<——>	BIEN	−.093	.019	−4.763	***	
SINT	<——>	FIS	.503	.036	14.119	***	
BIEN	<——>	FIS	.000	.018	−.023	.982	
e7	<——>	e8	.439	.032	13.658	***	
e1	<——>	e2	.324	.027	12.190	***	

Correlations: (Group number 1 — Default model)

			Estimate
SINT	<——>	BIEN	−.165
SINT	<——>	FIS	.723
BIEN	<——>	FIS	−.001
e7	<——>	e8	.491
e1	<——>	e2	.450

Variances: (Group number 1 — Default model)

	Estimate	S.E.	C.R.	*p*	Label
SINT	.768	.058	13.176	***	
BIEN	.409	.039	10.418	***	
FIS	.630	.053	11.901	***	
e1	.857	.039	22.166	***	
e2	.603	.029	21.010	***	
e3	.740	.035	21.278	***	
e4	.597	.029	20.286	***	
e5	.681	.033	20.544	***	
e6	.895	.041	21.755	***	
e7	.870	.039	22.358	***	
e8	.920	.041	22.399	***	
e9	.744	.034	21.670	***	
e10	.355	.032	11.048	***	
e11	.691	.037	18.558	***	
e12	.851	.040	21.101	***	
e13	.865	.040	21.598	***	
e14	.906	.045	20.162	***	
e15	.768	.041	18.643	***	
e16	.975	.047	20.807	***	
e17	1.081	.050	21.570	***	

Matrices (Group number 1 — Default model)

Total Effects (Group number 1 — Default model)

	FIS	BIEN	SINT
PROBLDI	1.123	.000	.000
COMRM	1.183	.000	.000
DOLCA	1.300	.000	.000
DOLRMU	1.228	.000	.000
PROBS	1.000	.000	.000
OPTIM	.000	1.154	.000
VITALI	.000	1.307	.000
ALGR	.000	1.458	.000
TRAQUIL	.000	1.000	.000
PERDDNT	.000	.000	1.012
NODISFR	.000	.000	.993
IRRIT	.000	.000	1.141
TRIT	.000	.000	1.174
TENSINE	.000	.000	1.132
ZOZ	.000	.000	1.115
PRCUPC	.000	.000	1.030
NERVS	.000	.000	1.000

Standardized Total Effects (Group number 1 — Default model)

	FIS	BIEN	SINT
PROBLDI	.651	.000	.000
COMRM	.689	.000	.000
DOLCA	.762	.000	.000
DOLRMU	.715	.000	.000
PROBS	.649	.000	.000
OPTIM	.000	.625	.000
VITALI	.000	.709	.000
ALGR	.000	.842	.000
TRAQUIL	.000	.595	.000
PERDDNT	.000	.000	.679
NODISFR	.000	.000	.682
IRRIT	.000	.000	.726
TRIT	.000	.000	.780
TENSINE	.000	.000	.789
ZOZ	.000	.000	.751
PRCUPC	.000	.000	.758
NERVS	.000	.000	.688

Direct Effects (Group number 1 — Default model)

	FIS	BIEN	SINT
PROBLDI	1.123	.000	.000
COMRM	1.183	.000	.000
DOLCA	1.300	.000	.000
DOLRMU	1.228	.000	.000
PROBS	1.000	.000	.000
OPTIM	.000	1.154	.000
VITALI	.000	1.307	.000
ALGR	.000	1.458	.000
TRAQUIL	.000	1.000	.000
PERDDNT	.000	.000	1.012
NODISFR	.000	.000	.993
IRRIT	.000	.000	1.141
TRIT	.000	.000	1.174
TENSINE	.000	.000	1.132
ZOZ	.000	.000	1.115
PRCUPC	.000	.000	1.030
NERVS	.000	.000	1.000

Standardized Direct Effects (Group number 1 — Default model)

	FIS	BIEN	SINT
PROBLDI	.651	.000	.000
COMRM	.689	.000	.000
DOLCA	.762	.000	.000
DOLRMU	.715	.000	.000
PROBS	.649	.000	.000
OPTIM	.000	.625	.000
VITALI	.000	.709	.000
ALGR	.000	.842	.000
TRAQUIL	.000	.595	.000
PERDDNT	.000	.000	.679
NODISFR	.000	.000	.682
IRRIT	.000	.000	.726
TRIT	.000	.000	.780
TENSINE	.000	.000	.789
ZOZ	.000	.000	.751
PRCUPC	.000	.000	.758
NERVS	.000	.000	.688

			M.I.	Par Change
e16	<——>	BIEN	4.137	.043
e16	<——>	e17	20.281	.149
e15	<——>	e17	11.611	−.104
e14	<——>	SINT	6.290	−.052
e14	<——>	e17	8.599	−.094
e14	<——>	e16	7.801	−.086
e14	<——>	e15	27.676	.149
e13	<——>	SINT	4.375	.042
e13	<——>	e17	15.472	.121
e13	<——>	e16	14.168	−.111
e13	<——>	e15	10.672	−.089
e12	<——>	FIS	11.055	.063
e12	<——>	SINT	5.484	.047
e12	<——>	e13	11.521	.093
e11	<——>	SINT	18.788	.081
e11	<——>	e15	5.711	−.061
e11	<——>	e12	16.550	.103
e9	<——>	FIS	6.288	−.044
e9	<——>	SINT	28.812	−.099
e9	<——>	e12	4.832	−.055
e8	<——>	FIS	19.265	.072
e8	<——>	SINT	10.271	−.055
e8	<——>	e16	5.103	.058
e7	<——>	e15	4.002	−.046
e7	<——>	e14	4.645	−.053
e6	<——>	FIS	18.184	.082
e6	<——>	SINT	8.854	−.060
e6	<——>	e17	5.184	.072
e6	<——>	e14	5.410	.069
e6	<——>	e13	4.005	.056
e6	<——>	e11	5.244	.060
e6	<——>	e10	6.750	−.059
e6	<——>	e7	25.164	.118
e5	<——>	BIEN	8.120	−.050
e5	<——>	e14	9.424	−.081
e5	<——>	e13	4.916	.056
e5	<——>	e12	4.840	−.055
e5	<——>	e8	11.703	.074
e5	<——>	e6	7.826	.071
e4	<——>	e15	7.661	.065
e4	<——>	e14	6.725	−.065
e4	<——>	e8	4.994	−.045
e4	<——>	e6	7.364	−.065
e4	<——>	e5	11.677	.072
e3	<——>	FIS	19.132	−.077
e3	<——>	BIEN	7.648	.050
e3	<——>	SINT	11.055	.061
e3	<——>	e15	8.752	−.076
e3	<——>	e13	4.887	−.057
e3	<——>	e11	12.143	.084
e3	<——>	e6	14.099	−.098
e3	<——>	e5	32.391	−.132
e2	<——>	BIEN	4.513	.030
e2	<——>	e17	5.223	−.051
e2	<——>	e12	9.223	.061
e2	<——>	e8	10.688	−.057
e2	<——>	e6	4.754	−.045

	M.I.	Par Change
e2 <——> e5	6.998	−.048
e2 <——> e3	63.403	.149
e1 <——> e9	8.859	−.064
e1 <——> e6	16.262	−.096
e1 <——> e5	4.319	−.044
e1 <——> e3	61.798	.172

Variances: (Group number 1 — Default model)

	M.I.	Par Change

Regression Weights: (Group number 1 — Default model)

			M.I.	Par Change
PROBLDI	<———	COMRM	9.387	.071
PROBLDI	<———	PROBS	8.098	.073
PROBLDI	<———	OPTIM	4.031	−.053
COMRM	<———	PROBLDI	10.609	.072
COMRM	<———	PROBS	7.445	−.068
COMRM	<———	PERDDNT	5.798	.056
DOLCA	<———	PROBLDI	6.150	−.051
DOLCA	<———	DOLRMU	11.950	.071
DOLCA	<———	PROBS	5.678	−.055
DOLCA	<———	VITALI	5.046	−.053
DOLRMU	<———	PROBLDI	4.514	−.046
DOLRMU	<———	DOLCA	9.637	.068
DOLRMU	<———	NODISFR	4.951	−.052
DOLRMU	<———	TRIT	8.990	−.067
DOLRMU	<———	TENSINE	7.307	−.063
PROBS	<———	PROBLDI	8.060	.058
PROBS	<———	COMRM	6.557	−.053
PROBS	<———	OPTIM	5.667	.057
PROBS	<———	PERDDNT	6.232	.054
PROBS	<———	NODISFR	5.869	.053
PROBS	<———	IRRIT	4.694	.044
PROBS	<———	TRIT	4.885	.047
OPTIM	<———	FIS	41.828	.246
OPTIM	<———	SINT	34.870	.198
OPTIM	<———	PROBLDI	7.724	.057
OPTIM	<———	COMRM	21.470	.095
OPTIM	<———	DOLCA	23.267	.099
OPTIM	<———	DOLRMU	25.990	.104
OPTIM	<———	PROBS	39.835	.144
OPTIM	<———	VITALI	6.951	.062
OPTIM	<———	PERDDNT	7.147	.057
OPTIM	<———	NODISFR	7.835	.061
OPTIM	<———	IRRIT	23.325	.098
OPTIM	<———	TRIT	9.659	.066
OPTIM	<———	TENSINE	24.793	.111
OPTIM	<———	ZOZ	25.635	.109
OPTIM	<———	PRCUPC	39.395	.147
OPTIM	<———	NERVS	21.521	.102
VITALI	<———	FIS	7.170	.096
VITALI	<———	SINT	19.375	.138
VITALI	<———	PROBLDI	6.180	.047
VITALI	<———	COMRM	4.943	.043
VITALI	<———	PROBS	4.519	.045
VITALI	<———	OPTIM	9.203	.067

			M.I.	Par Change
VITALI	<———	PERDDNT	6.795	.052
VITALI	<———	NODISFR	12.991	.074
VITALI	<———	IRRIT	20.444	.086
VITALI	<———	TRIT	8.879	.059
VITALI	<———	TENSINE	13.812	.077
VITALI	<———	ZOZ	27.976	.106
VITALI	<———	PRCUPC	5.804	.053
VITALI	<———	NERVS	5.704	.049
ALGR	<———	PROBLDI	4.592	−.035
ALGR	<———	IRRIT	8.603	−.048
ALGR	<———	ZOZ	5.828	−.042
TRAQUIL	<———	FIS	69.587	−.295
TRAQUIL	<———	SINT	80.652	−.279
TRAQUIL	<———	PROBLDI	17.685	−.079
TRAQUIL	<———	COMRM	25.057	−.095
TRAQUIL	<———	DOLCA	46.260	−.130
TRAQUIL	<———	DOLRMU	40.913	−.121
TRAQUIL	<———	PROBS	37.085	−.129
TRAQUIL	<———	PERDDNT	35.989	−.119
TRAQUIL	<———	NODISFR	35.272	−.120
TRAQUIL	<———	IRRIT	42.438	−.122
TRAQUIL	<———	TRIT	42.735	−.128
TRAQUIL	<———	TENSINE	54.225	−.151
TRAQUIL	<———	ZOZ	35.746	−.119
TRAQUIL	<———	PRCUPC	76.331	−.190
TRAQUIL	<———	NERVS	79.399	−.181
PERDDNT	<———	FIS	6.143	.082
PERDDNT	<———	COMRM	9.602	.055
PERDDNT	<———	DOLCA	4.320	.037
PERDDNT	<———	DOLRMU	6.942	.047
PERDDNT	<———	PROBS	6.255	.049
PERDDNT	<———	PRCUPC	8.124	−.058
PERDDNT	<———	NERVS	6.242	−.047
NODISFR	<———	IRRIT	10.620	.056
IRRIT	<———	FIS	6.413	.099
IRRIT	<———	PROBLDI	9.940	.066
IRRIT	<———	DOLRMU	10.020	.066
IRRIT	<———	PROBS	8.731	.069
IRRIT	<———	PERDDNT	13.178	.079
IRRIT	<———	NODISFR	24.284	.110
IRRIT	<———	ZOZ	5.472	−.051
IRRIT	<———	PRCUPC	8.167	−.069
IRRIT	<———	NERVS	16.181	−.090
TRIT	<———	BIEN	8.069	−.125
TRIT	<———	DOLRMU	5.083	−.042
TRIT	<———	OPTIM	10.428	−.070
TRIT	<———	VITALI	4.583	−.046
TRIT	<———	ALGR	5.774	−.056
TRIT	<———	PERDDNT	10.108	.062
TRIT	<———	NODISFR	4.249	.041
TRIT	<———	ZOZ	12.675	−.070
TRIT	<———	PRCUPC	6.528	−.055
TRIT	<———	NERVS	6.847	−.052
TENSINE	<———	PERDDNT	5.191	−.042
TENSINE	<———	TRIT	4.007	.037
ZOZ	<———	FIS	5.433	−.083
ZOZ	<———	BIEN	4.251	.093

			M.I.	Par Change
ZOZ	<---	PROBLDI	6.503	−.049
ZOZ	<---	DOLCA	11.198	−.065
ZOZ	<---	PROBS	8.909	−.064
ZOZ	<---	OPTIM	4.783	.049
ZOZ	<---	VITALI	12.641	.079
ZOZ	<---	NODISFR	4.277	−.043
ZOZ	<---	IRRIT	6.034	−.047
ZOZ	<---	TRIT	11.022	−.066
ZOZ	<---	PRCUPC	67.337	.181
ZOZ	<---	NERVS	83.988	.189
PRCUPC	<---	OPTIM	11.279	.058
PRCUPC	<---	ALGR	4.692	.040
PRCUPC	<---	PERDDNT	9.716	−.049
PRCUPC	<---	NODISFR	4.563	−.034
PRCUPC	<---	ZOZ	24.429	.078
NERVS	<---	TRAQUIL	7.549	−.061
NERVS	<---	IRRIT	6.856	−.045
NERVS	<---	ZOZ	23.642	.089

Minimization History (Default model)

Iteration		Negative eigenvalues	Condition #	Smallest eigenvalue	Diameter	F	NTries	Ratio
0	e	8		−1.272	9999.000	10129.936	0	9999.000
1	e	7		−.294	1.765	5851.533	19	.471
2	e*	3		−.307	.811	4131.630	5	.741
3	e*	1		−.020	.829	2476.119	5	.783
4	e	0	260.118		1.183	1315.985	6	.797
5	e	0	160.089		1.248	1315.709	1	.001
6	e	0	262.408		.561	921.787	1	1.142
7	e	0	336.602		.153	874.631	1	1.158
8	e	0	310.337		.135	868.187	1	1.130
9	e	0	298.820		.029	867.790	1	1.049
10	e	0	295.251		.003	867.788	1	1.005
11	e	0	303.210		.000	867.788	1	1.000

Model Fit Summary

CMIN

Model	NPAR	CMIN	*df*	*p*	CMIN/DF
Default model	39	867.788	114	.000	7.612
Saturated model	153	.000	0		
Independence model	17	10381.587	136	.000	76.335

RMR. GFI

Model	RMR	GFI	AGFI	PGFI
Default model	.098	.921	.894	.686
Saturated model	.000	1.000		
Independence model	.581	.313	.227	.278

Baseline Comparisons

Model	NFI Delta1	RFI rho1	IFI Delta2	TLI rho2	CFI
Default model	.916	.900	.927	.912	.926
Saturated model	1.000		1.000		1.000
Independence model	.000	.000	.000	.000	.000

Parsimony−Adjusted Measures

Model	PRATIO	PNFI	PCFI
Default model	.838	.768	.777

Model	PRATIO	PNFI	PCFI
Saturated model	.000	.000	.000
Independence model	1.000	.000	.000

NCP

Model	NCP	LO 90	HI 90
Default model	753.788	663.811	851.231
Saturated model	.000	.000	.000
Independence model	10245.587	9914.658	10582.819

FMIN

Model	FMIN	F0	LO 90	HI 90
Default model	.705	.612	.539	.691
Saturated model	.000	.000	.000	.000
Independence model	8.433	8.323	8.054	8.597

RMSEA

Model	RMSEA	LO 90	HI 90	PCLOSE
Default model	.073	.069	.078	.000
Independence model	.247	.243	.251	.000

AIC

Model	AIC	BCC	BIC	CAIC
Default model	945.788	946.945	1145.327	1184.327
Saturated model	306.000	310.541	1088.808	1241.808
Independence model	10415.587	10416.091	10502.566	10519.566

ECVI

Model	ECVI	LO 90	HI 90	MECVI
Default model	.768	.695	.847	.769
Saturated model	.249	.249	.249	.252
Independence model	8.461	8.192	8.735	8.461

HOELTER

Model	HOELTER .05	HOELTER .01
Default model	199	216
Independence model	20	22

Execution time summary

Minimization:	.018
Miscellaneous:	.386
Bootstrap:	.000
Total:	.404

Path Diagram: Confirmatory Factor Analysis

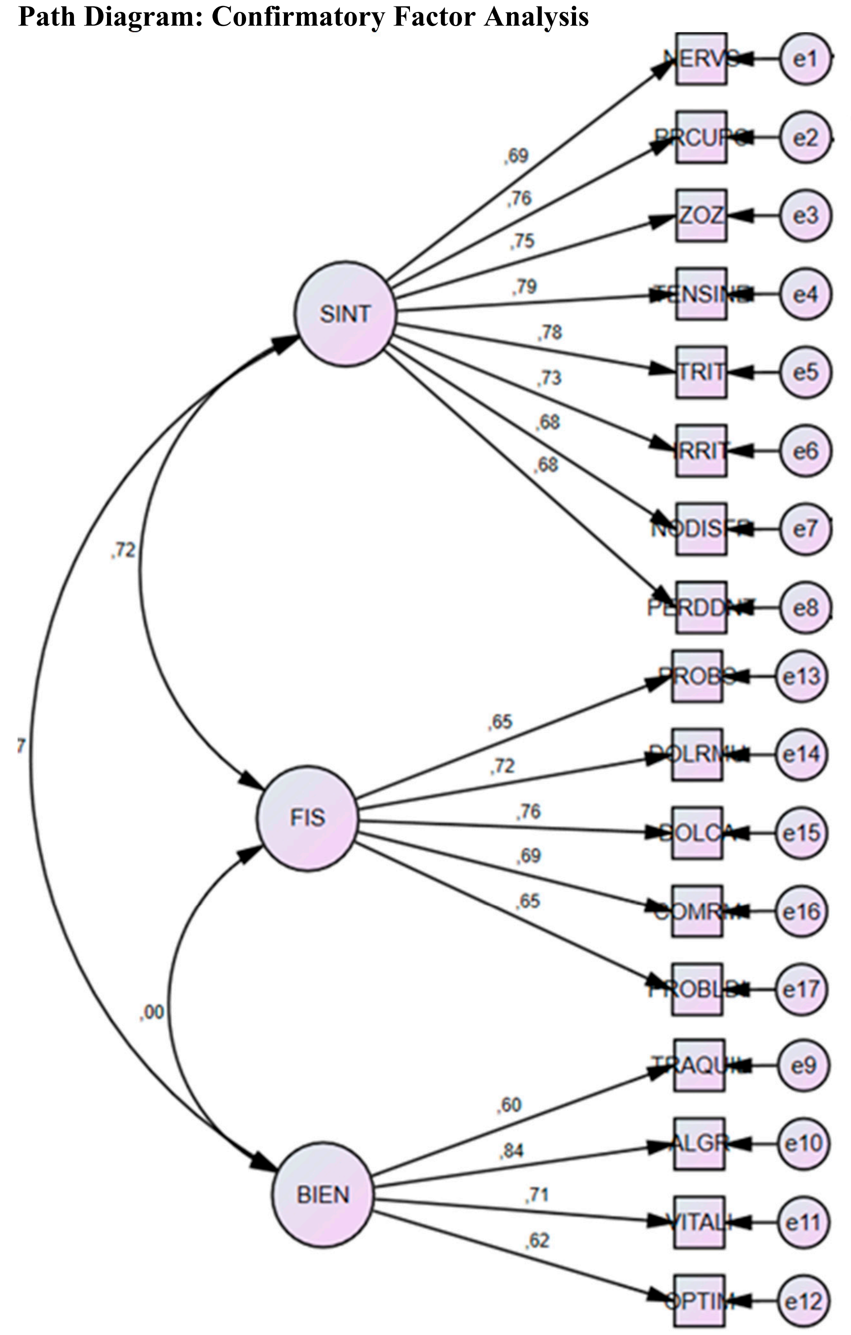

CONFIRMATORY FACTOR ANALYSIS SYMPTOMS IN 6 MONTHS

C:\Users\media\Dropbox\Mi PC (LAPTOP－LCRG33R5)\Desktop\2024\Articulos 2024\Salud mental 2024 1\Resultados junio final\Resutados 6 MESES.amw

Analysis Summary

Date and Time

Date: sábado. 22 de junio de 2024
Time: 1:05:13 p. m.

Title

Resutados 6 meses: sábado. 22 de junio de 2024 1:05 p. m.

Groups

Group number 1 (Group number 1)

Notes for Group (Group number 1)

The model is recursive.

Sample size = 1232

Variable Summary (Group number 1)

Your model contains the following variables (Group number 1)

Observed. endogenous variables
MESNR
MESP
MESZZ
MESTN
MESTRI
MESIR
MESDI
PERIN
MPROS
MDOLMU
MCABE
MCOME
MDIGE
MESTR
MALEG
MILUSI
MEVIT
Unobserved. exogenous variables
MALEST
e1
e2
e3
e4
e5
e6
e7
e8
FISC
e9
e10
e11
e12
e13
BIENS
e14
e15
e17
e18

Variable counts (Group number 1)

Number of variables in your model: 37
Number of observed variables: 17
Number of unobserved variables: 20
Number of exogenous variables: 20
Number of endogenous variables: 17

Parameter Summary (Group number 1)

	Weights	Covariances	Variances	Means	Intercepts	Total
Fixed	20	0	0	0	0	20
Labeled	0	0	0	0	0	0
Unlabeled	14	5	20	0	0	39
Total	34	5	20	0	0	59

Models

Default model (Default model)

Notes for Model (Default model)

Computation of degrees of freedom (Default model)

Number of distinct sample moments: 153

Number of distinct parameters to be estimated: 39
Degrees of freedom (153 — 39): 114

Result (Default model)

Minimum was achieved
Chi—square = 997.891
Degrees of freedom = 114
Probability level = 0.000

Group number 1 (Group number 1 — Default model)

Estimates (Group number 1 — Default model)

Scalar Estimates (Group number 1 — Default model)

Maximum Likelihood Estimates

Regression Weights: (Group number 1 — Default model)

			Estimate	S.E.	C.R.	*p*	Label
MESNR	<———	MALEST	1.000				
MESP	<———	MALEST	1.029	.021	48.904	***	
MESZZ	<———	MALEST	1.038	.032	32.697	***	
MESTN	<———	MALEST	1.017	.031	32.297	***	
MESTRI	<———	MALEST	1.058	.033	32.489	***	
MESIR	<———	MALEST	1.056	.034	30.832	***	
MESDI	<———	MALEST	.970	.033	29.203	***	
PERIN	<———	MALEST	.973	.034	28.822	***	
MPROS	<———	FISC	1.000				
MDOLMU	<———	FISC	1.177	.043	27.609	***	
MCABE	<———	FISC	1.146	.042	27.345	***	
MCOME	<———	FISC	1.148	.043	26.578	***	
MDIGE	<———	FISC	1.129	.043	26.272	***	
MESTR	<———	BIENS	1.000				
MALEG	<———	BIENS	1.309	.053	24.554	***	
MILUSI	<———	BIENS	1.253	.054	23.376	***	
MEVIT	<———	BIENS	1.234	.053	23.095	***	

Standardized Regression Weights: (Group number 1 — Default model)

			Estimate
MESNR	<———	MALEST	.806
MESP	<———	MALEST	.842
MESZZ	<———	MALEST	.818
MESTN	<———	MALEST	.811
MESTRI	<———	MALEST	.814
MESIR	<———	MALEST	.783
MESDI	<———	MALEST	.753
PERIN	<———	MALEST	.745
MPROS	<———	FISC	.735
MDOLMU	<———	FISC	.806
MCABE	<———	FISC	.798
MCOME	<———	FISC	.777
MDIGE	<———	FISC	.768
MESTR	<———	BIENS	.678
MALEG	<———	BIENS	.845
MILUSI	<———	BIENS	.782
MEVIT	<———	BIENS	.769

Covariances: (Group number 1 — Default model)

			Estimate	S.E.	C.R.	*p*	Label
MALEST	<--->	FISC	.703	.042	16.946	***	
MALEST	<--->	BIENS	.169	.024	6.918	***	
FISC	<--->	BIENS	.198	.024	8.262	***	
e1	<--->	e2	.237	.019	12.714	***	
e7	<--->	e8	.428	.027	16.043	***	

Correlations: (Group number 1 — Default model)

			Estimate
MALEST	<--->	FISC	.793
MALEST	<--->	BIENS	.234
FISC	<--->	BIENS	.296
e1	<--->	e2	.511
e7	<--->	e8	.604

Variances: (Group number 1 — Default model)

	Estimate	S.E.	C.R.	*p*	Label
MALEST	.959	.057	16.755	***	
FISC	.821	.057	14.473	***	
BIENS	.548	.043	12.680	***	
e1	.516	.025	20.985	***	
e2	.418	.021	20.019	***	
e3	.511	.024	20.954	***	
e4	.517	.024	21.156	***	
e5	.546	.026	21.061	***	
e6	.673	.031	21.789	***	
e7	.691	.031	22.278	***	
e8	.727	.033	22.379	***	
e9	.701	.032	21.604	***	
e10	.614	.031	19.710	***	
e11	.614	.031	19.975	***	
e12	.712	.035	20.640	***	
e13	.729	.035	20.868	***	
e14	.645	.030	21.429	***	
e15	.376	.026	14.637	***	
e17	.548	.030	18.339	***	
e18	.575	.030	18.865	***	

Matrices (Group number 1 — Default model)

Total Effects (Group number 1 — Default model)

	BIENS	FISC	MALEST
MEVIT	1.234	.000	.000
MILUSI	1.253	.000	.000
MALEG	1.309	.000	.000
MESTR	1.000	.000	.000
MDIGE	.000	1.129	.000
MCOME	.000	1.148	.000
MCABE	.000	1.146	.000
MDOLMU	.000	1.177	.000
MPROS	.000	1.000	.000
PERIN	.000	.000	.973
MESDI	.000	.000	.970
MESIR	.000	.000	1.056
MESTRI	.000	.000	1.058
MESTN	.000	.000	1.017
MESZZ	.000	.000	1.038
MESP	.000	.000	1.029
MESNR	.000	.000	1.000

Standardized Total Effects (Group number 1 — Default model)

	BIENS	FISC	MALEST
MEVIT	.769	.000	.000
MILUSI	.782	.000	.000
MALEG	.845	.000	.000
MESTR	.678	.000	.000
MDIGE	.000	.768	.000
MCOME	.000	.777	.000
MCABE	.000	.798	.000
MDOLMU	.000	.806	.000
MPROS	.000	.735	.000
PERIN	.000	.000	.745
MESDI	.000	.000	.753
MESIR	.000	.000	.783
MESTRI	.000	.000	.814
MESTN	.000	.000	.811
MESZZ	.000	.000	.818
MESP	.000	.000	.842
MESNR	.000	.000	.806

Direct Effects (Group number 1 — Default model)

	BIENS	FISC	MALEST
MEVIT	1.234	.000	.000
MILUSI	1.253	.000	.000
MALEG	1.309	.000	.000
MESTR	1.000	.000	.000
MDIGE	.000	1.129	.000
MCOME	.000	1.148	.000
MCABE	.000	1.146	.000
MDOLMU	.000	1.177	.000
MPROS	.000	1.000	.000
PERIN	.000	.000	.973
MESDI	.000	.000	.970
MESIR	.000	.000	1.056
MESTRI	.000	.000	1.058
MESTN	.000	.000	1.017
MESZZ	.000	.000	1.038
MESP	.000	.000	1.029
MESNR	.000	.000	1.000

Standardized Direct Effects (Group number 1 — Default model)

	BIENS	FISC	MALEST
MEVIT	.769	.000	.000
MILUSI	.782	.000	.000
MALEG	.845	.000	.000
MESTR	.678	.000	.000
MDIGE	.000	.768	.000
MCOME	.000	.777	.000
MCABE	.000	.798	.000
MDOLMU	.000	.806	.000
MPROS	.000	.735	.000
PERIN	.000	.000	.745
MESDI	.000	.000	.753
MESIR	.000	.000	.783
MESTRI	.000	.000	.814
MESTN	.000	.000	.811
MESZZ	.000	.000	.818
MESP	.000	.000	.842
MESNR	.000	.000	.806

			M.I.	Par Change
e17	<——>	FISC	9.303	.048
e14	<——>	e15	7.572	.049
e13	<——>	BIENS	6.932	−.053
e13	<——>	FISC	6.426	.043
e13	<——>	MALEST	6.424	−.047
e13	<——>	e15	4.467	−.042
e12	<——>	FISC	9.865	−.053
e12	<——>	MALEST	12.358	.064
e12	<——>	e17	14.125	.082
e11	<——>	e18	19.786	−.092
e11	<——>	e15	7.293	.050
e10	<——>	e18	5.518	−.049
e10	<——>	e15	4.490	.039
e10	<——>	e13	18.856	−.097
e10	<——>	e11	39.278	.130
e9	<——>	e18	33.333	.124
e9	<——>	e15	14.771	−.073
e9	<——>	e13	83.593	.211
e9	<——>	e12	9.276	−.070
e9	<——>	e11	18.251	−.092
e8	<——>	FISC	28.283	.069
e8	<——>	MALEST	15.806	−.055
e8	<——>	e17	8.843	.048
e8	<——>	e10	13.890	.063
e7	<——>	e13	4.636	.038
e7	<——>	e9	13.056	.061
e6	<——>	FISC	38.699	.102
e6	<——>	MALEST	25.002	−.087
e6	<——>	e18	12.125	.073
e6	<——>	e14	7.309	−.057
e6	<——>	e11	5.579	.050
e6	<——>	e8	16.225	.069
e6	<——>	e7	11.053	.055
e5	<——>	FISC	18.909	.065
e5	<——>	MALEST	9.510	−.049
e5	<——>	e18	10.689	−.063
e5	<——>	e9	13.861	.075
e5	<——>	e8	8.603	.046
e5	<——>	e6	7.711	.054
e4	<——>	FISC	5.933	−.035
e4	<——>	e14	9.821	.059
e4	<——>	e10	11.368	−.064
e4	<——>	e7	7.673	−.041
e4	<——>	e6	8.242	−.055
e4	<——>	e5	5.163	.039
e3	<——>	FISC	64.109	−.116
e3	<——>	MALEST	37.949	.095
e3	<——>	e18	4.039	.037
e3	<——>	e13	8.210	−.058
e3	<——>	e9	7.613	−.054
e3	<——>	e8	25.176	−.076
e3	<——>	e6	42.669	−.124
e3	<——>	e5	27.214	−.090
e2	<——>	FISC	11.453	−.037
e2	<——>	MALEST	5.866	.028
e2	<——>	e18	5.978	.034
e2	<——>	e13	5.417	−.035

			M.I.	Par Change
e2	<——>	e9	5.511	−.034
e2	<——>	e7	7.526	−.030
e2	<——>	e6	5.376	−.033
e2	<——>	e5	18.189	−.055
e2	<——>	e3	129.956	.144
e1	<——>	FISC	4.079	−.024
e1	<——>	e8	9.709	−.038
e1	<——>	e6	8.067	−.044
e1	<——>	e4	8.203	.039
e1	<——>	e3	19.699	.061

Variances: (Group number 1 — Default model)

	M.I.	Par Change

Regression Weights: (Group number 1 — Default model)

			M.I.	Par Change
MEVIT	<———	MCABE	5.753	−.044
MEVIT	<———	MPROS	13.847	.073
MEVIT	<———	MESIR	6.503	.047
MILUSI	<———	FISC	18.798	.120
MILUSI	<———	MALEST	12.616	.089
MILUSI	<———	MDIGE	9.006	.053
MILUSI	<———	MCOME	28.969	.095
MILUSI	<———	MCABE	6.349	.046
MILUSI	<———	MDOLMU	12.980	.065
MILUSI	<———	MPROS	14.432	.073
MILUSI	<———	PERIN	24.347	.092
MILUSI	<———	MESDI	15.322	.074
MILUSI	<———	MESIR	6.248	.045
MILUSI	<———	MESTRI	14.009	.070
MILUSI	<———	MESTN	9.116	.058
MILUSI	<———	MESZZ	4.404	.040
MALEG	<———	FISC	9.360	−.076
MALEG	<———	MALEST	11.841	−.078
MALEG	<———	MDIGE	12.154	−.056
MALEG	<———	MCOME	6.637	−.041
MALEG	<———	MPROS	21.050	−.080
MALEG	<———	PERIN	17.085	−.069
MALEG	<———	MESDI	12.780	−.061
MALEG	<———	MESIR	7.341	−.044
MALEG	<———	MESTRI	5.780	−.041
MALEG	<———	MESTN	8.366	−.050
MALEG	<———	MESZZ	10.739	−.057
MALEG	<———	MESP	6.075	−.044
MALEG	<———	MESNR	4.172	−.036
MDIGE	<———	BIENS	6.178	−.095
MDIGE	<———	MALEG	8.273	−.066
MDIGE	<———	MESTR	6.670	−.062
MDIGE	<———	MDOLMU	5.605	−.047
MDIGE	<———	MPROS	34.789	.126
MDIGE	<———	MESZZ	6.765	−.055
MDIGE	<———	MESP	6.981	−.058
MDIGE	<———	MESNR	4.495	−.046
MCOME	<———	MILUSI	11.097	.073
MCOME	<———	PERIN	9.531	.063
MCOME	<———	MESDI	7.335	.056

			M.I.	Par Change
MCOME	<———	MESTRI	4.810	.045
MCABE	<———	MEVIT	13.968	−.077
MCABE	<———	MDOLMU	11.786	.064
MCABE	<———	MPROS	7.638	−.055
MDOLMU	<———	MALEG	4.143	.044
MDOLMU	<———	MDIGE	6.889	−.049
MDOLMU	<———	MCABE	12.334	.067
MDOLMU	<———	MESTN	6.045	−.049
MPROS	<———	MEVIT	16.500	.087
MPROS	<———	MDIGE	30.096	.104
MPROS	<———	MCABE	5.626	−.046
MPROS	<———	PERIN	5.449	.046
MPROS	<———	MESDI	11.141	.067
MPROS	<———	MESTRI	4.826	.044
PERIN	<———	FISC	7.343	.062
PERIN	<———	MCOME	9.029	.044
PERIN	<———	MCABE	5.635	.036
PERIN	<———	MDOLMU	16.567	.060
PERIN	<———	MPROS	4.679	.034
PERIN	<———	MESIR	5.579	.035
PERIN	<———	MESZZ	7.166	−.042
PERIN	<———	MESNR	5.252	−.037
MESDI	<———	MPROS	8.081	.044
MESIR	<———	FISC	11.855	.099
MESIR	<———	MEVIT	11.057	.070
MESIR	<———	MDIGE	11.138	.062
MESIR	<———	MCOME	8.398	.054
MESIR	<———	MCABE	15.254	.074
MESIR	<———	MDOLMU	12.810	.067
MESIR	<———	MPROS	10.295	.065
MESIR	<———	PERIN	25.070	.097
MESIR	<———	MESDI	22.222	.093
MESIR	<———	MESZZ	12.357	−.070
MESIR	<———	MESP	5.335	−.048
MESIR	<———	MESNR	7.385	−.056
MESTRI	<———	FISC	4.529	.056
MESTRI	<———	MEVIT	7.342	−.052
MESTRI	<———	MCOME	5.445	.040
MESTRI	<———	MCABE	5.351	.040
MESTRI	<———	MPROS	14.918	.071
MESTRI	<———	PERIN	7.079	.047
MESTRI	<———	MESZZ	7.926	−.051
MESTRI	<———	MESP	7.696	−.053
MESTN	<———	MESTR	8.928	.060
MESTN	<———	MDOLMU	7.090	−.044
MESTN	<———	PERIN	6.137	−.043
MESTN	<———	MESDI	8.508	−.051
MESTN	<———	MESNR	5.149	.041
MESZZ	<———	FISC	18.113	−.109
MESZZ	<———	MDIGE	23.104	−.079
MESZZ	<———	MCOME	14.079	−.062
MESZZ	<———	MCABE	17.097	−.070
MESZZ	<———	MDOLMU	16.454	−.067
MESZZ	<———	MPROS	22.390	−.084
MESZZ	<———	PERIN	14.365	−.065
MESZZ	<———	MESIR	14.941	−.064
MESZZ	<———	MESTRI	8.111	−.049

			M.I.	Par Change
MESZZ	<———	MESP	69.763	.154
MESZZ	<———	MESNR	47.630	.125
MESP	<———	MDIGE	6.718	−.032
MESP	<———	MPROS	7.054	−.035
MESP	<———	MESDI	6.274	−.033
MESP	<———	MESTRI	5.345	−.030
MESP	<———	MESZZ	37.330	.081
MESNR	<———	PERIN	4.720	−.030
MESNR	<———	MESZZ	5.617	.034

Minimization History (Default model)

Iteration		Negative eigenvalues	Condition #	Smallest eigenvalue	Diameter	F	NTries	Ratio
0	e	8		−1.530	9999.000	14442.102	0	9999.000
1	e*	10		−.732	2.159	8625.151	19	.389
2	e	7		−.411	.575	6569.992	6	.999
3	e	5		−.542	.387	5654.241	4	.682
4	e*	1		−.034	.947	2894.449	6	.901
5	e*	0	503.787		1.186	1493.281	5	.676
6	e	0	119.253		1.140	1196.234	1	.732
7	e	0	193.535		.318	1013.328	1	1.131
8	e	0	243.093		.133	998.593	1	1.116
9	e	0	254.885		.043	997.895	1	1.043
10	e	0	260.023		.003	997.891	1	1.004
11	e	0	259.461		.000	997.891	1	1.000

Model Fit Summary

CMIN

Model	NPAR	CMIN	*df*	*p*	CMIN/DF
Default model	39	997.891	114	.000	8.753
Saturated model	153	.000	0		
Independence model	17	14938.900	136	.000	109.845

RMR. GFI

Model	RMR	GFI	AGFI	PGFI
Default model	.072	.902	.869	.672
Saturated model	.000	1.000		
Independence model	.694	.226	.129	.201

Baseline Comparisons

Model	NFI Delta1	RFI rho1	IFI Delta2	TLI rho2	CFI
Default model	.933	.920	.940	.929	.940
Saturated model	1.000		1.000		1.000
Independence model	.000	.000	.000	.000	.000

Parsimony—Adjusted Measures

Model	PRATIO	PNFI	PCFI
Default model	.838	.782	.788
Saturated model	.000	.000	.000
Independence model	1.000	.000	.000

NCP

Model	NCP	LO 90	HI 90
Default model	883.891	786.673	988.563
Saturated model	.000	.000	.000

Model	NCP	LO 90	HI 90
Independence model	14802.900	14404.886	15207.208

FMIN

Model	FMIN	F0	LO 90	HI 90
Default model	.811	.718	.639	.803
Saturated model	.000	.000	.000	.000
Independence model	12.136	12.025	11.702	12.354

RMSEA

Model	RMSEA	LO 90	HI 90	PCLOSE
Default model	.079	.075	.084	.000
Independence model	.297	.293	.301	.000

AIC

Model	AIC	BCC	BIC	CAIC
Default model	1075.891	1077.049	1275.431	1314.431
Saturated model	306.000	310.541	1088.808	1241.808
Independence model	14972.900	14973.404	15059.878	15076.878

ECVI

Model	ECVI	LO 90	HI 90	MECVI
Default model	.874	.795	.959	.875
Saturated model	.249	.249	.249	.252
Independence model	12.163	11.840	12.492	12.164

HOELTER

Model	HOELTER .05	HOELTER .01
Default model	173	188
Independence model	14	15

Execution time summary

Minimization: .012
Miscellaneous: .594
Bootstrap: .000
Total: .606

Path Diagram: Confirmatory Factor Analysis

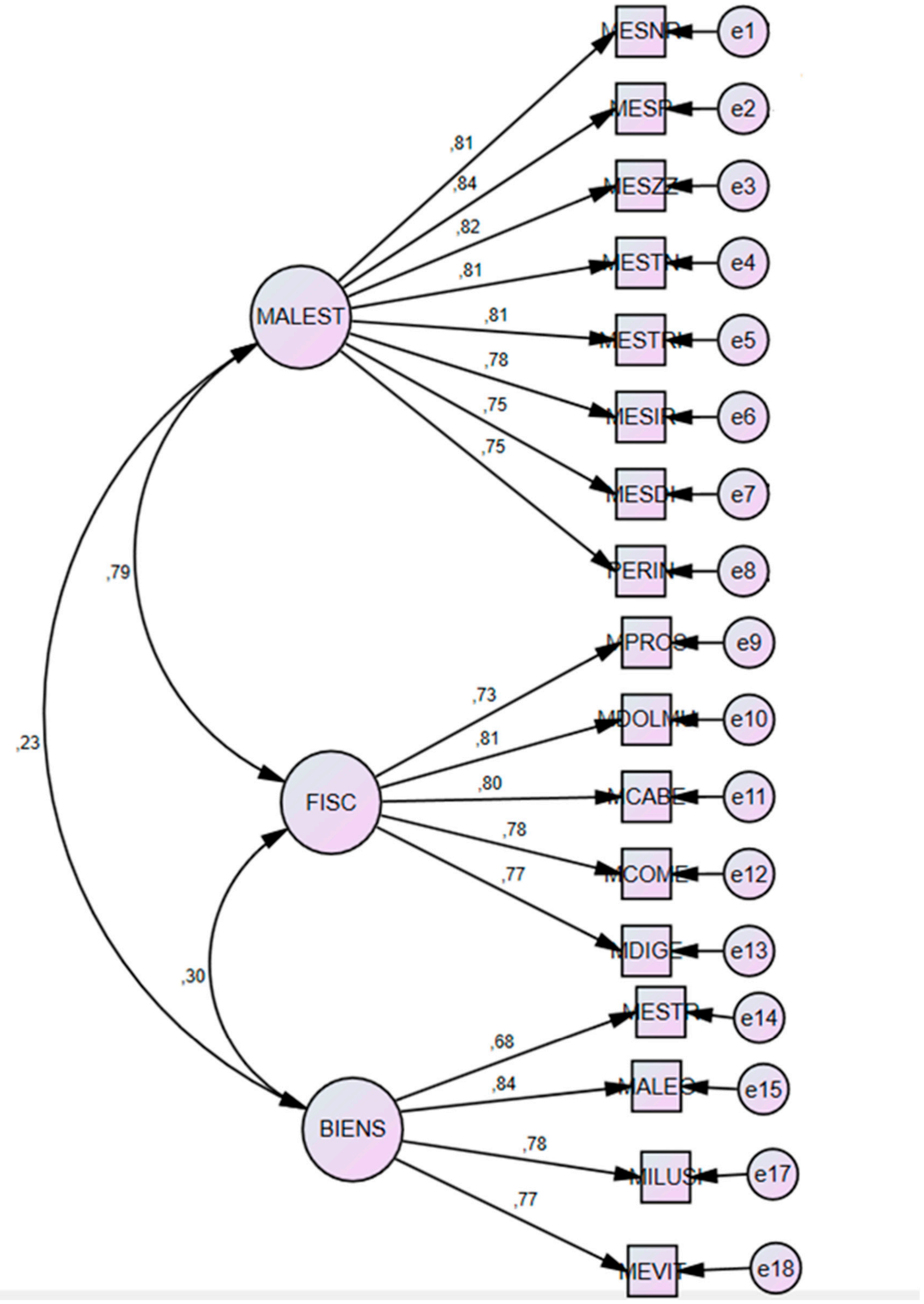

MALEST
FISC
BIENS
,81
,84
,82
,81
,81
,78
,75
,75
,79
,23
,73
,81
,80
,78
,77
,30
,68
,84
,78
,77
e1
e2
e3
e4
e5
e6
e7
e8
e9
e10
e11
e12
e13
e14
e15
e17
e18

C:\Users\media\Dropbox\Mi PC (LAPTOP－LCRG33R5)\Desktop\2024\Articulos 2024\Salud mental 2024 1\Analisis adicional noviembre\AFC TOTAL － copia\AFCTOTAL.amw

Analysis Summary

Date and Time

Date: martes. 26 de noviembre de 2024
Time: 5:59:20 p. m.

Title

Afctotal: martes. 26 de noviembre de 2024 5:59 p. m.

Groups

Group number 1 (Group number 1)

Notes for Group (Group number 1)

The model is recursive.
Sample size = 1232

Variable Summary (Group number 1)

Your model contains the following variables (Group number 1)

Observed. endogenous variables
APOYCOM
APOYBIE
APOYDOC
APOTTRAB
APOYCOMP
APOYAMIG
APOYFAM
APOYPAR
AMIGOS
COMFAM
GRUPOS
RECREA
CULTUR
DEPORT
DORMIR
COMER
FORTALZ
EMPATI
EXPRSN
INTERACT
DECISNS
AFRONT
INNOVA
CUESTIN
EXPRESEMOC
INDENTIF
ENTHG
RELHOG
CONFLHOG
SOLHGR
ENTTBA
RELIBAR
CONFBARR
SOLBAR
ENTUNI
RELUNIVE
CONFCUNI
SOLUNIV
ENTTRAB
RELIOTRB
CONFTRA
SOLTRAB

ENTREL
RELREL
CONFREL
SOLREL
ENTHOB
RELHOB
CONFHOB
SOLHOB
MALESSLD
MALFINAZ
MALSOCL
MALFAM
MALPAR
DAÑO
INTENT
IDEACI
COMNERGZ
COMMDIC
CONSDRG
CONTABAC
CONSALCH
PROLGLS
PROBACDM
PRMLABR
PROMECON
CONFAMIG
CONFPRJ
CONFFAM
RESDSLS
APUESTAS
PORN
VIDEOJG
CFLEGAL
CFACADM
CFLABR
CFECNM
CFAMIGS
CFPAREJA
CFFAMILIA
NERVS
PRCUPC
ZOZ
TENSINE
TRIT
IRRIT
NODISFR
PERDDNT
PROBLDI
COMRM
DOLCA
DOLRMU
PROBS
OPTIM
VITALI
ALGR
TRAQUIL
MESNR
MESP
MESZZ
MESTN
MESTRI
MESIR
MESDI
PERIN
MPROS
MDOLMU
MCABE
MCOME
MDIGE
MALEG
MILUSI
MEVIT
MESTR

Unobserved. exogenous variables
APOY
e1
e2
e3
e4
e5
e6
e7
e8
HAB
e9
e10
e11
e12
e13
e14
e15
e16
HP
e17
e18
e19
e20
e21
e22
e23
e24
e25
e26
HOG
e27
e28
e29
e30
BAR
e31
e32
e33
e34
ESC
e35
e36
e37
e38
LAB
e39
e40
e41
e42
REL
e43
e44
e45
e46
CUL
e47
e48
e49
e50
MALS
e51
e52
e53
e54
e55
SUI
e56
e57
e58
DRUG
e59
e60

e61
e62
e63
PDRUG
e64
e65
e66
e67
e68
e69
e70
USOINT
e71
e72
e73
e74
PROINT
e75
e76
e77
e78
e79
e80
e81
SESEM
e82
e83
e84
e85
e86
e87
e88
e89
SFUSEM
e90
e91
e92
e93
e94
BIMS
e95
e96
e97
e98
SEMES
e99
e100
e101
e102
e103
e104
e105
e106
SFMES
e107
e108
e109
e110
e111
BIEMS
e112
e113
e114
e115

Variable counts (Group number 1)

Number of variables in your model:	251
Number of observed variables:	115
Number of unobserved variables:	136
Number of exogenous variables:	136
Number of endogenous variables:	115

Parameter Summary (Group number 1)

	Weights	Covariances	Variances	Means	Intercepts	Total
Fixed	136	0	0	0	0	136
Labeled	0	0	0	0	0	0
Unlabeled	94	229	136	0	0	459
Total	230	229	136	0	0	595

Models

Default model (Default model)

Notes for Model (Default model)

Computation of degrees of freedom (Default model)

Number of distinct sample moments:	6670
Number of distinct parameters to be estimated:	459
Degrees of freedom (6670 — 459):	6211

Result (Default model)

Minimum was achieved
Chi—square = 19642.116
Degrees of freedom = 6211
Probability level = 0.000

Group number 1 (Group number 1 — Default model)

Estimates (Group number 1 — Default model)

Scalar Estimates (Group number 1 — Default model)

Maximum Likelihood Estimates

Regression Weights: (Group number 1 — Default model)

			Estimate	S.E.	C.R.	*p*	Label
APOYCOM	<———	APOY	1.000				
APOYBIE	<———	APOY	1.135	.053	21.535	***	
APOYDOC	<———	APOY	1.162	.052	22.549	***	
APOTTRAB	<———	APOY	.505	.051	9.940	***	
APOYCOMP	<———	APOY	.731	.045	16.413	***	
APOYAMIG	<———	APOY	.377	.040	9.523	***	
APOYFAM	<———	APOY	.258	.028	9.073	***	
APOYPAR	<———	APOY	.122	.064	1.907	.056	
AMIGOS	<———	HAB	1.000				
COMFAM	<———	HAB	.850	.066	12.951	***	
GRUPOS	<———	HAB	.983	.085	11.528	***	
RECREA	<———	HAB	1.651	.114	14.446	***	
CULTUR	<———	HAB	1.325	.093	14.227	***	
DEPORT	<———	HAB	1.197	.105	11.411	***	
DORMIR	<———	HAB	.280	.065	4.322	***	
COMER	<———	HAB	.498	.072	6.870	***	
FORTALZ	<———	HP	1.000				
EMPATI	<———	HP	.667	.044	15.083	***	
EXPRSN	<———	HP	1.143	.057	20.119	***	
INTERACT	<———	HP	1.056	.052	20.326	***	
DECISNS	<———	HP	1.062	.051	21.022	***	
AFRONT	<———	HP	1.212	.056	21.548	***	
INNOVA	<———	HP	1.233	.057	21.673	***	
CUESTIN	<———	HP	1.077	.054	19.954	***	
EXPRESEMOC	<———	HP	1.207	.058	20.643	***	

			Estimate	S.E.	C.R.	*p*	Label
INDENTIF	<---	HP	1.201	.057	20.921	***	
ENTHG	<---	HOG	1.000				
RELHOG	<---	HOG	.838	.048	17.506	***	
CONFLHOG	<---	HOG	.771	.048	16.193	***	
SOLHGR	<---	HOG	.849	.055	15.319	***	
ENTTBA	<---	BAR	1.000				
RELIBAR	<---	BAR	2.419	.200	12.096	***	
CONFBARR	<---	BAR	1.209	.123	9.794	***	
SOLBAR	<---	BAR	1.725	.157	10.993	***	
ENTUNI	<---	ESC	1.000				
RELUNIVE	<---	ESC	.757	.078	9.713	***	
CONFCUNI	<---	ESC	.667	.084	7.986	***	
SOLUNIV	<---	ESC	.942	.116	8.124	***	
ENTTRAB	<---	LAB	1.000				
RELIOTRB	<---	LAB	1.096	.020	53.864	***	
CONFTRA	<---	LAB	.785	.018	44.507	***	
SOLTRAB	<---	LAB	.706	.020	35.297	***	
ENTREL	<---	REL	1.000				
RELREL	<---	REL	1.085	.027	40.547	***	
CONFREL	<---	REL	.769	.024	32.050	***	
SOLREL	<---	REL	.553	.020	27.182	***	
ENTHOB	<---	CUL	1.000				
RELHOB	<---	CUL	1.031	.018	58.809	***	
CONFHOB	<---	CUL	.732	.018	40.269	***	
SOLHOB	<---	CUL	.565	.018	31.561	***	
MALESSLD	<---	MALS	1.000				
MALFINAZ	<---	MALS	1.084	.076	14.206	***	
MALSOCL	<---	MALS	.868	.065	13.403	***	
MALFAM	<---	MALS	1.340	.086	15.531	***	
MALPAR	<---	MALS	.585	.071	8.267	***	
DAÑO	<---	SUI	1.000				
INTENT	<---	SUI	.844	.068	12.372	***	
IDEACI	<---	SUI	1.285	.090	14.280	***	
COMNERGZ	<---	DRUG	1.000				
COMMDIC	<---	DRUG	.348	.039	8.935	***	
CONSDRG	<---	DRUG	.668	.055	12.135	***	
CONTABAC	<---	DRUG	.631	.055	11.559	***	
CONSALCH	<---	DRUG	1.143	.086	13.272	***	
PROLGLS	<---	PDRUG	1.000				
PROBACDM	<---	PDRUG	1.230	.028	43.694	***	
PRMLABR	<---	PDRUG	.919	.027	33.916	***	
PROMECON	<---	PDRUG	1.210	.029	42.097	***	
CONFAMIG	<---	PDRUG	1.187	.028	41.663	***	
CONFPRJ	<---	PDRUG	.935	.033	28.133	***	
CONFFAM	<---	PDRUG	1.278	.032	39.526	***	
RESDSLS	<---	USOINT	1.000				
APUESTAS	<---	USOINT	2.859	.956	2.989	.003	
PORN	<---	USOINT	6.919	2.263	3.058	.002	
VIDEOJG	<---	USOINT	9.845	3.217	3.061	.002	
CFLEGAL	<---	PROINT	1.000				
CFACADM	<---	PROINT	1.284	.059	21.645	***	
CFLABR	<---	PROINT	1.061	.046	22.848	***	
CFECNM	<---	PROINT	1.317	.047	28.250	***	
CFAMIGS	<---	PROINT	1.256	.044	28.245	***	
CFPAREJA	<---	PROINT	1.054	.055	19.330	***	
CFFAMILIA	<---	PROINT	1.271	.059	21.645	***	
NERVS	<---	SESEM	1.000				

			Estimate	S.E.	C.R.	*p*	Label
PRCUPC	<---	SESEM	1.030	.031	32.928	***	
ZOZ	<---	SESEM	1.121	.046	24.508	***	
TENSINE	<---	SESEM	1.130	.044	25.456	***	
TRIT	<---	SESEM	1.168	.046	25.133	***	
IRRIT	<---	SESEM	1.158	.048	24.204	***	
NODISFR	<---	SESEM	.984	.045	22.109	***	
PERDDNT	<---	SESEM	1.010	.046	22.157	***	
PROBLDI	<---	SFUSEM	1.000				
COMRM	<---	SFUSEM	.998	.046	21.681	***	
DOLCA	<---	SFUSEM	1.086	.046	23.510	***	
DOLRMU	<---	SFUSEM	1.042	.046	22.544	***	
PROBS	<---	SFUSEM	.853	.041	20.739	***	
OPTIM	<---	BIMS	1.000				
VITALI	<---	BIMS	1.096	.052	21.035	***	
ALGR	<---	BIMS	1.122	.050	22.330	***	
TRAQUIL	<---	BIMS	.833	.046	18.166	***	
MESNR	<---	SEMES	1.000				
MESP	<---	SEMES	1.021	.021	49.669	***	
MESZZ	<---	SEMES	1.034	.031	33.887	***	
MESTN	<---	SEMES	1.009	.030	33.289	***	
MESTRI	<---	SEMES	1.046	.031	33.295	***	
MESIR	<---	SEMES	1.037	.032	32.168	***	
MESDI	<---	SEMES	.957	.032	29.753	***	
PERIN	<---	SEMES	.959	.033	29.296	***	
MPROS	<---	SFMES	1.000				
MDOLMU	<---	SFMES	1.183	.042	28.024	***	
MCABE	<---	SFMES	1.151	.042	27.727	***	
MCOME	<---	SFMES	1.156	.043	27.014	***	
MDIGE	<---	SFMES	1.143	.043	26.841	***	
MALEG	<---	BIEMS	1.000				
MILUSI	<---	BIEMS	.984	.033	30.056	***	
MEVIT	<---	BIEMS	.977	.033	29.800	***	
MESTR	<---	BIEMS	.789	.031	25.280	***	

Standardized Regression Weights: (Group number 1 — Default model)

			Estimate
APOYCOM	<---	APOY	.693
APOYBIE	<---	APOY	.735
APOYDOC	<---	APOY	.794
APOTTRAB	<---	APOY	.316
APOYCOMP	<---	APOY	.535
APOYAMIG	<---	APOY	.304
APOYFAM	<---	APOY	.288
APOYPAR	<---	APOY	.060
AMIGOS	<---	HAB	.436
COMFAM	<---	HAB	.382
GRUPOS	<---	HAB	.476
RECREA	<---	HAB	.844
CULTUR	<---	HAB	.773
DEPORT	<---	HAB	.467
DORMIR	<---	HAB	.139
COMER	<---	HAB	.232
FORTALZ	<---	HP	.631
EMPATI	<---	HP	.482
EXPRSN	<---	HP	.679
INTERACT	<---	HP	.688
DECISNS	<---	HP	.718

			Estimate
AFRONT	<---	HP	.745
INNOVA	<---	HP	.751
CUESTIN	<---	HP	.672
EXPRESEMOC	<---	HP	.702
INDENTIF	<---	HP	.714
ENTHG	<---	HOG	.691
RELHOG	<---	HOG	.657
CONFLHOG	<---	HOG	.586
SOLHGR	<---	HOG	.546
ENTTBA	<---	BAR	.458
RELIBAR	<---	BAR	.704
CONFBARR	<---	BAR	.410
SOLBAR	<---	BAR	.507
ENTUNI	<---	ESC	.534
RELUNIVE	<---	ESC	.474
CONFCUNI	<---	ESC	.335
SOLUNIV	<---	ESC	.330
ENTTRAB	<---	LAB	.892
RELIOTRB	<---	LAB	.951
CONFTRA	<---	LAB	.862
SOLTRAB	<---	LAB	.759
ENTREL	<---	REL	.825
RELREL	<---	REL	.939
CONFREL	<---	REL	.764
SOLREL	<---	REL	.643
ENTHOB	<---	CUL	.910
RELHOB	<---	CUL	.957
CONFHOB	<---	CUL	.811
SOLHOB	<---	CUL	.666
MALESSLD	<---	MALS	.552
MALFINAZ	<---	MALS	.589
MALSOCL	<---	MALS	.536
MALFAM	<---	MALS	.714
MALPAR	<---	MALS	.289
DAÑO	<---	SUI	.611
INTENT	<---	SUI	.495
IDEACI	<---	SUI	.677
COMNERGZ	<---	DRUG	.490
COMMDIC	<---	DRUG	.342
CONSDRG	<---	DRUG	.546
CONTABAC	<---	DRUG	.500
CONSALCH	<---	DRUG	.681
PROLGLS	<---	PDRUG	.858
PROBACDM	<---	PDRUG	.899
PRMLABR	<---	PDRUG	.741
PROMECON	<---	PDRUG	.882
CONFAMIG	<---	PDRUG	.877
CONFPRJ	<---	PDRUG	.631
CONFFAM	<---	PDRUG	.852
RESDSLS	<---	USOINT	.110
APUESTAS	<---	USOINT	.373
PORN	<---	USOINT	.588
VIDEOJG	<---	USOINT	.649
CFLEGAL	<---	PROINT	.728
CFACADM	<---	PROINT	.642
CFLABR	<---	PROINT	.631
CFECNM	<---	PROINT	.836

			Estimate
CFAMIGS	<---	PROINT	.836
CFPAREJA	<---	PROINT	.515
CFFAMILIA	<---	PROINT	.642
NERVS	<---	SESEM	.691
PRCUPC	<---	SESEM	.761
ZOZ	<---	SESEM	.759
TENSINE	<---	SESEM	.791
TRIT	<---	SESEM	.780
IRRIT	<---	SESEM	.729
NODISFR	<---	SESEM	.679
PERDDNT	<---	SESEM	.681
PROBLDI	<---	SFUSEM	.680
COMRM	<---	SFUSEM	.682
DOLCA	<---	SFUSEM	.747
DOLRMU	<---	SFUSEM	.712
PROBS	<---	SFUSEM	.650
OPTIM	<---	BIMS	.661
VITALI	<---	BIMS	.726
ALGR	<---	BIMS	.793
TRAQUIL	<---	BIMS	.606
MESNR	<---	SEMES	.815
MESP	<---	SEMES	.844
MESZZ	<---	SEMES	.823
MESTN	<---	SEMES	.813
MESTRI	<---	SEMES	.813
MESIR	<---	SEMES	.777
MESDI	<---	SEMES	.751
PERIN	<---	SEMES	.742
MPROS	<---	SFMES	.730
MDOLMU	<---	SFMES	.805
MCABE	<---	SFMES	.797
MCOME	<---	SFMES	.778
MDIGE	<---	SFMES	.773
MALEG	<---	BIEMS	.827
MILUSI	<---	BIEMS	.786
MEVIT	<---	BIEMS	.780
MESTR	<---	BIEMS	.684

Model Fit Summary

CMIN

Model	NPAR	CMIN	*df*	*p*	CMIN/DF
Default model	459	19642.116	6211	.000	3.162
Saturated model	6670	.000	0		
Independence model	115	85320.715	6555	.000	13.016

RMR. GFI

Model	RMR	GFI	AGFI	PGFI
Default model	.108	.771	.754	.718
Saturated model	.000	1.000		
Independence model	.364	.231	.217	.227

Baseline Comparisons

Model	NFI Delta1	RFI rho1	IFI Delta2	TLI rho2	CFI
Default model	.770	.757	.830	.820	.829
Saturated model	1.000		1.000		1.000

Model	NFI Delta1	RFI rho1	IFI Delta2	TLI rho2	CFI
Independence model	.000	.000	.000	.000	.000

Parsimony－Adjusted Measures

Model	PRATIO	PNFI	PCFI
Default model	.948	.729	.786
Saturated model	.000	.000	.000
Independence model	1.000	.000	.000

NCP

Model	NCP	LO 90	HI 90
Default model	13431.116	13011.579	13857.645
Saturated model	.000	.000	.000
Independence model	78765.715	77826.666	79711.213

FMIN

Model	FMIN	F0	LO 90	HI 90
Default model	15.956	10.911	10.570	11.257
Saturated model	.000	.000	.000	.000
Independence model	69.310	63.985	63.222	64.753

RMSEA

Model	RMSEA	LO 90	HI 90	PCLOSE
Default model	.042	.041	.043	1.000
Independence model	.099	.098	.099	.000

AIC

Model	AIC	BCC	BIC	CAIC
Default model	20560.116	20655.621	22908.541	23367.541
Saturated model	13340.000	14727.839	47466.349	54136.349
Independence model	85550.715	85574.643	86139.100	86254.100

ECVI

Model	ECVI	LO 90	HI 90	MECVI
Default model	16.702	16.361	17.048	16.780
Saturated model	10.837	10.837	10.837	11.964
Independence model	69.497	68.734	70.265	69.516

HOELTER

Model	HOELTER .05	HOELTER .01
Default model	401	406
Independence model	98	99

Execution time summary

Minimization: 1.250
Miscellaneous: 6.357
Bootstrap: .000
Total: 7.607

Supplementary Information S3

Analysis of the Factorial Structure and Reliability of the Social Determinants of Mental Health Questionnaire for Young Adults (SDMH)

Mónica Roncancio—Moreno. Rita Patricia Ocampo—Cepeda. Walther M. Zúñiga and Arcadio de Jesús Cardona—Isaza

"Invariance Analysis".

C:\Users\media\Dropbox\Mi PC (LAPTOP—LCRG33R5)\Desktop\2024\Articulos 2024\Salud mental 2024 1\Analisis adicional noviembre\AFC TOTAL — Invarianza\AFCTOTAL.amw

Analysis Summary

Date and Time

Date: martes. 17 de diciembre de 2024
Time: 4:11:47 a. m.

Title

Afctotal: martes. 17 de diciembre de 2024 4:11 a. m.

Groups

Group number 1 (Group number 1)

Notes for Group (Group number 1)

The model is recursive.
Sample size = 576

Variable Summary (1 Females)

Your model contains the following variables (1 Females)

Variable counts (1 Females)

Number of variables in your model:	251
Number of observed variables:	115
Number of unobserved variables:	136
Number of exogenous variables:	136
Number of endogenous variables:	115

Parameter Summary (1 Females)

	Weights	Covariances	Variances	Means	Intercepts	Total
Fixed	136	0	0	136	0	272
Labeled	94	229	136	0	115	574
Unlabeled	0	0	0	0	0	0
Total	230	229	136	136	115	846

Variable counts (2 Males)

Number of variables in your model:	251

Number of observed variables: 115
Number of unobserved variables: 136
Number of exogenous variables: 136
Number of endogenous variables: 115

Parameter Summary (2 Males)

	Weights	Covariances	Variances	Means	Intercepts	Total
Fixed	136	0	0	136	0	272
Labeled	94	229	136	0	115	574
Unlabeled	0	0	0	0	0	0
Total	230	229	136	136	115	846

Result (Unconstrained)

Minimum was achieved
Chi—square = 27071.444
Degrees of freedom = 12422
Probability level = 0.000

1 Females (1 Females — Unconstrained)

Estimates (1 Females — Unconstrained)

Scalar Estimates (1 Females — Unconstrained)

Standardized Regression Weights: (1 Females — Unconstrained)

			Estimate
APOYCOM	<———	APOY	.712
APOYBIE	<———	APOY	.733
APOYDOC	<———	APOY	.784
APOTTRAB	<———	APOY	.284
APOYCOMP	<———	APOY	.512
APOYAMIG	<———	APOY	.281
APOYFAM	<———	APOY	.184
APOYPAR	<———	APOY	.065
AMIGOS	<———	HAB	.420
COMFAM	<———	HAB	.370
GRUPOS	<———	HAB	.504
RECREA	<———	HAB	.822
CULTUR	<———	HAB	.795
DEPORT	<———	HAB	.420
DORMIR	<———	HAB	.115
COMER	<———	HAB	.245
FORTALZ	<———	HP	.609
EMPATI	<———	HP	.440
EXPRSN	<———	HP	.691
INTERACT	<———	HP	.685
DECISNS	<———	HP	.713
AFRONT	<———	HP	.754
INNOVA	<———	HP	.772
CUESTIN	<———	HP	.678
EXPRESEMOC	<———	HP	.709
INDENTIF	<———	HP	.735
ENTHG	<———	HOG	.663
RELHOG	<———	HOG	.592
CONFLHOG	<———	HOG	.607
SOLHGR	<———	HOG	.523
ENTTBA	<———	BAR	.500
RELIBAR	<———	BAR	.663

			Estimate
CONFBARR	<---	BAR	.492
SOLBAR	<---	BAR	.462
ENTUNI	<---	ESC	.556
RELUNIVE	<---	ESC	.481
CONFCUNI	<---	ESC	.313
SOLUNIV	<---	ESC	.323
ENTTRAB	<---	LAB	.894
RELIOTRB	<---	LAB	.955
CONFTRA	<---	LAB	.867
SOLTRAB	<---	LAB	.754
ENTREL	<---	REL	.817
RELREL	<---	REL	.922
CONFREL	<---	REL	.762
SOLREL	<---	REL	.618
ENTHOB	<---	CUL	.920
RELHOB	<---	CUL	.945
CONFHOB	<---	CUL	.813
SOLHOB	<---	CUL	.654
MALESSLD	<---	MALS	.574
MALFINAZ	<---	MALS	.636
MALSOCL	<---	MALS	.605
MALFAM	<---	MALS	.711
MALPAR	<---	MALS	.322
DAÑO	<---	SUI	.608
INTENT	<---	SUI	.458
IDEACI	<---	SUI	.625
COMNERGZ	<---	DRUG	.485
COMMDIC	<---	DRUG	.404
CONSDRG	<---	DRUG	.650
CONTABAC	<---	DRUG	.594
CONSALCH	<---	DRUG	.603
PROLGLS	<---	PDRUG	.885
PROBACDM	<---	PDRUG	.908
PRMLABR	<---	PDRUG	.726
PROMECON	<---	PDRUG	.873
CONFAMIG	<---	PDRUG	.892
CONFPRJ	<---	PDRUG	.630
CONFFAM	<---	PDRUG	.880
RESDSLS	<---	USOINT	.073
APUESTAS	<---	USOINT	.450
PORN	<---	USOINT	.527
VIDEOJG	<---	USOINT	.622
CFLEGAL	<---	PROINT	.715
CFACADM	<---	PROINT	.657
CFLABR	<---	PROINT	.617
CFECNM	<---	PROINT	.813
CFAMIGS	<---	PROINT	.838
CFPAREJA	<---	PROINT	.503
CFFAMILIA	<---	PROINT	.665
NERVS	<---	SESEM	.717
PRCUPC	<---	SESEM	.795
ZOZ	<---	SESEM	.771
TENSINE	<---	SESEM	.795
TRIT	<---	SESEM	.782
IRRIT	<---	SESEM	.757
NODISFR	<---	SESEM	.678
PERDDNT	<---	SESEM	.690

			Estimate
PROBLDI	<---	SFUSEM	.714
COMRM	<---	SFUSEM	.723
DOLCA	<---	SFUSEM	.773
DOLRMU	<---	SFUSEM	.743
PROBS	<---	SFUSEM	.682
OPTIM	<---	BIMS	.660
VITALI	<---	BIMS	.724
ALGR	<---	BIMS	.766
TRAQUIL	<---	BIMS	.598
MESNR	<---	SEMES	.819
MESP	<---	SEMES	.846
MESZZ	<---	SEMES	.831
MESTN	<---	SEMES	.816
MESTRI	<---	SEMES	.821
MESIR	<---	SEMES	.807
MESDI	<---	SEMES	.756
PERIN	<---	SEMES	.753
MPROS	<---	SFMES	.755
MDOLMU	<---	SFMES	.806
MCABE	<---	SFMES	.809
MCOME	<---	SFMES	.806
MDIGE	<---	SFMES	.785
MALEG	<---	BIEMS	.844
MILUSI	<---	BIEMS	.804
MEVIT	<---	BIEMS	.749
MESTR	<---	BIEMS	.692

Standardized Regression Weights: (2 Males — Unconstrained)

			Estimate
APOYCOM	<---	APOY	.679
APOYBIE	<---	APOY	.737
APOYDOC	<---	APOY	.800
APOTTRAB	<---	APOY	.333
APOYCOMP	<---	APOY	.551
APOYAMIG	<---	APOY	.321
APOYFAM	<---	APOY	.381
APOYPAR	<---	APOY	.053
AMIGOS	<---	HAB	.449
COMFAM	<---	HAB	.393
GRUPOS	<---	HAB	.462
RECREA	<---	HAB	.862
CULTUR	<---	HAB	.755
DEPORT	<---	HAB	.503
DORMIR	<---	HAB	.159
COMER	<---	HAB	.223
FORTALZ	<---	HP	.654
EMPATI	<---	HP	.520
EXPRSN	<---	HP	.670
INTERACT	<---	HP	.691
DECISNS	<---	HP	.723
AFRONT	<---	HP	.737
INNOVA	<---	HP	.733
CUESTIN	<---	HP	.665

			Estimate
EXPRESEMOC	<---	HP	.693
INDENTIF	<---	HP	.696
ENTHG	<---	HOG	.724
RELHOG	<---	HOG	.709
CONFLHOG	<---	HOG	.569
SOLHGR	<---	HOG	.560
ENTTBA	<---	BAR	.419
RELIBAR	<---	BAR	.731
CONFBARR	<---	BAR	.371
SOLBAR	<---	BAR	.529
ENTUNI	<---	ESC	.489
RELUNIVE	<---	ESC	.500
CONFCUNI	<---	ESC	.375
SOLUNIV	<---	ESC	.319
ENTTRAB	<---	LAB	.889
RELIOTRB	<---	LAB	.947
CONFTRA	<---	LAB	.856
SOLTRAB	<---	LAB	.761
ENTREL	<---	REL	.829
RELREL	<---	REL	.956
CONFREL	<---	REL	.763
SOLREL	<---	REL	.668
ENTHOB	<---	CUL	.899
RELHOB	<---	CUL	.970
CONFHOB	<---	CUL	.805
SOLHOB	<---	CUL	.677
MALESSLD	<---	MALS	.514
MALFINAZ	<---	MALS	.536
MALSOCL	<---	MALS	.470
MALFAM	<---	MALS	.735
MALPAR	<---	MALS	.254
DAÑO	<---	SUI	.619
INTENT	<---	SUI	.527
IDEACI	<---	SUI	.712
COMNERGZ	<---	DRUG	.476
COMMDIC	<---	DRUG	.304
CONSDRG	<---	DRUG	.468
CONTABAC	<---	DRUG	.422
CONSALCH	<---	DRUG	.741
PROLGLS	<---	PDRUG	.834
PROBACDM	<---	PDRUG	.889
PRMLABR	<---	PDRUG	.754
PROMECON	<---	PDRUG	.889
CONFAMIG	<---	PDRUG	.863
CONFPRJ	<---	PDRUG	.630
CONFFAM	<---	PDRUG	.829
RESDSLS	<---	USOINT	.127
APUESTAS	<---	USOINT	.316
PORN	<---	USOINT	.631
VIDEOJG	<---	USOINT	.666
CFLEGAL	<---	PROINT	.738
CFACADM	<---	PROINT	.632
CFLABR	<---	PROINT	.645
CFECNM	<---	PROINT	.854
CFAMIGS	<---	PROINT	.835
CFPAREJA	<---	PROINT	.525
CFFAMILIA	<---	PROINT	.623

			Estimate
NERVS	<---	SESEM	.665
PRCUPC	<---	SESEM	.728
ZOZ	<---	SESEM	.746
TENSINE	<---	SESEM	.793
TRIT	<---	SESEM	.779
IRRIT	<---	SESEM	.704
NODISFR	<---	SESEM	.679
PERDDNT	<---	SESEM	.672
PROBLDI	<---	SFUSEM	.645
COMRM	<---	SFUSEM	.650
DOLCA	<---	SFUSEM	.722
DOLRMU	<---	SFUSEM	.678
PROBS	<---	SFUSEM	.618
OPTIM	<---	BIMS	.663
VITALI	<---	BIMS	.731
ALGR	<---	BIMS	.814
TRAQUIL	<---	BIMS	.605
MESNR	<---	SEMES	.811
MESP	<---	SEMES	.840
MESZZ	<---	SEMES	.815
MESTN	<---	SEMES	.811
MESTRI	<---	SEMES	.808
MESIR	<---	SEMES	.749
MESDI	<---	SEMES	.745
PERIN	<---	SEMES	.732
MPROS	<---	SFMES	.705
MDOLMU	<---	SFMES	.805
MCABE	<---	SFMES	.790
MCOME	<---	SFMES	.755
MDIGE	<---	SFMES	.759
MALEG	<---	BIEMS	.813
MILUSI	<---	BIEMS	.767
MEVIT	<---	BIEMS	.811
MESTR	<---	BIEMS	.680

Model Fit Summary

CMIN

Model	NPAR	CMIN	*df*	*p*	CMIN/DF
Unconstrained	1148	27071.444	12422	.000	2.179
Measurement weights	1054	27185.077	12516	.000	2.172
Measurement intercepts	939	27331.152	12631	.000	2.164
Structural covariances	707	27653.760	12863	.000	2.150
Measurement residuals	574	27939.726	12996	.000	2.150
Saturated model	13570	.000	0		
Independence model	460	93264.302	13110	.000	7.114

Baseline Comparisons

Model	NFI Delta1	RFI rho1	IFI Delta2	TLI rho2	CFI
Unconstrained	.710	.694	.819	.807	.817
Measurement weights	.709	.695	.818	.808	.817
Measurement intercepts	.707	.696	.818	.810	.817
Structural covariances	.703	.698	.816	.812	.815
Measurement residuals	.700	.698	.814	.812	.814
Saturated model	1.000		1.000		1.000
Independence model	.000	.000	.000	.000	.000

Parsimony－Adjusted Measures

Model	PRATIO	PNFI	PCFI
Unconstrained	.948	.672	.774
Measurement weights	.955	.676	.780
Measurement intercepts	.963	.681	.787
Structural covariances	.981	.690	.800
Measurement residuals	.991	.694	.806
Saturated model	.000	.000	.000
Independence model	1.000	.000	.000

NCP

Model	NCP	LO 90	HI 90
Unconstrained	14649.444	14177.971	15128.235
Measurement weights	14669.077	14196.847	15148.628
Measurement intercepts	14700.152	14226.918	15180.711
Structural covariances	14790.760	14315.191	15273.661
Measurement residuals	14943.726	14465.685	15429.099
Saturated model	.000	.000	.000
Independence model	80154.302	79188.900	81126.298

FMIN

Model	FMIN	F0	LO 90	HI 90
Unconstrained	22.009	11.910	11.527	12.299
Measurement weights	22.102	11.926	11.542	12.316
Measurement intercepts	22.220	11.951	11.567	12.342
Structural covariances	22.483	12.025	11.638	12.418
Measurement residuals	22.715	12.149	11.761	12.544
Saturated model	.000	.000	.000	.000
Independence model	75.825	65.166	64.381	65.956

RMSEA

Model	RMSEA	LO 90	HI 90	PCLOSE
Unconstrained	.031	.030	.031	1.000
Measurement weights	.031	.030	.031	1.000
Measurement intercepts	.031	.030	.031	1.000
Structural covariances	.031	.030	.031	1.000
Measurement residuals	.031	.030	.031	1.000
Independence model	.071	.070	.071	.000

AIC

Model	AIC	BCC	BIC	CAIC
Unconstrained	29367.444	29904.660	30082.090	30535.090
Measurement weights	29293.077	29786.304	42924.803	43377.803
Measurement intercepts	29209.152	29648.564	43185.878	43638.878
Structural covariances	29067.760	29398.606	43740.486	44193.486
Measurement residuals	29087.726	29356.334	44159.452	44612.452
Saturated model	27140.000	33490.186	3223.726	3676.726
Independence model	94184.302	94399.562	109598.028	110051.028

ECVI

Model	ECVI	LO 90	HI 90	MECVI
Unconstrained	23.876	23.493	24.265	24.313

Model	ECVI	LO 90	HI 90	MECVI
Measurement weights	23.816	23.432	24.205	24.217
Measurement intercepts	23.747	23.363	24.138	24.105
Structural covariances	23.632	23.246	24.025	23.901
Measurement residuals	23.649	23.260	24.043	23.867
Saturated model	22.065	22.065	22.065	27.228
Independence model	76.573	75.788	77.363	76.748

HOELTER

Model	HOELTER .05	HOELTER .01
Unconstrained	578	583
Measurement weights	580	585
Measurement intercepts	582	587
Structural covariances	585	590
Measurement residuals	585	590
Independence model	178	179

Nested Model Comparisons

Assuming model Unconstrained to be correct:

Model	*df*	CMIN	*p*	NFI Delta−1	IFI Delta−2	RFI rho−1	TLI rho2
Measurement weights	94	113.633	.082	.001	.001	−.001	−.001
Measurement intercepts	209	259.708	.010	.003	.003	−.002	−.003
Structural covariances	441	582.316	.000	.006	.007	−.004	−.005
Measurement residuals	574	868.282	.000	.009	.011	−.004	−.005

Assuming model Measurement weights to be correct:

Model	*df*	CMIN	*p*	NFI Delta−1	IFI Delta−2	RFI rho−1	TLI rho2
Measurement intercepts	115	146.075	.027	.002	.002	−.001	−.001
Structural covariances	347	468.683	.000	.005	.006	−.003	−.004
Measurement residuals	480	754.649	.000	.008	.009	−.003	−.004

Assuming model Measurement intercepts to be correct:

Model	*df*	CMIN	*p*	NFI Delta−1	IFI Delta−2	RFI rho−1	TLI rho2
Structural covariances	232	322.608	.000	.003	.004	−.002	−.002
Measurement residuals	365	608.574	.000	.007	.008	−.002	−.002

Assuming model Structural covariances to be correct:

Model	*df*	CMIN	*p*	NFI Delta−1	IFI Delta−2	RFI rho−1	TLI rho2
Measurement residuals	133	285.966	.000	.003	.004	.000	.000

Execution time summary

Minimization:	3.548
Miscellaneous:	60.678
Bootstrap:	.000
Total:	64.226
